# Supplementary material for: Does the grassland ecological compensation policy improve the herders’ breeding technical efficiency in China?—Based on the parallel mediation effect model
Source: PLoS One. 2021 Apr 29;16(4):e0249990. doi: 10.1371/journal.pone.0249990 (PMC8084240; doi:10.1371/journal.pone.0249990)
Supplement: S1 Table — (DOCX) [file pone.0249990.s001.docx]

**S1 Table. Estimation results of parallel mediating effects model of the effect of GEPSP on the efficiency of breeding technology: small scales**

|  | Dependent variable: | | | |
| --- | --- | --- | --- | --- |
|  |  | | | |
|  | Larea | scale | farmstr | eff |
|  | | | | |
| scale |  |  |  | -0.044 |
|  |  |  |  | (0.028) |
| farmstr |  |  |  | -0.010 |
|  |  |  |  | (0.039) |
| Larea |  |  |  | 0.113^***^ |
|  |  |  |  | (0.016) |
| policy | 8.340^***^ | 0.221 | -0.282 | 3.712^***^ |
|  | (1.245) | (0.720) | (0.512) | (0.436) |
| inc | -0.0003 | 0.001 | 0.002^*^ | -0.002^**^ |
|  | (0.002) | (0.001) | (0.001) | (0.001) |
| incstr | 0.116 | 0.214 | -0.008 | 0.027 |
|  | (0.228) | (0.132) | (0.094) | (0.076) |
| price | -0.003 | 0.010^***^ | 0.00001 | -0.003^*^ |
|  | (0.006) | (0.003) | (0.002) | (0.002) |
| age | 0.004^**^ | -0.001 | 0.00000 | 0.001 |
|  | (0.002) | (0.001) | (0.001) | (0.001) |
| edu | -0.043^**^ | 0.009 | 0.001 | 0.023^***^ |
|  | (0.021) | (0.012) | (0.009) | (0.007) |
| lab | 0.021 | -0.003 | -0.010 | -0.006 |
|  | (0.016) | (0.009) | (0.007) | (0.005) |
| dis1 | 0.184^***^ | 0.023 | -0.036 | -0.046^*^ |
|  | (0.070) | (0.040) | (0.029) | (0.024) |
| dis2 | -0.089 | -0.043 | -0.145^***^ | 0.038 |
|  | (0.095) | (0.055) | (0.039) | (0.032) |
| Year | Yes | Yes | Yes | Yes |
| Firm | Yes | Yes | Yes | Yes |
| Constant | 462.768^***^ | 8.808 | -19.027 | 208.766^***^ |
|  | (78.445) | (45.363) | (32.276) | (27.163) |
|  | | | | |
| Adjusted R^2^ | 0.158 | 0.757 | 0.066 | 0.386 |
| F Statistic | 8.659^***^ | 128.495^***^ | 3.870^***^ | 21.149^***^ |
|  | | | | |
