# Supplementary material for: Does the grassland ecological compensation policy improve the herders’ breeding technical efficiency in China?—Based on the parallel mediation effect model
Source: PLoS One. 2021 Apr 29;16(4):e0249990. doi: 10.1371/journal.pone.0249990 (PMC8084240; doi:10.1371/journal.pone.0249990)
Supplement: S2 Table — (DOCX) [file pone.0249990.s002.docx]

**S2 Table. Results of parallel mediating effects model of the effect of GEPSP on the efficiency of breeding technology: large scales**

|  | Dependent variable: | | | |
| --- | --- | --- | --- | --- |
|  |  | | | |
|  | Larea | scale | farmstr | eff |
| scale |  |  |  | 0.046^***^ |
|  |  |  |  | (0.010) |
| farmstr |  |  |  | 0.030 |
|  |  |  |  | (0.042) |
| Larea |  |  |  | 0.060^***^ |
|  |  |  |  | (0.017) |
| policy | 5.607^***^ | 12.056^***^ | 1.204^**^ | 3.608^***^ |
|  | (1.145) | (1.954) | (0.469) | (0.448) |
| inc | 0.002 | -0.002 | 0.002^***^ | -0.003^***^ |
|  | (0.002) | (0.003) | (0.001) | (0.001) |
| incstr | 0.085 | 0.695 | -0.012 | 0.058 |
|  | (0.258) | (0.441) | (0.106) | (0.094) |
| price | 0.0004 | 0.0003 | 0.003 | -0.002 |
|  | (0.005) | (0.009) | (0.002) | (0.002) |
| age | 0.005^**^ | 0.002 | 0.0002 | 0.003^***^ |
|  | (0.002) | (0.003) | (0.001) | (0.001) |
| edu | -0.030 | -0.003 | -0.008 | -0.010 |
|  | (0.022) | (0.038) | (0.009) | (0.008) |
| lab | 0.023 | 0.024 | -0.012^*^ | 0.011^*^ |
|  | (0.017) | (0.029) | (0.007) | (0.006) |
| dis1 | 0.076 | -0.105 | -0.026 | -0.010 |
|  | (0.072) | (0.123) | (0.030) | (0.026) |
| dis2 | -0.077 | 0.046 | -0.131^***^ | 0.039 |
|  | (0.095) | (0.163) | (0.039) | (0.035) |
| Year | Yes | Yes | Yes | Yes |
| Firm | Yes | Yes | Yes | Yes |
| Constant | 309.106^***^ | 691.122^***^ | 68.389^**^ | 206.528^***^ |
|  | (73.329) | (125.100) | (30.035) | (28.216) |
|  | | | | |
| Adjusted R^2^ | 0.092 | 0.656 | 0.106 | 0.401 |
| F Statistic | 5.155^***^ | 79.216^***^ | 5.871^***^ | 22.601^***^ |
|  | | | | |
