# Supplementary material for: Does the grassland ecological compensation policy improve the herders’ breeding technical efficiency in China?—Based on the parallel mediation effect model
Source: PLoS One. 2021 Apr 29;16(4):e0249990. doi: 10.1371/journal.pone.0249990 (PMC8084240; doi:10.1371/journal.pone.0249990)
Supplement: S3 Table — (DOCX) [file pone.0249990.s003.docx]

**S3 Table. Initial data**

| **id** | **year** | **Y** | **in_area** | **in_lab** | **in_feed** | **in_other** | **policy** | **Larea** | **age** | **edu** | **inc** | **incstr** | **farmstr** | **dis1** | **dis2** | **price** | **lab** | **scale** |
| --- | --- | --- | --- | --- | --- | --- | --- | --- | --- | --- | --- | --- | --- | --- | --- | --- | --- | --- |
| 1 | 2010 | 9 | 12.72 | 4 | 120.38 | 500.34 | 0 | 1 | 32 | 1 | 1.7 | 14.00% | 65.00% | 0.42 | 0.35 | 110.44 | 4 | 0.02 |
| 2 | 2010 | 8 | 14.19 | 4 | 132.09 | 434.82 | 0 | 1 | 34 | 2 | 5.17 | 11.00% | 62.00% | 0.47 | 0.4 | 110.31 | 5 | 0.04 |
| 3 | 2010 | 15 | 31.5 | 5 | 181.7 | 422.5 | 0 | 0 | 31 | 1 | 5.2 | 15.00% | 71.00% | 0.98 | 0.37 | 112.07 | 2 | 0.05 |
| 4 | 2010 | 19 | 12.39 | 3 | 126.97 | 4553.86 | 0 | 1 | 35 | 3 | 4.34 | 0.00% | 58.00% | 0.45 | 0.43 | 110.65 | 2 | 0.03 |
| 5 | 2010 | 8 | 14.86 | 5 | 131.56 | 239.39 | 0 | 0 | 25 | 3 | 6.73 | 0.00% | 84.00% | 1.08 | 1.07 | 110.51 | 4 | 0.04 |
| 6 | 2010 | 22 | 30.69 | 4 | 188.9 | 497.49 | 0 | 0 | 45 | 1 | 10.53 | 0.00% | 54.00% | 0.47 | 0.53 | 111.80 | 2 | 0.04 |
| 7 | 2010 | 10 | 25.03 | 4 | 120.68 | 919.92 | 0 | 0 | 31 | 2 | 6.93 | 0.00% | 59.00% | 1.03 | 1.17 | 114.04 | 6 | 0.02 |
| 8 | 2010 | 24 | 12.19 | 4 | 139.35 | 548.93 | 0 | 0 | 27 | 3 | 4.72 | 7.00% | 49.00% | 0.64 | 0.81 | 111.39 | 3 | 0.02 |
| 9 | 2010 | 25 | 28.12 | 4 | 192.58 | 560.2 | 0 | 0 | 24 | 2 | 20.55 | 0.00% | 74.00% | 1.45 | 0.57 | 119.40 | 2 | 0.05 |
| 10 | 2010 | 19 | 28.95 | 5 | 183.35 | 641.96 | 0 | 0 | 23 | 2 | 9.91 | 0.00% | 79.00% | 0.48 | 0.32 | 117.16 | 2 | 0.05 |
| 11 | 2010 | 20 | 28.05 | 5 | 125.21 | 634.67 | 0 | 0 | 44 | 2 | 9.92 | 0.00% | 84.00% | 1.05 | 0.36 | 113.36 | 3 | 0.06 |
| 12 | 2010 | 24 | 26.23 | 3 | 131.67 | 252.94 | 0 | 0 | 26 | 3 | 6.74 | 0.00% | 59.00% | 1.07 | 1.06 | 117.16 | 3 | 0.02 |
| 13 | 2010 | 9 | 26.82 | 4 | 136.09 | 515.94 | 0 | 0 | 52 | 3 | 15.76 | 0.00% | 78.00% | 1.43 | 0.4 | 108.81 | 3 | 0.03 |
| 14 | 2010 | 14 | 13.75 | 4 | 130.58 | 163.81 | 0 | 0 | 44 | 5 | 4.22 | 9.00% | 40.00% | 0.39 | 0.47 | 108.41 | 4 | 0.07 |
| 15 | 2010 | 26 | 31.56 | 3 | 185.1 | 8758.64 | 0 | 0 | 23 | 2 | 36 | 0.00% | 38.00% | 0.72 | 0.85 | 113.16 | 3 | 0.04 |
| 16 | 2010 | 14 | 31.86 | 4 | 190.97 | 170.67 | 0 | 0 | 51 | 2 | 4.19 | 9.00% | 47.00% | 0.41 | 0.43 | 108.20 | 3 | 0.05 |
| 17 | 2010 | 20 | 24.47 | 4 | 120.18 | 502.54 | 0 | 0 | 25 | 3 | 1.72 | 12.00% | 70.00% | 0.43 | 0.34 | 109.97 | 3 | 0.05 |
| 18 | 2010 | 10 | 28.27 | 4 | 137.03 | 4755.89 | 0 | 0 | 43 | 3 | 5.48 | 0.00% | 60.00% | 0.43 | 0.4 | 111.39 | 2 | 0.07 |
| 19 | 2010 | 26 | 11.41 | 5 | 139.27 | 4523.77 | 0 | 0 | 25 | 3 | 4.22 | 0.00% | 100.00% | 0.47 | 0.33 | 108.95 | 2 | 0.02 |
| 20 | 2010 | 22 | 15.25 | 3 | 125.38 | 499.38 | 0 | 0 | 68 | 3 | 1.79 | 9.00% | 62.00% | 0.43 | 0.32 | 108.00 | 2 | 0.05 |
| 21 | 2010 | 18 | 25.66 | 4 | 126.12 | 1494.49 | 0 | 0 | 24 | 2 | 13.16 | 0.00% | 72.00% | 0.38 | 0.36 | 112.68 | 2 | 0.06 |
| 22 | 2010 | 20 | 28.53 | 4 | 125.01 | 529.96 | 0 | 1 | 51 | 3 | 15.8 | 0.00% | 80.00% | 1.34 | 0.42 | 113.70 | 4 | 0.03 |
| 23 | 2010 | 15 | 48.15 | 6 | 246 | 541.64 | 0 | 0 | 46 | 2 | 13.69 | 0.00% | 62.00% | 0.48 | 0.42 | 107.32 | 3 | 0.04 |
| 24 | 2010 | 31 | 50.49 | 7 | 248.74 | 185.47 | 0 | 0 | 25 | 2 | 5.49 | 11.00% | 57.00% | 0.45 | 0.43 | 119.40 | 3 | 0.05 |
| 25 | 2010 | 11 | 19.94 | 6 | 78.27 | 750.87 | 0 | 0 | 32 | 3 | 1.19 | 0.00% | 74.00% | 0.23 | 0.29 | 108.14 | 1 | 0.05 |
| 26 | 2010 | 12 | 24.53 | 4 | 120.9 | 518.88 | 0 | 0 | 51 | 2 | 10.53 | 0.00% | 43.00% | 0.5 | 0.39 | 111.73 | 3 | 0.03 |
| 27 | 2010 | 25 | 17.51 | 4 | 79.82 | 448.98 | 0 | 0 | 50 | 3 | 4.88 | 0.00% | 75.00% | 0.82 | 0.25 | 114.92 | 2 | 0.07 |
| 28 | 2010 | 29 | 19.17 | 5 | 97.75 | 460.8 | 0 | 0 | 67 | 3 | 3.65 | 0.00% | 55.00% | 0.97 | 0.43 | 112.34 | 4 | 0.03 |
| 29 | 2010 | 23 | 25.49 | 4 | 135 | 220.43 | 0 | 0 | 27 | 3 | 6.72 | 0.00% | 50.00% | 1.03 | 0.89 | 106.44 | 2 | 0.04 |
| 30 | 2010 | 29 | 26.97 | 5 | 123.03 | 235.64 | 0 | 0 | 45 | 3 | 6.64 | 0.00% | 85.00% | 0.94 | 1.14 | 110.24 | 3 | 0.06 |
| 31 | 2010 | 24 | 27.87 | 4 | 124.88 | 913.46 | 0 | 0 | 69 | 2 | 7.01 | 0.00% | 41.00% | 1.03 | 0.99 | 110.03 | 5 | 0.07 |
| 32 | 2010 | 29 | 50.31 | 6 | 248.24 | 1590.28 | 0 | 0 | 51 | 4 | 17.16 | 0.00% | 48.00% | 0.46 | 0.36 | 115.53 | 6 | 0.07 |
| 33 | 2010 | 17 | 50.69 | 7 | 239.89 | 397.83 | 0 | 0 | 26 | 5 | 5.12 | 12.00% | 63.00% | 0.47 | 0.35 | 116.75 | 2 | 0.09 |
| 34 | 2010 | 15 | 15.96 | 4 | 76.77 | 378.52 | 0 | 0 | 43 | 4 | 5.34 | 0.00% | 100.00% | 0.24 | 0.3 | 112.88 | 6 | 0.06 |
| 35 | 2010 | 24 | 28.49 | 4 | 192.43 | 505.61 | 0 | 0 | 35 | 2 | 1.71 | 9.00% | 62.00% | 0.45 | 0.37 | 113.36 | 3 | 0.05 |
| 36 | 2010 | 17 | 18.6 | 4 | 88.74 | 1895.73 | 0 | 1 | 37 | 3 | 16.68 | 0.00% | 50.00% | 0.48 | 0.42 | 116.75 | 5 | 0.05 |
| 37 | 2010 | 33 | 29.76 | 4 | 182.37 | 512.63 | 0 | 0 | 45 | 2 | 10.57 | 0.00% | 41.00% | 1.08 | 0.46 | 109.22 | 2 | 0.05 |
| 38 | 2010 | 26 | 24.48 | 4 | 133.39 | 312.57 | 0 | 1 | 62 | 4 | 17.09 | 0.00% | 85.00% | 0.71 | 0.65 | 114.58 | 3 | 0.04 |
| 39 | 2010 | 25 | 25.79 | 3 | 129.15 | 926.5 | 0 | 1 | 36 | 5 | 6.96 | 0.00% | 43.00% | 1.04 | 1.01 | 112.95 | 5 | 0.03 |
| 40 | 2010 | 30 | 16.46 | 5 | 87.89 | 512.05 | 0 | 0 | 43 | 3 | 3.71 | 8.00% | 35.00% | 0.62 | 0.76 | 108.00 | 2 | 0.05 |
| 41 | 2010 | 26 | 24.92 | 4 | 126.75 | 355.2 | 0 | 0 | 38 | 4 | 22.3 | 0.00% | 86.00% | 0.63 | 0.79 | 112.95 | 3 | 0.06 |
| 42 | 2010 | 24 | 26.74 | 5 | 126.32 | 650.55 | 0 | 0 | 46 | 2 | 9.81 | 0.00% | 76.00% | 0.42 | 0.32 | 113.77 | 3 | 0.05 |
| 43 | 2010 | 26 | 48.5 | 8 | 229.25 | 404.05 | 0 | 0 | 46 | 5 | 5.12 | 9.00% | 69.00% | 0.8 | 0.62 | 111.05 | 2 | 0.08 |
| 44 | 2010 | 33 | 47.76 | 8 | 243.22 | 531.07 | 0 | 0 | 48 | 3 | 2.29 | 11.00% | 60.00% | 0.44 | 0.32 | 109.83 | 4 | 0.03 |
| 45 | 2010 | 31 | 25.2 | 4 | 134.22 | 4755.78 | 0 | 0 | 65 | 3 | 5.49 | 0.00% | 68.00% | 0.47 | 0.33 | 115.80 | 2 | 0.02 |
| 46 | 2010 | 33 | 47.32 | 6 | 245.26 | 480.89 | 0 | 0 | 63 | 4 | 4.35 | 0.00% | 53.00% | 1.06 | 0.45 | 108.47 | 4 | 0.05 |
| 47 | 2010 | 37 | 26.07 | 4 | 122.64 | 552.91 | 0 | 0 | 23 | 3 | 20.57 | 0.00% | 87.00% | 1.44 | 0.36 | 115.46 | 2 | 0.09 |
| 48 | 2010 | 40 | 16.4 | 6 | 97.9 | 740.38 | 0 | 0 | 22 | 3 | 1.25 | 0.00% | 80.00% | 0.23 | 0.29 | 106.17 | 2 | 0.06 |
| 49 | 2010 | 20 | 47.59 | 6 | 235.56 | 535.53 | 0 | 0 | 27 | 3 | 2.23 | 16.00% | 98.00% | 0.96 | 0.45 | 112.27 | 4 | 0.04 |
| 50 | 2010 | 15 | 17.35 | 6 | 89.15 | 447.44 | 0 | 1 | 44 | 3 | 4.85 | 0.00% | 70.00% | 0.85 | 0.48 | 119.06 | 3 | 0.1 |
| 51 | 2010 | 41 | 26.52 | 4 | 116.72 | 547.73 | 0 | 1 | 24 | 3 | 20.56 | 0.00% | 70.00% | 1.45 | 0.51 | 110.37 | 3 | 0.04 |
| 52 | 2010 | 24 | 27.75 | 4 | 132.13 | 1577.68 | 0 | 0 | 45 | 2 | 17.2 | 0.00% | 45.00% | 0.47 | 0.41 | 120.41 | 6 | 0.05 |
| 53 | 2010 | 47 | 24.04 | 5 | 125.82 | 506.74 | 0 | 0 | 21 | 3 | 2.27 | 12.00% | 93.00% | 0.44 | 0.39 | 107.39 | 3 | 0.1 |
| 54 | 2010 | 25 | 24.16 | 3 | 116.48 | 503.19 | 0 | 1 | 47 | 2 | 13.79 | 0.00% | 100.00% | 0.95 | 0.42 | 113.09 | 2 | 0.09 |
| 55 | 2010 | 23 | 24.3 | 5 | 118.77 | 514.27 | 0 | 0 | 47 | 2 | 10.53 | 0.00% | 38.00% | 0.96 | 0.33 | 113.70 | 2 | 0.06 |
| 56 | 2010 | 46 | 23.84 | 4 | 120.51 | 350.87 | 0 | 0 | 45 | 4 | 22.34 | 0.00% | 66.00% | 0.74 | 0.84 | 109.29 | 3 | 0.09 |
| 57 | 2010 | 39 | 22.34 | 4 | 111.44 | 458.36 | 0 | 0 | 38 | 3 | 4.74 | 0.00% | 83.00% | 0.92 | 0.56 | 107.59 | 4 | 0.1 |
| 58 | 2010 | 56 | 10.3 | 3 | 48.16 | 454.77 | 0 | 0 | 43 | 3 | 3.72 | 0.00% | 53.00% | 0.82 | 0.58 | 108.47 | 2 | 0.07 |
| 59 | 2010 | 25 | 12.41 | 3 | 57.21 | 374.85 | 0 | 0 | 61 | 4 | 5.38 | 0.00% | 62.00% | 0.23 | 0.25 | 117.29 | 2 | 0.1 |
| 60 | 2010 | 24 | 15.65 | 4 | 65.93 | 422.15 | 0 | 0 | 60 | 3 | 3.69 | 0.00% | 60.00% | 0.88 | 0.51 | 113.63 | 4 | 0.11 |
| 61 | 2010 | 31 | 19.32 | 4 | 98.62 | 448.57 | 0 | 1 | 60 | 3 | 4.87 | 0.00% | 70.00% | 0.86 | 0.28 | 119.06 | 3 | 0.1 |
| 62 | 2010 | 48 | 20.08 | 3 | 98.9 | 773.72 | 0 | 0 | 36 | 3 | 1.58 | 0.00% | 100.00% | 0.27 | 0.22 | 109.56 | 1 | 0.08 |
| 63 | 2010 | 49 | 10.55 | 4 | 52.25 | 1777.9 | 0 | 0 | 30 | 5 | 12.76 | 0.00% | 33.00% | 0.41 | 0.36 | 111.87 | 5 | 0.11 |
| 64 | 2010 | 42 | 11.08 | 3 | 55.08 | 1767.8 | 0 | 1 | 42 | 5 | 12.76 | 0.00% | 73.00% | 0.48 | 0.32 | 117.23 | 5 | 0.1 |
| 65 | 2010 | 53 | 14.32 | 4 | 71.37 | 405.53 | 0 | 1 | 27 | 4 | 6.95 | 0.00% | 48.00% | 0.27 | 0.3 | 110.51 | 3 | 0.12 |
| 66 | 2010 | 58 | 20.21 | 3 | 113.68 | 729.91 | 0 | 0 | 50 | 3 | 1.3 | 0.00% | 100.00% | 0.27 | 0.22 | 116.75 | 2 | 0.09 |
| 67 | 2010 | 45 | 22.7 | 3 | 102.43 | 464.11 | 0 | 0 | 53 | 3 | 4.81 | 0.00% | 77.00% | 0.91 | 0.55 | 109.49 | 4 | 0.07 |
| 68 | 2010 | 55 | 14.86 | 4 | 66.59 | 376.3 | 0 | 0 | 39 | 4 | 5.34 | 0.00% | 100.00% | 0.24 | 0.27 | 109.70 | 1 | 0.09 |
| 69 | 2010 | 40 | 12.14 | 3 | 44.3 | 384.72 | 0 | 0 | 53 | 4 | 5.34 | 0.00% | 58.00% | 0.26 | 0.27 | 111.05 | 3 | 0.07 |
| 70 | 2010 | 64 | 13.83 | 4 | 69.72 | 381.18 | 0 | 0 | 50 | 4 | 6.93 | 0.00% | 100.00% | 0.23 | 0.27 | 105.83 | 2 | 0.03 |
| 71 | 2010 | 49 | 15.97 | 4 | 77.18 | 517.29 | 0 | 0 | 38 | 3 | 3.68 | 5.00% | 41.00% | 0.62 | 0.74 | 113.83 | 3 | 0.05 |
| 72 | 2010 | 50 | 17.6 | 4 | 78.64 | 380.74 | 0 | 0 | 54 | 4 | 6.98 | 0.00% | 59.00% | 0.26 | 0.28 | 113.83 | 3 | 0.1 |
| 73 | 2010 | 26 | 17.42 | 4 | 69.11 | 396.96 | 0 | 0 | 44 | 4 | 5.27 | 0.00% | 76.00% | 0.23 | 0.24 | 109.63 | 3 | 0.06 |
| 74 | 2010 | 51 | 9.58 | 5 | 52.46 | 362.12 | 0 | 0 | 43 | 4 | 5.28 | 0.00% | 53.00% | 0.24 | 0.23 | 117.23 | 3 | 0.11 |
| 75 | 2010 | 39 | 20.16 | 4 | 96.23 | 1237.19 | 0 | 1 | 48 | 3 | 18.34 | 0.00% | 60.00% | 1.06 | 0.43 | 109.29 | 3 | 0.12 |
| 76 | 2010 | 47 | 15.25 | 5 | 85.5 | 438.26 | 0 | 1 | 60 | 3 | 3.74 | 0.00% | 43.00% | 0.89 | 0.42 | 106.17 | 4 | 0.09 |
| 77 | 2010 | 32 | 20.52 | 3 | 104.36 | 459.78 | 0 | 0 | 42 | 4 | 4.34 | 0.00% | 46.00% | 1.08 | 0.37 | 105.83 | 4 | 0.09 |
| 78 | 2010 | 48 | 21.04 | 4 | 115.87 | 792.71 | 0 | 1 | 34 | 3 | 1.61 | 0.00% | 61.00% | 0.24 | 0.28 | 114.44 | 2 | 0.1 |
| 79 | 2010 | 57 | 15.52 | 4 | 70.5 | 427.07 | 0 | 0 | 36 | 3 | 3.73 | 0.00% | 54.00% | 0.86 | 0.26 | 108.61 | 3 | 0.11 |
| 80 | 2010 | 43 | 13.6 | 4 | 69.68 | 387.32 | 0 | 1 | 42 | 4 | 5.36 | 0.00% | 100.00% | 0.25 | 0.21 | 106.17 | 2 | 0.15 |
| 81 | 2010 | 26 | 19.31 | 3 | 95.12 | 9190.98 | 0 | 1 | 44 | 4 | 46.93 | 0.00% | 58.00% | 0.81 | 0.9 | 117.02 | 5 | 0.11 |
| 82 | 2010 | 25 | 20.96 | 3 | 89.74 | 1573.48 | 0 | 0 | 43 | 4 | 17.18 | 0.00% | 91.00% | 0.24 | 0.22 | 116.95 | 6 | 0.16 |
| 83 | 2010 | 37 | 19.75 | 2 | 107.02 | 4518.98 | 0 | 0 | 43 | 3 | 4.22 | 0.00% | 71.00% | 0.45 | 0.3 | 110.85 | 2 | 0.1 |
| 84 | 2010 | 66 | 10.15 | 4 | 61.73 | 1803.27 | 0 | 0 | 51 | 5 | 12.88 | 0.00% | 39.00% | 0.42 | 0.4 | 111.59 | 7 | 0.1 |
| 85 | 2010 | 59 | 10.88 | 4 | 51.42 | 365.77 | 0 | 0 | 62 | 4 | 5.25 | 0.00% | 64.00% | 0.24 | 0.25 | 111.39 | 3 | 0.1 |
| 86 | 2010 | 68 | 15.46 | 3 | 68.28 | 393.82 | 0 | 0 | 57 | 4 | 6.93 | 0.00% | 100.00% | 0.23 | 0.29 | 109.29 | 2 | 0.06 |
| 87 | 2010 | 41 | 13.84 | 4 | 72.53 | 1874.97 | 0 | 0 | 53 | 5 | 16.6 | 0.00% | 68.00% | 0.44 | 0.33 | 112.07 | 7 | 0.14 |
| 88 | 2010 | 62 | 18.27 | 2 | 88.98 | 941.55 | 0 | 0 | 55 | 5 | 9.05 | 0.00% | 63.00% | 0.92 | 0.83 | 105.90 | 5 | 0.1 |
| 89 | 2010 | 53 | 13.22 | 4 | 73.03 | 1874.71 | 0 | 0 | 55 | 5 | 16.62 | 0.00% | 62.00% | 0.43 | 0.4 | 115.73 | 7 | 0.05 |
| 90 | 2010 | 31 | 13.44 | 4 | 74.01 | 1876.39 | 0 | 1 | 23 | 5 | 16.6 | 0.00% | 52.00% | 0.49 | 0.4 | 112.07 | 6 | 0.11 |
| 91 | 2010 | 41 | 19.26 | 4 | 95.51 | 1571.26 | 0 | 1 | 57 | 4 | 17.09 | 0.00% | 100.00% | 0.26 | 0.29 | 120.35 | 7 | 0.11 |
| 92 | 2010 | 44 | 15.45 | 5 | 72.94 | 1213.34 | 0 | 1 | 47 | 3 | 14.08 | 0.00% | 88.00% | 1.06 | 0.31 | 117.29 | 2 | 0.1 |
| 93 | 2010 | 59 | 19.09 | 2 | 94.75 | 1556.9 | 0 | 0 | 48 | 3 | 8.43 | 0.00% | 100.00% | 0.6 | 0.56 | 108.88 | 3 | 0.07 |
| 94 | 2010 | 65 | 20.34 | 4 | 103.38 | 485.65 | 0 | 1 | 23 | 4 | 2.49 | 0.00% | 46.00% | 0.91 | 0.48 | 117.43 | 3 | 0.15 |
| 95 | 2010 | 64 | 15.96 | 5 | 78.16 | 463.26 | 0 | 0 | 54 | 3 | 4.79 | 0.00% | 71.00% | 0.86 | 0.43 | 111.39 | 3 | 0.12 |
| 96 | 2010 | 75 | 20.41 | 4 | 103.41 | 1576.18 | 0 | 1 | 64 | 4 | 17.23 | 0.00% | 77.00% | 0.94 | 0.51 | 112.41 | 6 | 0.17 |
| 97 | 2010 | 54 | 12.83 | 4 | 70.61 | 1896.32 | 0 | 1 | 23 | 5 | 16.73 | 0.00% | 56.00% | 0.41 | 0.36 | 115.39 | 6 | 0.11 |
| 98 | 2010 | 49 | 13.53 | 4 | 65.04 | 728.28 | 0 | 0 | 47 | 3 | 1.21 | 0.00% | 100.00% | 0.27 | 0.27 | 109.02 | 2 | 0.16 |
| 99 | 2010 | 61 | 12.61 | 4 | 78.36 | 542.73 | 0 | 0 | 51 | 3 | 3.6 | 14.00% | 25.00% | 0.64 | 0.67 | 108.00 | 2 | 0.09 |
| 100 | 2010 | 45 | 12.82 | 4 | 68.59 | 1781.03 | 0 | 0 | 54 | 5 | 12.78 | 0.00% | 38.00% | 0.44 | 0.33 | 112.07 | 6 | 0.18 |
| 101 | 2010 | 33 | 21.9 | 2 | 109.09 | 167.51 | 0 | 0 | 45 | 2 | 4.19 | 8.00% | 84.00% | 0.38 | 0.42 | 108.54 | 4 | 0.1 |
| 102 | 2010 | 38 | 22.81 | 2 | 100.99 | 246.92 | 0 | 0 | 44 | 3 | 5.18 | 0.00% | 45.00% | 0.92 | 0.96 | 106.24 | 3 | 0.15 |
| 103 | 2010 | 77 | 24.42 | 3 | 113.59 | 4548.77 | 0 | 0 | 50 | 3 | 4.26 | 0.00% | 100.00% | 0.48 | 0.33 | 114.65 | 2 | 0.13 |
| 104 | 2010 | 73 | 23.04 | 3 | 101.16 | 233.95 | 0 | 0 | 50 | 3 | 5.2 | 0.00% | 48.00% | 0.93 | 0.86 | 115.67 | 3 | 0.11 |
| 105 | 2010 | 34 | 22.12 | 3 | 101.77 | 1208.93 | 0 | 1 | 45 | 3 | 14.06 | 0.00% | 73.00% | 0.98 | 0.48 | 112.14 | 3 | 0.16 |
| 106 | 2010 | 64 | 23.83 | 3 | 120.58 | 223.2 | 0 | 0 | 52 | 3 | 5.19 | 0.00% | 48.00% | 1.1 | 0.97 | 112.14 | 4 | 0.18 |
| 107 | 2010 | 53 | 21.8 | 3 | 119.7 | 1236.8 | 0 | 1 | 46 | 3 | 18.38 | 0.00% | 73.00% | 0.95 | 0.31 | 114.24 | 2 | 0.17 |
| 108 | 2010 | 66 | 21.44 | 3 | 101.71 | 1183.47 | 0 | 0 | 55 | 3 | 14.1 | 0.00% | 71.00% | 0.92 | 0.4 | 112.14 | 3 | 0.21 |
| 109 | 2010 | 85 | 20.21 | 3 | 119.6 | 236.86 | 0 | 0 | 26 | 3 | 5.22 | 0.00% | 48.00% | 0.97 | 1.08 | 108.61 | 3 | 0.12 |
| 110 | 2010 | 45 | 23.25 | 3 | 114.81 | 787.6 | 0 | 0 | 64 | 3 | 1.65 | 0.00% | 100.00% | 0.27 | 0.27 | 116.14 | 3 | 0.14 |
| 111 | 2010 | 42 | 22.77 | 2 | 115.42 | 536.68 | 0 | 0 | 40 | 3 | 4.69 | 13.00% | 55.00% | 0.62 | 0.65 | 119.53 | 2 | 0.16 |
| 112 | 2010 | 75 | 24.09 | 3 | 116.35 | 1239.37 | 0 | 1 | 45 | 3 | 18.36 | 0.00% | 65.00% | 0.95 | 0.3 | 113.83 | 4 | 0.12 |
| 113 | 2010 | 140 | 5.4 | 5 | 36.75 | 161.09 | 0 | 0 | 71 | 2 | 4.24 | 7.00% | 75.00% | 0.4 | 0.51 | 111.32 | 3 | 0.14 |
| 114 | 2010 | 130 | 3.72 | 5 | 33.14 | 312.44 | 0 | 0 | 39 | 4 | 17.17 | 0.00% | 79.00% | 0.7 | 0.76 | 110.78 | 6 | 0.15 |
| 115 | 2010 | 75 | 6 | 4 | 24.02 | 1497.41 | 0 | 0 | 37 | 4 | 13.16 | 0.00% | 57.00% | 0.27 | 0.22 | 118.11 | 6 | 0.2 |
| 116 | 2010 | 104 | 5.26 | 4 | 18.95 | 552.3 | 0 | 0 | 31 | 3 | 4.84 | 14.00% | 52.00% | 0.71 | 0.78 | 117.56 | 5 | 0.19 |
| 117 | 2010 | 130 | 3.89 | 4 | 32.6 | 339.9 | 0 | 0 | 38 | 4 | 17.18 | 0.00% | 74.00% | 0.66 | 0.85 | 112.48 | 5 | 0.26 |
| 118 | 2010 | 99 | 7.15 | 6 | 28.01 | 4554.65 | 0 | 0 | 56 | 3 | 4.28 | 0.00% | 56.00% | 0.46 | 0.36 | 114.58 | 4 | 0.2 |
| 119 | 2010 | 76 | 5.81 | 5 | 28.66 | 177.91 | 0 | 0 | 49 | 2 | 4.29 | 7.00% | 58.00% | 0.46 | 0.43 | 114.65 | 5 | 0.26 |
| 120 | 2010 | 144 | 5.34 | 4 | 23.61 | 527.32 | 0 | 0 | 45 | 3 | 15.78 | 0.00% | 76.00% | 1.36 | 0.49 | 107.19 | 2 | 0.23 |
| 121 | 2010 | 166 | 11.72 | 4 | 48.59 | 528.19 | 0 | 0 | 26 | 3 | 3.74 | 7.00% | 37.00% | 0.63 | 0.57 | 112.95 | 3 | 0.36 |
| 122 | 2010 | 125 | 13.11 | 4 | 50.83 | 378.52 | 0 | 0 | 42 | 4 | 6.92 | 0.00% | 100.00% | 0.25 | 0.23 | 117.50 | 1 | 0.27 |
| 123 | 2010 | 90 | 10.8 | 3 | 64.83 | 1795.52 | 0 | 1 | 64 | 5 | 12.83 | 0.00% | 68.00% | 0.47 | 0.34 | 111.80 | 5 | 0.17 |
| 124 | 2010 | 81 | 11.91 | 4 | 48.24 | 759.51 | 0 | 1 | 55 | 3 | 1.19 | 0.00% | 86.00% | 0.27 | 0.22 | 107.80 | 3 | 0.22 |
| 125 | 2010 | 85 | 11.42 | 4 | 65.89 | 1771.8 | 0 | 0 | 21 | 5 | 12.76 | 0.00% | 27.00% | 0.44 | 0.4 | 110.71 | 5 | 0.2 |
| 126 | 2010 | 104 | 19.62 | 5 | 96.2 | 554.41 | 0 | 0 | 46 | 3 | 4.78 | 11.00% | 46.00% | 0.73 | 0.71 | 106.71 | 3 | 0.29 |
| 127 | 2010 | 180 | 18.59 | 5 | 110.17 | 564.66 | 0 | 1 | 40 | 1 | 4.78 | 13.00% | 57.00% | 0.72 | 0.63 | 106.64 | 4 | 0.15 |
| 128 | 2010 | 135 | 10.71 | 4 | 50.03 | 761.03 | 0 | 0 | 50 | 3 | 1.29 | 0.00% | 72.00% | 0.28 | 0.28 | 115.33 | 2 | 0.26 |
| 129 | 2010 | 172 | 9.77 | 5 | 55.73 | 549.71 | 0 | 0 | 45 | 3 | 3.7 | 12.00% | 51.00% | 0.73 | 0.81 | 107.39 | 3 | 0.37 |
| 130 | 2010 | 138 | 11.18 | 5 | 49.66 | 450.88 | 0 | 1 | 32 | 3 | 3.75 | 0.00% | 42.00% | 0.89 | 0.26 | 108.68 | 3 | 0.24 |
| 131 | 2010 | 140 | 20.53 | 4 | 92.92 | 1176.98 | 0 | 0 | 50 | 3 | 14.13 | 0.00% | 78.00% | 0.96 | 0.29 | 107.80 | 4 | 0.22 |
| 132 | 2010 | 192 | 21.74 | 4 | 104.65 | 927.74 | 0 | 0 | 56 | 5 | 6.99 | 0.00% | 40.00% | 1.05 | 1.13 | 114.24 | 5 | 0.18 |
| 133 | 2010 | 134 | 18.74 | 5 | 112.74 | 221 | 0 | 0 | 38 | 3 | 5.09 | 0.00% | 44.00% | 0.93 | 1.06 | 114.44 | 2 | 0.19 |
| 134 | 2010 | 94 | 19.19 | 4 | 104.06 | 578.11 | 0 | 1 | 33 | 3 | 4.72 | 15.00% | 52.00% | 0.63 | 0.59 | 112.68 | 3 | 0.4 |
| 135 | 2010 | 177 | 21.37 | 4 | 100.51 | 1505.04 | 0 | 1 | 20 | 4 | 13.25 | 0.00% | 52.00% | 0.27 | 0.22 | 111.32 | 6 | 0.43 |
| 136 | 2010 | 153 | 18.38 | 4 | 102.19 | 477.69 | 0 | 0 | 39 | 3 | 1.8 | 14.00% | 73.00% | 1.03 | 0.98 | 115.33 | 3 | 0.26 |
| 137 | 2010 | 143 | 32.92 | 4 | 156.33 | 484.52 | 0 | 0 | 36 | 3 | 1.83 | 17.00% | 64.00% | 0.46 | 0.37 | 110.03 | 2 | 0.17 |
| 138 | 2010 | 78 | 31.92 | 3 | 169.84 | 245.07 | 0 | 0 | 30 | 3 | 6.67 | 0.00% | 52.00% | 1.07 | 0.94 | 109.02 | 3 | 0.33 |
| 139 | 2010 | 179 | 31.41 | 5 | 169.32 | 560.62 | 0 | 0 | 45 | 3 | 4.8 | 16.00% | 49.00% | 0.65 | 0.65 | 107.66 | 3 | 0.22 |
| 140 | 2010 | 107 | 30.23 | 4 | 152.63 | 248.94 | 0 | 0 | 44 | 3 | 5.15 | 0.00% | 52.00% | 0.91 | 0.95 | 114.44 | 3 | 0.38 |
| 141 | 2010 | 145 | 30.74 | 4 | 154.83 | 557.44 | 0 | 0 | 33 | 3 | 15.82 | 0.00% | 85.00% | 1.43 | 0.41 | 107.86 | 3 | 0.21 |
| 142 | 2010 | 205 | 31.44 | 3 | 149.02 | 1505.35 | 0 | 0 | 33 | 4 | 13.13 | 0.00% | 64.00% | 0.9 | 0.52 | 114.24 | 6 | 0.36 |
| 143 | 2010 | 86 | 30.02 | 4 | 166.29 | 230.23 | 0 | 1 | 44 | 3 | 5.12 | 0.00% | 56.00% | 0.93 | 0.99 | 113.36 | 2 | 0.26 |
| 144 | 2010 | 122 | 31.61 | 5 | 164.64 | 1490.38 | 0 | 0 | 52 | 4 | 13.26 | 0.00% | 60.00% | 0.26 | 0.28 | 108.81 | 6 | 0.4 |
| 145 | 2010 | 187 | 33.53 | 4 | 152.19 | 207.79 | 0 | 0 | 27 | 3 | 5.15 | 0.00% | 65.00% | 0.98 | 1.19 | 105.90 | 2 | 0.32 |
| 146 | 2010 | 149 | 29.79 | 5 | 149.17 | 1491.57 | 0 | 0 | 24 | 1 | 13.18 | 0.00% | 57.00% | 0.25 | 0.29 | 110.92 | 5 | 0.36 |
| 147 | 2010 | 208 | 30.78 | 5 | 170.34 | 4549.01 | 0 | 0 | 52 | 3 | 4.35 | 0.00% | 100.00% | 0.49 | 0.4 | 110.31 | 3 | 0.27 |
| 148 | 2010 | 94 | 32.68 | 4 | 163.33 | 329.26 | 0 | 0 | 50 | 4 | 17.21 | 0.00% | 55.00% | 0.65 | 0.63 | 108.00 | 3 | 0.37 |
| 149 | 2010 | 195 | 24.49 | 4 | 126.04 | 1498.43 | 0 | 0 | 37 | 4 | 13.2 | 0.00% | 56.00% | 0.98 | 0.47 | 108.95 | 7 | 0.37 |
| 150 | 2010 | 193 | 22.76 | 3 | 130.02 | 886.15 | 0 | 1 | 57 | 2 | 7.03 | 0.00% | 50.00% | 1.06 | 1.14 | 111.12 | 6 | 0.31 |
| 151 | 2010 | 103 | 25.96 | 5 | 118.46 | 900.47 | 0 | 0 | 25 | 5 | 7.05 | 0.00% | 50.00% | 1.07 | 1.22 | 116.41 | 6 | 0.4 |
| 152 | 2010 | 129 | 26.43 | 5 | 114.24 | 1515.62 | 0 | 0 | 28 | 4 | 13.24 | 0.00% | 68.00% | 0.86 | 0.59 | 116.48 | 6 | 0.33 |
| 153 | 2010 | 179 | 22.65 | 4 | 120.01 | 1529.74 | 0 | 0 | 47 | 4 | 13.22 | 0.00% | 71.00% | 0.86 | 0.54 | 118.72 | 6 | 0.47 |
| 154 | 2010 | 204 | 22.84 | 4 | 113.79 | 4552.36 | 0 | 0 | 30 | 3 | 4.33 | 0.00% | 100.00% | 0.49 | 0.3 | 109.08 | 2 | 0.46 |
| 155 | 2010 | 188 | 25.24 | 3 | 126.28 | 786.79 | 0 | 0 | 27 | 3 | 1.65 | 0.00% | 67.00% | 0.27 | 0.28 | 110.51 | 2 | 0.15 |
| 156 | 2010 | 95 | 24.74 | 5 | 130.65 | 1268.31 | 0 | 0 | 47 | 3 | 18.26 | 0.00% | 74.00% | 0.99 | 0.37 | 108.00 | 3 | 0.28 |
| 157 | 2010 | 141 | 12.41 | 3 | 65.52 | 504.13 | 0 | 0 | 27 | 3 | 1.83 | 11.00% | 73.00% | 0.41 | 0.41 | 106.85 | 3 | 0.36 |
| 158 | 2010 | 140 | 13.29 | 5 | 79.4 | 395.39 | 0 | 1 | 49 | 5 | 5.23 | 15.00% | 68.00% | 0.43 | 0.36 | 120.07 | 1 | 0.44 |
| 159 | 2010 | 88 | 13.9 | 4 | 69.85 | 249.33 | 0 | 0 | 70 | 3 | 6.63 | 0.00% | 44.00% | 1.03 | 1.06 | 116.95 | 3 | 0.4 |
| 160 | 2010 | 143 | 13.44 | 4 | 80.56 | 1564.14 | 0 | 0 | 31 | 4 | 17.22 | 0.00% | 87.00% | 0.27 | 0.22 | 113.83 | 5 | 0.27 |
| 161 | 2010 | 133 | 15.38 | 5 | 75.16 | 334.02 | 0 | 0 | 42 | 4 | 22.28 | 0.00% | 87.00% | 0.66 | 0.73 | 110.37 | 3 | 0.26 |
| 162 | 2010 | 143 | 12.01 | 4 | 82.04 | 532.67 | 0 | 0 | 37 | 3 | 15.84 | 0.00% | 77.00% | 1.45 | 0.53 | 115.05 | 4 | 0.25 |
| 163 | 2010 | 232 | 12.16 | 5 | 66.3 | 314.33 | 0 | 0 | 69 | 4 | 17.11 | 0.00% | 60.00% | 0.77 | 0.63 | 108.27 | 3 | 0.27 |
| 164 | 2010 | 141 | 15.18 | 4 | 65.11 | 337.01 | 0 | 0 | 44 | 4 | 17.14 | 0.00% | 89.00% | 0.71 | 0.69 | 116.34 | 3 | 0.39 |
| 165 | 2010 | 158 | 22.3 | 3 | 123.86 | 1629.69 | 0 | 1 | 69 | 3 | 8.15 | 0.00% | 49.00% | 0.52 | 0.6 | 116.75 | 3 | 0.39 |
| 166 | 2010 | 190 | 25.16 | 4 | 114.81 | 1635.15 | 0 | 0 | 68 | 3 | 8.07 | 0.00% | 43.00% | 0.52 | 0.58 | 115.60 | 3 | 0.37 |
| 167 | 2010 | 176 | 24.4 | 3 | 112.13 | 162.72 | 0 | 0 | 46 | 2 | 5.47 | 4.00% | 100.00% | 0.43 | 0.5 | 117.77 | 4 | 0.42 |
| 168 | 2010 | 189 | 21.84 | 3 | 109.56 | 470.35 | 0 | 0 | 35 | 4 | 4.45 | 0.00% | 53.00% | 0.49 | 0.35 | 113.63 | 3 | 0.26 |
| 169 | 2010 | 190 | 22.57 | 3 | 117.48 | 955.37 | 0 | 0 | 48 | 5 | 9.1 | 0.00% | 65.00% | 1.05 | 1.08 | 116.55 | 5 | 0.3 |
| 170 | 2010 | 200 | 21.22 | 4 | 123.77 | 456.84 | 0 | 0 | 62 | 4 | 2.5 | 0.00% | 72.00% | 0.83 | 0.34 | 110.78 | 4 | 0.31 |
| 171 | 2010 | 249 | 21.16 | 4 | 110 | 471.07 | 0 | 1 | 68 | 4 | 4.38 | 0.00% | 60.00% | 1.08 | 0.46 | 106.78 | 2 | 0.55 |
| 172 | 2010 | 280 | 24.98 | 3 | 116.94 | 536.37 | 0 | 0 | 43 | 3 | 2.23 | 18.00% | 57.00% | 0.49 | 0.43 | 108.81 | 3 | 0.23 |
| 173 | 2010 | 119 | 353.6 | 3 | 1753.16 | 1512.46 | 0 | 0 | 34 | 2 | 13.34 | 0.00% | 66.00% | 0.23 | 0.24 | 114.51 | 4 | 0.38 |
| 174 | 2010 | 117 | 355.19 | 4 | 1754.13 | 457.93 | 0 | 0 | 31 | 3 | 5.22 | 6.00% | 75.00% | 0.48 | 0.3 | 117.36 | 3 | 0.24 |
| 175 | 2010 | 242 | 357.23 | 5 | 1746.19 | 476.14 | 0 | 0 | 29 | 3 | 5.18 | 0.00% | 66.00% | 0.44 | 0.34 | 110.17 | 3 | 0.58 |
| 176 | 2010 | 259 | 357.28 | 4 | 1734.94 | 1510.92 | 0 | 0 | 31 | 2 | 13.35 | 0.00% | 79.00% | 0.89 | 0.26 | 109.63 | 2 | 0.57 |
| 177 | 2010 | 174 | 11.39 | 4 | 80.73 | 576.57 | 0 | 0 | 29 | 3 | 20.55 | 0.00% | 62.00% | 1.29 | 0.69 | 109.97 | 3 | 0.42 |
| 178 | 2010 | 267 | 354.98 | 4 | 1746.48 | 504.93 | 0 | 0 | 31 | 4 | 3.16 | 0.00% | 61.00% | 0.97 | 0.52 | 117.02 | 2 | 0.33 |
| 179 | 2010 | 138 | 14.41 | 5 | 76.79 | 357.7 | 0 | 0 | 32 | 4 | 22.25 | 0.00% | 83.00% | 0.67 | 0.69 | 111.32 | 4 | 0.65 |
| 180 | 2010 | 305 | 15.01 | 4 | 60.78 | 4766.03 | 0 | 0 | 30 | 3 | 5.59 | 0.00% | 66.00% | 0.48 | 0.33 | 113.56 | 3 | 0.4 |
| 181 | 2010 | 319 | 354.73 | 5 | 1747.37 | 1548.09 | 0 | 0 | 40 | 2 | 13.31 | 0.00% | 82.00% | 0.4 | 0.5 | 111.53 | 2 | 0.58 |
| 182 | 2010 | 231 | 353.89 | 5 | 1749.11 | 854.12 | 0 | 0 | 44 | 2 | 25.8 | 0.00% | 81.00% | 0.64 | 0.75 | 119.53 | 2 | 0.48 |
| 183 | 2010 | 224 | 13.88 | 5 | 78.53 | 499.32 | 0 | 1 | 47 | 3 | 1.79 | 8.00% | 72.00% | 0.93 | 0.36 | 107.73 | 3 | 0.37 |
| 184 | 2010 | 168 | 354.2 | 5 | 1739.14 | 474.55 | 0 | 0 | 22 | 3 | 5.14 | 4.00% | 66.00% | 0.94 | 0.83 | 113.70 | 4 | 0.23 |
| 185 | 2010 | 155 | 12.94 | 4 | 78.62 | 503.95 | 0 | 0 | 40 | 3 | 1.69 | 14.00% | 76.00% | 0.92 | 0.86 | 116.68 | 3 | 0.24 |
| 186 | 2010 | 334 | 12.97 | 4 | 63.61 | 663.91 | 0 | 0 | 62 | 2 | 9.82 | 0.00% | 84.00% | 0.95 | 0.38 | 110.51 | 3 | 0.48 |
| 187 | 2010 | 300 | 11.78 | 3 | 74.94 | 512.53 | 0 | 0 | 43 | 3 | 1.74 | 9.00% | 75.00% | 0.49 | 0.4 | 107.32 | 4 | 0.58 |
| 188 | 2010 | 139 | 12.79 | 4 | 63.91 | 229.99 | 0 | 0 | 45 | 3 | 6.7 | 0.00% | 72.00% | 1.06 | 1.07 | 106.17 | 3 | 0.57 |
| 189 | 2010 | 209 | 9.48 | 4 | 117.72 | 448.96 | 0 | 0 | 34 | 3 | 3.7 | 0.00% | 48.00% | 0.96 | 0.26 | 114.10 | 3 | 0.29 |
| 190 | 2010 | 228 | 8.01 | 5 | 112.7 | 1213.95 | 0 | 0 | 49 | 3 | 14.12 | 0.00% | 79.00% | 1.01 | 0.37 | 109.97 | 2 | 0.35 |
| 191 | 2010 | 329 | 8.89 | 4 | 110.98 | 513.24 | 0 | 0 | 24 | 3 | 3.63 | 8.00% | 41.00% | 0.72 | 0.74 | 112.00 | 3 | 0.31 |
| 192 | 2010 | 156 | 9.12 | 4 | 118.27 | 1768.78 | 0 | 0 | 43 | 5 | 12.78 | 0.00% | 70.00% | 0.42 | 0.39 | 107.12 | 5 | 0.47 |
| 193 | 2010 | 181 | 8.7 | 4 | 124.51 | 1196.35 | 0 | 0 | 37 | 3 | 14.09 | 0.00% | 86.00% | 1.05 | 0.29 | 113.56 | 3 | 0.33 |
| 194 | 2010 | 288 | 10.11 | 4 | 109.11 | 417.61 | 0 | 0 | 33 | 4 | 6.97 | 0.00% | 62.00% | 0.23 | 0.28 | 114.99 | 3 | 0.7 |
| 195 | 2010 | 317 | 10.57 | 4 | 117.94 | 1791.17 | 0 | 0 | 33 | 5 | 12.89 | 0.00% | 59.00% | 0.47 | 0.36 | 115.94 | 7 | 0.34 |
| 196 | 2010 | 346 | 10.71 | 4 | 104.49 | 1879.91 | 0 | 0 | 35 | 5 | 16.65 | 0.00% | 47.00% | 0.42 | 0.37 | 107.39 | 6 | 0.52 |
| 197 | 2010 | 317 | 27.33 | 4 | 132.09 | 1234.43 | 0 | 0 | 48 | 3 | 18.28 | 0.00% | 58.00% | 1.04 | 0.34 | 116.28 | 3 | 0.48 |
| 198 | 2010 | 203 | 26.56 | 2 | 142.1 | 411.01 | 0 | 1 | 48 | 4 | 6.9 | 0.00% | 100.00% | 0.27 | 0.24 | 106.85 | 2 | 0.67 |
| 199 | 2010 | 310 | 24.97 | 3 | 127.89 | 1881.28 | 0 | 0 | 36 | 5 | 16.65 | 0.00% | 51.00% | 0.42 | 0.35 | 117.23 | 6 | 0.41 |
| 200 | 2010 | 205 | 24.84 | 3 | 130.13 | 738.66 | 0 | 0 | 49 | 3 | 1.25 | 0.00% | 67.00% | 0.25 | 0.24 | 110.24 | 3 | 0.31 |
| 201 | 2010 | 264 | 27.83 | 3 | 144.38 | 790.01 | 0 | 1 | 52 | 3 | 1.61 | 0.00% | 100.00% | 0.26 | 0.25 | 116.89 | 3 | 0.38 |
| 202 | 2010 | 166 | 28.43 | 2 | 129.56 | 545.25 | 0 | 0 | 37 | 3 | 3.7 | 7.00% | 41.00% | 0.67 | 0.67 | 108.68 | 2 | 0.25 |
| 203 | 2010 | 125 | 24.77 | 4 | 134.48 | 1250.08 | 0 | 0 | 47 | 3 | 18.3 | 0.00% | 64.00% | 0.92 | 0.4 | 116.00 | 4 | 0.38 |
| 204 | 2010 | 261 | 27.09 | 2 | 132.37 | 788.95 | 0 | 0 | 35 | 3 | 1.61 | 0.00% | 68.00% | 0.23 | 0.23 | 115.80 | 2 | 0.62 |
| 205 | 2010 | 240 | 28.54 | 4 | 141.6 | 1875.71 | 0 | 0 | 22 | 5 | 16.62 | 0.00% | 63.00% | 0.41 | 0.4 | 116.61 | 5 | 0.71 |
| 206 | 2010 | 135 | 26.57 | 3 | 133.2 | 517.38 | 0 | 1 | 50 | 3 | 3.62 | 12.00% | 35.00% | 0.69 | 0.66 | 110.51 | 2 | 0.69 |
| 207 | 2010 | 163 | 28.69 | 2 | 126.78 | 546.45 | 0 | 0 | 47 | 3 | 4.8 | 14.00% | 45.00% | 0.62 | 0.57 | 110.37 | 2 | 0.7 |
| 208 | 2010 | 143 | 28.06 | 3 | 132.06 | 778.4 | 0 | 0 | 34 | 3 | 1.6 | 0.00% | 100.00% | 0.27 | 0.27 | 119.60 | 3 | 0.51 |
| 209 | 2010 | 357 | 37.19 | 3 | 173.04 | 1506.78 | 0 | 0 | 25 | 4 | 13.21 | 0.00% | 71.00% | 0.44 | 0.4 | 112.14 | 7 | 0.46 |
| 210 | 2010 | 142 | 34.18 | 4 | 179.65 | 1495.53 | 0 | 0 | 43 | 4 | 13.22 | 0.00% | 63.00% | 0.44 | 0.4 | 110.24 | 6 | 0.86 |
| 211 | 2010 | 199 | 34.5 | 3 | 186.57 | 168.71 | 0 | 0 | 47 | 2 | 4.29 | 10.00% | 54.00% | 0.39 | 0.41 | 109.76 | 3 | 0.56 |
| 212 | 2010 | 372 | 37.74 | 4 | 194.26 | 1506.35 | 0 | 0 | 29 | 2 | 13.18 | 0.00% | 65.00% | 0.42 | 0.45 | 115.80 | 7 | 0.81 |
| 213 | 2010 | 334 | 38.24 | 4 | 190.84 | 659.39 | 0 | 0 | 42 | 2 | 9.9 | 0.00% | 80.00% | 1 | 0.37 | 119.40 | 3 | 0.41 |
| 214 | 2010 | 376 | 34.16 | 4 | 193.3 | 4772.03 | 0 | 0 | 50 | 3 | 5.61 | 0.00% | 70.00% | 0.48 | 0.36 | 119.12 | 3 | 0.81 |
| 215 | 2010 | 370 | 36.28 | 5 | 175.85 | 314.35 | 0 | 1 | 45 | 4 | 17.18 | 0.00% | 56.00% | 0.69 | 0.79 | 115.80 | 3 | 0.49 |
| 216 | 2010 | 307 | 36.48 | 5 | 179.76 | 499.19 | 0 | 0 | 48 | 2 | 10.57 | 0.00% | 50.00% | 0.98 | 0.43 | 113.09 | 3 | 0.76 |
| 217 | 2010 | 159 | 35.78 | 4 | 179.39 | 4518.35 | 0 | 0 | 51 | 3 | 4.34 | 0.00% | 60.00% | 0.44 | 0.43 | 112.41 | 4 | 0.59 |
| 218 | 2010 | 265 | 38.28 | 3 | 180.68 | 920.36 | 0 | 0 | 50 | 5 | 6.93 | 0.00% | 51.00% | 1.09 | 1.18 | 106.91 | 4 | 0.43 |
| 219 | 2010 | 198 | 37.12 | 4 | 182.98 | 164.29 | 0 | 1 | 48 | 2 | 4.19 | 2.00% | 74.00% | 0.4 | 0.4 | 108.34 | 3 | 0.36 |
| 220 | 2010 | 266 | 35.64 | 5 | 178.76 | 481.78 | 0 | 0 | 47 | 2 | 10.53 | 0.00% | 37.00% | 0.43 | 0.4 | 106.51 | 3 | 0.36 |
| 221 | 2010 | 363 | 13.59 | 5 | 101.01 | 483.8 | 0 | 1 | 32 | 3 | 5.26 | 4.00% | 81.00% | 0.25 | 0.21 | 116.89 | 2 | 0.6 |
| 222 | 2010 | 234 | 17 | 4 | 101.99 | 1613.19 | 0 | 1 | 45 | 3 | 11.03 | 0.00% | 80.00% | 0.23 | 0.24 | 114.17 | 2 | 0.48 |
| 223 | 2010 | 374 | 35.13 | 5 | 184.16 | 1539.03 | 0 | 0 | 53 | 3 | 8.39 | 0.00% | 100.00% | 0.63 | 0.49 | 114.10 | 3 | 0.51 |
| 224 | 2010 | 363 | 15.37 | 5 | 105.67 | 1517.71 | 0 | 1 | 44 | 2 | 13.4 | 0.00% | 82.00% | 0.89 | 0.28 | 115.94 | 3 | 0.61 |
| 225 | 2010 | 182 | 35.13 | 4 | 180.46 | 431.51 | 0 | 0 | 50 | 5 | 5.17 | 8.00% | 75.00% | 0.92 | 0.47 | 120.48 | 1 | 0.68 |
| 226 | 2010 | 405 | 36.78 | 5 | 171.19 | 962.31 | 0 | 0 | 48 | 1 | 9.11 | 0.00% | 65.00% | 1.09 | 1.2 | 115.39 | 5 | 0.35 |
| 227 | 2010 | 419 | 36.37 | 6 | 174.39 | 837.93 | 0 | 0 | 68 | 2 | 19.83 | 0.00% | 85.00% | 0.96 | 0.44 | 108.81 | 2 | 0.71 |
| 228 | 2010 | 381 | 36.04 | 6 | 169.62 | 1551.04 | 0 | 0 | 49 | 3 | 8.38 | 0.00% | 61.00% | 1.06 | 0.85 | 108.27 | 4 | 0.82 |
| 229 | 2010 | 257 | 31.24 | 5 | 434.49 | 391.64 | 0 | 0 | 36 | 2 | 11.88 | 27.00% | 55.00% | 0.23 | 0.24 | 111.05 | 0 | 0.76 |
| 230 | 2010 | 404 | 32.92 | 4 | 427.22 | 484.02 | 0 | 0 | 37 | 4 | 5.65 | 0.00% | 39.00% | 0.47 | 0.49 | 117.36 | 3 | 0.48 |
| 231 | 2010 | 182 | 35 | 6 | 176.7 | 835.25 | 0 | 0 | 35 | 2 | 19.91 | 0.00% | 77.00% | 1.06 | 0.47 | 119.80 | 2 | 0.68 |
| 232 | 2010 | 329 | 33.29 | 5 | 422.18 | 1613.37 | 0 | 0 | 37 | 3 | 10.93 | 0.00% | 64.00% | 1.01 | 1.11 | 115.60 | 3 | 0.36 |
| 233 | 2010 | 390 | 36.31 | 5 | 185.53 | 542.86 | 0 | 0 | 65 | 2 | 13.74 | 0.00% | 100.00% | 0.94 | 0.38 | 110.03 | 2 | 0.48 |
| 234 | 2010 | 364 | 14.52 | 3 | 99.51 | 1713.94 | 0 | 1 | 42 | 3 | 10.46 | 0.00% | 46.00% | 0.46 | 0.52 | 117.97 | 4 | 0.45 |
| 235 | 2010 | 349 | 32.78 | 6 | 435.49 | 387.18 | 0 | 0 | 63 | 2 | 11.81 | 28.00% | 55.00% | 0.55 | 0.63 | 115.67 | 2 | 0.87 |
| 236 | 2010 | 233 | 34.03 | 6 | 170.45 | 801.74 | 0 | 0 | 56 | 2 | 19.83 | 0.00% | 75.00% | 0.69 | 0.82 | 110.10 | 2 | 0.56 |
| 237 | 2010 | 408 | 16.62 | 3 | 96.75 | 475.31 | 0 | 0 | 36 | 4 | 5.69 | 0.00% | 47.00% | 0.51 | 0.43 | 105.96 | 3 | 0.82 |
| 238 | 2010 | 397 | 14.06 | 5 | 102.62 | 1637.04 | 0 | 0 | 37 | 3 | 10.98 | 0.00% | 60.00% | 0.96 | 0.89 | 113.36 | 3 | 0.33 |
| 239 | 2010 | 335 | 16.04 | 5 | 109.24 | 1541.36 | 0 | 1 | 46 | 2 | 13.39 | 0.00% | 78.00% | 0.42 | 0.43 | 109.15 | 3 | 0.55 |
| 240 | 2010 | 241 | 33.47 | 4 | 185.12 | 147.06 | 0 | 1 | 42 | 2 | 5.45 | 4.00% | 71.00% | 0.46 | 0.48 | 111.05 | 3 | 0.47 |
| 241 | 2010 | 377 | 31.69 | 5 | 434.69 | 386.55 | 0 | 0 | 35 | 2 | 11.75 | 32.00% | 39.00% | 0.57 | 0.52 | 119.74 | 1 | 0.57 |
| 242 | 2010 | 327 | 36.24 | 5 | 181.7 | 478.38 | 0 | 0 | 36 | 4 | 4.41 | 0.00% | 44.00% | 0.44 | 0.29 | 107.46 | 3 | 0.83 |
| 243 | 2010 | 267 | 36.28 | 4 | 179.89 | 541.66 | 0 | 0 | 35 | 3 | 2.24 | 14.00% | 93.00% | 1.03 | 0.33 | 110.92 | 3 | 0.73 |
| 244 | 2010 | 347 | 34.07 | 6 | 428.59 | 1682.59 | 0 | 0 | 57 | 3 | 10.52 | 0.00% | 55.00% | 0.48 | 0.59 | 109.08 | 4 | 0.45 |
| 245 | 2010 | 393 | 34.07 | 6 | 174.02 | 464.87 | 0 | 0 | 39 | 4 | 4.41 | 0.00% | 44.00% | 0.55 | 0.46 | 111.19 | 3 | 0.41 |
| 246 | 2010 | 398 | 33.75 | 6 | 417.19 | 1134.79 | 0 | 0 | 48 | 3 | 54.46 | 0.00% | 56.00% | 0.99 | 0.46 | 117.29 | 4 | 0.35 |
| 247 | 2010 | 379 | 32.28 | 5 | 434.95 | 1149.48 | 0 | 0 | 44 | 3 | 54.41 | 0.00% | 53.00% | 1.03 | 0.34 | 110.78 | 4 | 0.69 |
| 248 | 2010 | 336 | 14.96 | 5 | 106.45 | 1616.2 | 0 | 1 | 41 | 3 | 10.96 | 0.00% | 78.00% | 0.23 | 0.23 | 109.90 | 2 | 0.52 |
| 249 | 2010 | 198 | 31.76 | 6 | 435.86 | 1141.27 | 0 | 0 | 43 | 3 | 54.48 | 0.00% | 59.00% | 1.05 | 0.39 | 111.87 | 4 | 0.79 |
| 250 | 2010 | 249 | 33.77 | 6 | 434.25 | 368.91 | 0 | 0 | 46 | 2 | 11.77 | 26.00% | 65.00% | 0.25 | 0.26 | 109.08 | 2 | 1 |
| 251 | 2010 | 322 | 30.59 | 6 | 420.35 | 1637.06 | 0 | 0 | 22 | 3 | 10.94 | 0.00% | 86.00% | 0.62 | 0.67 | 117.63 | 4 | 0.78 |
| 252 | 2010 | 338 | 31.13 | 5 | 424.11 | 1688.16 | 0 | 0 | 56 | 3 | 10.51 | 0.00% | 37.00% | 0.52 | 0.41 | 115.73 | 3 | 0.97 |
| 253 | 2010 | 388 | 9.33 | 4 | 93.2 | 1144.23 | 0 | 0 | 42 | 3 | 54.42 | 0.00% | 61.00% | 1.05 | 0.38 | 114.58 | 2 | 0.8 |
| 254 | 2010 | 171 | 8.67 | 4 | 92.13 | 506.06 | 0 | 0 | 39 | 4 | 3.24 | 0.00% | 58.00% | 0.91 | 0.33 | 113.49 | 4 | 0.92 |
| 255 | 2010 | 397 | 12.72 | 5 | 92.05 | 510.76 | 0 | 0 | 44 | 4 | 3.27 | 0.00% | 75.00% | 0.87 | 0.28 | 110.31 | 4 | 0.84 |
| 256 | 2010 | 259 | 11.27 | 4 | 102.23 | 1149.15 | 0 | 0 | 30 | 3 | 54.52 | 0.00% | 60.00% | 1.1 | 0.36 | 112.54 | 2 | 0.88 |
| 257 | 2010 | 183 | 11.83 | 4 | 89.96 | 346.28 | 0 | 0 | 40 | 2 | 9.15 | 20.00% | 100.00% | 0.83 | 0.26 | 114.72 | 1 | 1.14 |
| 258 | 2010 | 194 | 22.77 | 3 | 131.76 | 768.88 | 0 | 0 | 46 | 3 | 1.58 | 0.00% | 53.00% | 0.23 | 0.22 | 114.04 | 1 | 0.64 |
| 259 | 2010 | 316 | 24.96 | 4 | 135.94 | 449.29 | 0 | 0 | 40 | 3 | 4.86 | 0.00% | 67.00% | 0.91 | 0.27 | 119.60 | 4 | 0.79 |
| 260 | 2010 | 173 | 9.28 | 6 | 85.76 | 1624.4 | 0 | 0 | 48 | 3 | 10.93 | 0.00% | 81.00% | 0.6 | 0.56 | 111.73 | 3 | 0.4 |
| 261 | 2010 | 220 | 10.37 | 6 | 95.27 | 452.21 | 0 | 0 | 46 | 5 | 6.69 | 16.00% | 85.00% | 0.97 | 0.37 | 115.12 | 2 | 0.8 |
| 262 | 2010 | 173 | 25.58 | 5 | 131.69 | 752.7 | 0 | 0 | 40 | 3 | 1.21 | 0.00% | 100.00% | 0.24 | 0.24 | 117.36 | 3 | 0.54 |
| 263 | 2010 | 372 | 11.12 | 5 | 84.63 | 431.14 | 0 | 0 | 47 | 5 | 6.75 | 17.00% | 73.00% | 0.69 | 0.86 | 110.51 | 2 | 0.58 |
| 264 | 2010 | 410 | 23.34 | 3 | 130.08 | 389.49 | 0 | 0 | 38 | 4 | 6.98 | 0.00% | 63.00% | 0.25 | 0.26 | 107.86 | 2 | 0.38 |
| 265 | 2010 | 415 | 24.11 | 5 | 127.15 | 425.81 | 0 | 0 | 32 | 3 | 3.7 | 0.00% | 43.00% | 0.88 | 0.28 | 109.56 | 3 | 0.88 |
| 266 | 2010 | 364 | 26.1 | 4 | 123.49 | 469.72 | 0 | 0 | 48 | 3 | 4.81 | 0.00% | 68.00% | 0.97 | 0.31 | 115.67 | 2 | 0.98 |
| 267 | 2010 | 386 | 22.03 | 3 | 127.17 | 1203.59 | 0 | 0 | 47 | 3 | 14.1 | 0.00% | 64.00% | 1.07 | 0.43 | 106.24 | 3 | 0.97 |
| 268 | 2010 | 420 | 26.15 | 5 | 125.97 | 1189.84 | 0 | 0 | 38 | 3 | 14.16 | 0.00% | 77.00% | 0.98 | 0.32 | 117.56 | 4 | 1 |
| 269 | 2010 | 359 | 14.8 | 5 | 73.35 | 1567.06 | 0 | 0 | 23 | 4 | 17.09 | 0.00% | 85.00% | 0.85 | 0.41 | 115.12 | 6 | 1.16 |
| 270 | 2010 | 314 | 16.2 | 4 | 81.62 | 473.33 | 0 | 0 | 25 | 4 | 4.33 | 0.00% | 58.00% | 0.49 | 0.56 | 110.92 | 3 | 0.84 |
| 271 | 2010 | 322 | 15.54 | 4 | 93.09 | 436.54 | 0 | 1 | 46 | 5 | 6.79 | 18.00% | 100.00% | 0.46 | 0.3 | 120.01 | 2 | 0.68 |
| 272 | 2010 | 184 | 16.93 | 4 | 77.88 | 1552.89 | 0 | 0 | 30 | 3 | 8.36 | 0.00% | 100.00% | 0.55 | 0.68 | 111.05 | 3 | 0.43 |
| 273 | 2010 | 479 | 16.04 | 5 | 77.71 | 472.35 | 0 | 0 | 26 | 4 | 4.43 | 0.00% | 49.00% | 0.47 | 0.53 | 110.71 | 2 | 0.95 |
| 274 | 2010 | 440 | 15.21 | 3 | 92.11 | 962.55 | 0 | 0 | 26 | 5 | 9.11 | 0.00% | 44.00% | 0.91 | 1.05 | 114.92 | 5 | 0.58 |
| 275 | 2010 | 536 | 13.99 | 5 | 81.69 | 1615.87 | 0 | 1 | 44 | 3 | 8.02 | 0.00% | 52.00% | 0.49 | 0.52 | 109.76 | 3 | 0.49 |
| 276 | 2010 | 515 | 16.84 | 5 | 78.53 | 516.9 | 0 | 0 | 31 | 3 | 2.2 | 11.00% | 78.00% | 0.45 | 0.36 | 116.55 | 3 | 1.08 |
| 277 | 2010 | 508 | 17.38 | 4 | 90.76 | 1548.82 | 0 | 1 | 29 | 3 | 8.49 | 0.00% | 100.00% | 0.65 | 0.57 | 117.29 | 2 | 0.67 |
| 278 | 2010 | 497 | 14.86 | 4 | 81.41 | 1620.81 | 0 | 1 | 30 | 3 | 8.1 | 0.00% | 36.00% | 0.54 | 0.6 | 117.56 | 4 | 0.84 |
| 279 | 2010 | 249 | 14.46 | 5 | 90.52 | 457.08 | 0 | 0 | 39 | 5 | 6.71 | 18.00% | 100.00% | 0.44 | 0.31 | 118.31 | 1 | 1.1 |
| 280 | 2010 | 467 | 14.38 | 4 | 82.1 | 337.2 | 0 | 0 | 45 | 2 | 9.12 | 28.00% | 100.00% | 0.24 | 0.26 | 108.81 | 4 | 0.84 |
| 281 | 2010 | 507 | 33.35 | 8 | 180.11 | 455.12 | 0 | 0 | 32 | 4 | 2.49 | 0.00% | 58.00% | 0.85 | 0.53 | 114.85 | 4 | 0.65 |
| 282 | 2010 | 361 | 35.26 | 6 | 185.38 | 458.78 | 0 | 0 | 44 | 4 | 4.37 | 0.00% | 62.00% | 0.49 | 0.42 | 114.24 | 3 | 0.73 |
| 283 | 2010 | 339 | 35.19 | 8 | 183.01 | 828.03 | 0 | 0 | 40 | 2 | 19.82 | 0.00% | 84.00% | 0.91 | 0.47 | 110.03 | 2 | 0.63 |
| 284 | 2010 | 385 | 33.36 | 7 | 184.25 | 532.63 | 0 | 0 | 40 | 3 | 2.2 | 17.00% | 51.00% | 0.48 | 0.31 | 106.78 | 3 | 0.55 |
| 285 | 2010 | 470 | 34.3 | 8 | 180.83 | 521.73 | 0 | 0 | 40 | 2 | 13.73 | 0.00% | 100.00% | 1.01 | 0.34 | 109.49 | 3 | 1.14 |
| 286 | 2010 | 289 | 35.45 | 7 | 171.67 | 473.47 | 0 | 0 | 39 | 4 | 2.46 | 0.00% | 56.00% | 0.96 | 0.47 | 113.02 | 5 | 0.84 |
| 287 | 2010 | 596 | 33 | 6 | 167.88 | 175.04 | 0 | 0 | 21 | 2 | 5.5 | 5.00% | 100.00% | 0.43 | 0.37 | 116.55 | 4 | 0.61 |
| 288 | 2010 | 419 | 35.84 | 7 | 189.36 | 543.82 | 0 | 0 | 41 | 3 | 20.55 | 0.00% | 70.00% | 1.36 | 0.6 | 119.26 | 2 | 1.14 |
| 289 | 2010 | 595 | 29.82 | 5 | 160.44 | 147.45 | 0 | 0 | 45 | 2 | 5.45 | 11.00% | 57.00% | 0.44 | 0.39 | 111.12 | 4 | 1.31 |
| 290 | 2010 | 261 | 29.5 | 6 | 167.56 | 515.94 | 0 | 0 | 26 | 3 | 1.78 | 13.00% | 73.00% | 1.1 | 0.37 | 112.27 | 3 | 1.18 |
| 291 | 2010 | 259 | 31.87 | 5 | 159.05 | 486.43 | 0 | 0 | 31 | 2 | 10.59 | 0.00% | 45.00% | 0.92 | 0.41 | 114.51 | 2 | 0.74 |
| 292 | 2010 | 584 | 30.2 | 5 | 153.67 | 4779.74 | 0 | 0 | 27 | 3 | 5.5 | 0.00% | 72.00% | 0.47 | 0.33 | 113.77 | 2 | 1.06 |
| 293 | 2010 | 496 | 31.39 | 5 | 161.59 | 335.05 | 0 | 0 | 32 | 4 | 22.3 | 0.00% | 65.00% | 0.69 | 0.65 | 114.38 | 3 | 0.96 |
| 294 | 2010 | 365 | 32.9 | 5 | 157.44 | 166.97 | 0 | 0 | 26 | 2 | 4.26 | 5.00% | 90.00% | 0.41 | 0.39 | 111.05 | 3 | 0.96 |
| 295 | 2010 | 359 | 29.03 | 5 | 166.92 | 549.43 | 0 | 0 | 26 | 3 | 20.58 | 0.00% | 74.00% | 1.37 | 0.48 | 115.33 | 3 | 1.06 |
| 296 | 2010 | 345 | 32.65 | 5 | 169.1 | 428.7 | 0 | 0 | 24 | 5 | 5.16 | 10.00% | 62.00% | 1 | 0.4 | 119.74 | 1 | 1.06 |
| 297 | 2010 | 443 | 71.93 | 5 | 351.71 | 1539.36 | 0 | 0 | 53 | 3 | 8.44 | 0.00% | 70.00% | 0.93 | 0.84 | 108.07 | 4 | 0.61 |
| 298 | 2010 | 291 | 70.17 | 4 | 349.34 | 534.48 | 0 | 0 | 38 | 3 | 2.22 | 11.00% | 56.00% | 0.97 | 0.85 | 114.44 | 4 | 0.45 |
| 299 | 2010 | 632 | 69.73 | 4 | 363.29 | 426.53 | 0 | 0 | 53 | 5 | 5.18 | 8.00% | 75.00% | 0.96 | 0.4 | 114.24 | 2 | 1.29 |
| 300 | 2010 | 243 | 73.28 | 5 | 358.13 | 663.78 | 0 | 0 | 52 | 2 | 9.92 | 0.00% | 82.00% | 0.42 | 0.31 | 110.58 | 4 | 1.34 |
| 301 | 2010 | 596 | 71.24 | 3 | 368.36 | 968.41 | 0 | 0 | 40 | 1 | 9.12 | 0.00% | 44.00% | 1.05 | 1.12 | 110.71 | 4 | 1.23 |
| 302 | 2010 | 266 | 72.5 | 4 | 349.41 | 408.71 | 0 | 0 | 51 | 2 | 5.12 | 9.00% | 69.00% | 0.68 | 0.66 | 117.29 | 1 | 0.87 |
| 303 | 2010 | 425 | 70.28 | 4 | 360.38 | 518.68 | 0 | 0 | 44 | 2 | 13.67 | 0.00% | 100.00% | 1.06 | 0.42 | 111.26 | 2 | 1.29 |
| 304 | 2010 | 279 | 71.9 | 4 | 347.58 | 687.28 | 0 | 0 | 41 | 2 | 12.91 | 0.00% | 73.00% | 0.45 | 0.34 | 110.98 | 4 | 1.38 |
| 305 | 2010 | 318 | 73.02 | 4 | 361.75 | 674.21 | 0 | 0 | 54 | 2 | 12.87 | 0.00% | 100.00% | 0.91 | 0.35 | 116.21 | 4 | 1.32 |
| 306 | 2010 | 501 | 72.78 | 5 | 351.52 | 1559.1 | 0 | 0 | 37 | 3 | 8.51 | 0.00% | 75.00% | 0.25 | 0.25 | 110.31 | 2 | 1.33 |
| 307 | 2010 | 614 | 72.85 | 3 | 348.23 | 1572.44 | 0 | 0 | 55 | 4 | 17.09 | 0.00% | 85.00% | 0.27 | 0.26 | 111.12 | 6 | 1.35 |
| 308 | 2010 | 462 | 69.78 | 4 | 353 | 1525.24 | 0 | 0 | 39 | 3 | 8.51 | 0.00% | 68.00% | 0.28 | 0.29 | 111.19 | 3 | 1.01 |
| 309 | 2010 | 642 | 44.9 | 4 | 235.6 | 521.16 | 0 | 0 | 38 | 2 | 10.56 | 0.00% | 44.00% | 0.93 | 0.4 | 112.95 | 3 | 0.66 |
| 310 | 2010 | 651 | 43.16 | 5 | 235.4 | 1539.88 | 0 | 0 | 21 | 3 | 8.5 | 0.00% | 58.00% | 0.98 | 1.22 | 109.49 | 2 | 1.08 |
| 311 | 2010 | 406 | 42.52 | 5 | 235.82 | 492.96 | 0 | 0 | 39 | 2 | 10.51 | 0.00% | 51.00% | 1.05 | 0.35 | 115.94 | 3 | 1.31 |
| 312 | 2010 | 415 | 44.14 | 4 | 232.92 | 402.63 | 0 | 0 | 38 | 5 | 5.26 | 14.00% | 69.00% | 0.43 | 0.37 | 110.92 | 3 | 1.48 |
| 313 | 2010 | 564 | 46.15 | 3 | 233.25 | 1598.16 | 0 | 0 | 20 | 4 | 17.2 | 0.00% | 45.00% | 0.38 | 0.45 | 117.63 | 7 | 0.7 |
| 314 | 2010 | 335 | 40.15 | 5 | 206.57 | 352.85 | 0 | 0 | 51 | 2 | 9.16 | 25.00% | 48.00% | 0.53 | 0.47 | 110.03 | 2 | 1.41 |
| 315 | 2010 | 436 | 44.96 | 5 | 231.49 | 952.57 | 0 | 0 | 20 | 5 | 9.05 | 0.00% | 62.00% | 1.03 | 1.05 | 109.83 | 5 | 0.81 |
| 316 | 2010 | 294 | 20.58 | 4 | 114.39 | 496.68 | 0 | 0 | 38 | 3 | 1.77 | 11.00% | 68.00% | 1.06 | 1.19 | 111.66 | 3 | 0.88 |
| 317 | 2010 | 642 | 42.8 | 5 | 212.08 | 1609.23 | 0 | 0 | 45 | 3 | 8.11 | 0.00% | 48.00% | 0.47 | 0.59 | 111.32 | 3 | 0.74 |
| 318 | 2010 | 447 | 43.44 | 3 | 207.33 | 1629.49 | 0 | 0 | 39 | 3 | 11.03 | 0.00% | 73.00% | 0.23 | 0.26 | 113.90 | 3 | 0.65 |
| 319 | 2010 | 288 | 23.13 | 4 | 114.55 | 671.27 | 0 | 0 | 29 | 2 | 9.92 | 0.00% | 82.00% | 1.03 | 0.33 | 117.29 | 4 | 0.95 |
| 320 | 2010 | 670 | 23.89 | 5 | 111.41 | 631.29 | 0 | 0 | 48 | 2 | 9.88 | 0.00% | 81.00% | 0.43 | 0.35 | 116.68 | 3 | 0.79 |
| 321 | 2010 | 485 | 44.63 | 5 | 228.99 | 530.96 | 0 | 0 | 50 | 2 | 13.75 | 0.00% | 100.00% | 1 | 0.37 | 116.00 | 3 | 0.61 |
| 322 | 2010 | 643 | 46.56 | 4 | 223.72 | 474.92 | 0 | 0 | 50 | 4 | 2.43 | 0.00% | 78.00% | 0.86 | 0.28 | 107.59 | 4 | 0.78 |
| 323 | 2010 | 289 | 23.38 | 4 | 120.07 | 232.58 | 0 | 0 | 29 | 3 | 6.7 | 0.00% | 72.00% | 0.94 | 1.12 | 108.54 | 4 | 0.92 |
| 324 | 2010 | 616 | 43.59 | 5 | 220.11 | 668.61 | 0 | 0 | 47 | 2 | 12.85 | 0.00% | 74.00% | 0.44 | 0.35 | 113.97 | 2 | 1.58 |
| 325 | 2010 | 689 | 24.2 | 4 | 114.81 | 326.71 | 0 | 0 | 41 | 4 | 22.31 | 0.00% | 60.00% | 0.63 | 0.81 | 116.28 | 3 | 1.37 |
| 326 | 2010 | 299 | 44.52 | 4 | 236.49 | 1586.8 | 0 | 0 | 47 | 4 | 17.21 | 0.00% | 56.00% | 0.4 | 0.43 | 115.39 | 6 | 1.46 |
| 327 | 2010 | 593 | 40.4 | 4 | 207.44 | 504.95 | 0 | 0 | 37 | 2 | 13.73 | 0.00% | 100.00% | 0.98 | 0.48 | 109.70 | 3 | 1.06 |
| 328 | 2010 | 238 | 43.67 | 4 | 235.34 | 1547.02 | 0 | 0 | 30 | 3 | 8.4 | 0.00% | 70.00% | 0.24 | 0.29 | 107.80 | 3 | 0.78 |
| 329 | 2010 | 256 | 39.68 | 3 | 207.46 | 1591.28 | 0 | 0 | 43 | 4 | 17.21 | 0.00% | 96.00% | 0.86 | 0.44 | 119.74 | 7 | 1.36 |
| 330 | 2010 | 565 | 43.09 | 4 | 219.45 | 515.68 | 0 | 0 | 19 | 3 | 2.24 | 19.00% | 63.00% | 0.44 | 0.44 | 109.29 | 4 | 0.98 |
| 331 | 2010 | 519 | 43.6 | 4 | 209.42 | 505.74 | 0 | 0 | 44 | 2 | 13.71 | 0.00% | 100.00% | 1.08 | 0.47 | 109.76 | 3 | 0.67 |
| 332 | 2010 | 498 | 22.84 | 5 | 110.52 | 1246.55 | 0 | 0 | 46 | 3 | 18.31 | 0.00% | 54.00% | 0.91 | 0.47 | 111.80 | 4 | 0.95 |
| 333 | 2010 | 362 | 41.2 | 5 | 214.08 | 502.91 | 0 | 0 | 20 | 2 | 13.74 | 0.00% | 100.00% | 1.05 | 0.42 | 109.15 | 2 | 0.6 |
| 334 | 2010 | 470 | 45.94 | 4 | 235.01 | 178.46 | 0 | 0 | 47 | 2 | 5.51 | 7.00% | 73.00% | 0.46 | 0.4 | 109.02 | 3 | 1.19 |
| 335 | 2010 | 262 | 20.22 | 3 | 119.77 | 1232.84 | 0 | 0 | 44 | 3 | 18.35 | 0.00% | 65.00% | 0.98 | 0.3 | 113.83 | 3 | 1.25 |
| 336 | 2010 | 679 | 23.47 | 4 | 119.31 | 515.18 | 0 | 0 | 42 | 3 | 1.82 | 11.00% | 76.00% | 1.05 | 0.99 | 109.83 | 3 | 0.91 |
| 337 | 2010 | 246 | 43.05 | 4 | 212.21 | 430.38 | 0 | 0 | 34 | 5 | 6.73 | 19.00% | 100.00% | 0.49 | 0.31 | 117.50 | 2 | 1.27 |
| 338 | 2010 | 333 | 23.02 | 5 | 123.16 | 4778.84 | 0 | 0 | 45 | 3 | 5.63 | 0.00% | 53.00% | 0.43 | 0.36 | 116.89 | 2 | 1.5 |
| 339 | 2010 | 289 | 21.9 | 4 | 113.81 | 334.39 | 0 | 0 | 47 | 4 | 17.2 | 0.00% | 50.00% | 0.73 | 0.73 | 117.63 | 2 | 0.95 |
| 340 | 2010 | 542 | 20.38 | 5 | 126.47 | 362.97 | 0 | 0 | 43 | 4 | 22.29 | 0.00% | 78.00% | 0.69 | 0.72 | 112.07 | 2 | 1.11 |
| 341 | 2010 | 402 | 23.77 | 3 | 112.41 | 1588.43 | 0 | 0 | 48 | 4 | 17.16 | 0.00% | 93.00% | 0.84 | 0.47 | 114.72 | 6 | 1.46 |
| 342 | 2010 | 582 | 41.75 | 4 | 206.7 | 941.75 | 0 | 0 | 52 | 5 | 9.04 | 0.00% | 42.00% | 1.09 | 0.84 | 113.36 | 4 | 0.89 |
| 343 | 2010 | 318 | 42.69 | 3 | 211.6 | 665.22 | 0 | 1 | 28 | 2 | 12.86 | 0.00% | 100.00% | 0.95 | 0.38 | 116.75 | 4 | 0.81 |
| 344 | 2010 | 534 | 40.14 | 5 | 216.54 | 335.37 | 0 | 1 | 48 | 2 | 9.1 | 24.00% | 100.00% | 0.26 | 0.27 | 115.87 | 2 | 1.04 |
| 345 | 2010 | 671 | 32.2 | 4 | 166.49 | 349.19 | 0 | 0 | 69 | 4 | 22.27 | 0.00% | 66.00% | 0.68 | 0.66 | 119.46 | 3 | 1.7 |
| 346 | 2010 | 436 | 33.69 | 5 | 157.57 | 4744.64 | 0 | 1 | 51 | 3 | 5.53 | 0.00% | 77.00% | 0.49 | 0.37 | 112.88 | 3 | 1.7 |
| 347 | 2010 | 531 | 29.92 | 4 | 175.65 | 452.03 | 0 | 1 | 68 | 4 | 2.46 | 0.00% | 86.00% | 0.96 | 0.32 | 112.27 | 4 | 0.73 |
| 348 | 2010 | 782 | 33.76 | 4 | 171.84 | 498.05 | 0 | 1 | 43 | 2 | 10.52 | 0.00% | 50.00% | 0.98 | 0.46 | 117.36 | 2 | 1.56 |
| 349 | 2010 | 268 | 32.28 | 5 | 161.03 | 513.57 | 0 | 1 | 28 | 2 | 13.79 | 0.00% | 50.00% | 0.47 | 0.41 | 108.68 | 2 | 0.66 |
| 350 | 2010 | 341 | 33.36 | 4 | 175.69 | 494.27 | 0 | 1 | 25 | 2 | 10.62 | 0.00% | 47.00% | 0.91 | 0.38 | 112.54 | 4 | 0.87 |
| 351 | 2010 | 405 | 31.19 | 3 | 168.04 | 481.52 | 0 | 1 | 53 | 3 | 1.7 | 17.00% | 62.00% | 0.47 | 0.3 | 117.63 | 4 | 1.25 |
| 352 | 2010 | 777 | 33.14 | 5 | 166.66 | 528.54 | 0 | 0 | 40 | 3 | 15.82 | 0.00% | 76.00% | 1.51 | 0.63 | 112.95 | 4 | 1.13 |
| 353 | 2010 | 856 | 37.58 | 5 | 190.21 | 522.6 | 0 | 0 | 41 | 2 | 13.68 | 0.00% | 51.00% | 0.42 | 0.42 | 112.88 | 3 | 1.12 |
| 354 | 2010 | 576 | 37.64 | 5 | 203.03 | 153.26 | 0 | 0 | 44 | 2 | 5.44 | 3.00% | 100.00% | 0.41 | 0.35 | 115.87 | 2 | 1.68 |
| 355 | 2010 | 493 | 36.62 | 7 | 189.82 | 799.68 | 0 | 1 | 49 | 2 | 19.83 | 0.00% | 76.00% | 0.98 | 0.48 | 116.00 | 2 | 1.12 |
| 356 | 2010 | 511 | 34.64 | 6 | 202.94 | 358 | 0 | 0 | 37 | 2 | 9.14 | 18.00% | 100.00% | 0.98 | 0.29 | 119.46 | 1 | 1.46 |
| 357 | 2010 | 915 | 35.72 | 6 | 193.58 | 1625.55 | 0 | 1 | 47 | 3 | 10.95 | 0.00% | 69.00% | 0.97 | 1.14 | 115.19 | 2 | 0.93 |
| 358 | 2010 | 442 | 35.58 | 6 | 187.38 | 560.7 | 0 | 0 | 48 | 3 | 20.54 | 0.00% | 76.00% | 1.44 | 0.47 | 114.04 | 4 | 1.79 |
| 359 | 2010 | 951 | 35.7 | 7 | 201.85 | 460.69 | 0 | 0 | 48 | 4 | 4.33 | 0.00% | 50.00% | 1.05 | 0.35 | 116.14 | 2 | 1.65 |
| 360 | 2010 | 771 | 34.96 | 5 | 196.08 | 1617.8 | 0 | 0 | 39 | 3 | 11 | 0.00% | 64.00% | 0.93 | 0.82 | 109.83 | 3 | 1.4 |
| 361 | 2010 | 509 | 35.04 | 3 | 199.56 | 440.65 | 0 | 0 | 37 | 5 | 6.69 | 20.00% | 100.00% | 0.42 | 0.29 | 110.85 | 2 | 1.88 |
| 362 | 2010 | 749 | 37.43 | 2 | 199.89 | 362.12 | 0 | 0 | 46 | 2 | 9.11 | 22.00% | 100.00% | 0.92 | 0.34 | 110.98 | 2 | 2.11 |
| 363 | 2010 | 717 | 35.36 | 4 | 185.83 | 408.29 | 0 | 1 | 54 | 5 | 5.16 | 14.00% | 72.00% | 0.78 | 0.72 | 117.36 | 1 | 1.07 |
| 364 | 2010 | 967 | 37.04 | 3 | 183.35 | 523.77 | 0 | 0 | 42 | 3 | 2.3 | 18.00% | 69.00% | 0.94 | 0.84 | 113.90 | 3 | 1.79 |
| 365 | 2010 | 745 | 35.66 | 3 | 191.69 | 494.66 | 0 | 1 | 51 | 4 | 5.74 | 0.00% | 63.00% | 1.03 | 0.38 | 111.26 | 4 | 1.58 |
| 366 | 2010 | 833 | 35.76 | 4 | 200.98 | 459.3 | 0 | 1 | 53 | 4 | 2.47 | 0.00% | 71.00% | 0.86 | 0.3 | 110.98 | 5 | 0.88 |
| 367 | 2010 | 539 | 35.73 | 3 | 195.1 | 492.03 | 0 | 1 | 51 | 4 | 5.63 | 0.00% | 54.00% | 0.99 | 0.4 | 116.68 | 2 | 1.35 |
| 368 | 2010 | 461 | 35.84 | 3 | 184.53 | 815.87 | 0 | 0 | 44 | 2 | 19.82 | 0.00% | 78.00% | 0.76 | 0.61 | 109.42 | 1 | 1.33 |
| 369 | 2010 | 601 | 37.28 | 4 | 196.58 | 473.03 | 0 | 0 | 44 | 4 | 5.64 | 0.00% | 76.00% | 0.48 | 0.33 | 114.92 | 4 | 2.04 |
| 370 | 2010 | 553 | 36.84 | 4 | 184.55 | 341.38 | 0 | 0 | 41 | 2 | 9.12 | 24.00% | 100.00% | 0.26 | 0.28 | 119.53 | 2 | 1.84 |
| 371 | 2010 | 706 | 34.45 | 3 | 194.71 | 358.87 | 0 | 0 | 43 | 2 | 9.07 | 24.00% | 52.00% | 0.59 | 0.6 | 115.19 | 2 | 2.25 |
| 372 | 2010 | 942 | 37.32 | 3 | 199.41 | 1526.28 | 0 | 0 | 43 | 3 | 8.48 | 0.00% | 59.00% | 0.24 | 0.22 | 110.31 | 3 | 1.48 |
| 373 | 2010 | 610 | 845.76 | 6 | 4142.98 | 862.17 | 0 | 0 | 50 | 2 | 25.81 | 0.00% | 73.00% | 0.93 | 0.42 | 116.41 | 2 | 1.94 |
| 374 | 2010 | 854 | 48.03 | 7 | 246.18 | 504.54 | 0 | 0 | 48 | 2 | 10.53 | 0.00% | 39.00% | 0.43 | 0.43 | 116.75 | 4 | 0.83 |
| 375 | 2010 | 649 | 46.59 | 6 | 245.65 | 526.26 | 0 | 0 | 43 | 3 | 15.76 | 0.00% | 79.00% | 1.51 | 0.69 | 113.16 | 3 | 1.02 |
| 376 | 2010 | 425 | 46.52 | 6 | 253.55 | 489.07 | 0 | 0 | 41 | 3 | 1.68 | 9.00% | 79.00% | 0.93 | 0.43 | 113.90 | 2 | 2.18 |
| 377 | 2010 | 1090 | 47.01 | 6 | 246.92 | 4771.25 | 0 | 0 | 50 | 3 | 5.48 | 0.00% | 69.00% | 0.43 | 0.3 | 119.53 | 2 | 2.21 |
| 378 | 2010 | 968 | 48.42 | 7 | 250.31 | 507.54 | 0 | 1 | 53 | 3 | 1.77 | 16.00% | 68.00% | 1.04 | 0.36 | 110.31 | 3 | 1.39 |
| 379 | 2010 | 473 | 44.66 | 7 | 248.87 | 536.92 | 0 | 1 | 54 | 3 | 15.77 | 0.00% | 85.00% | 1.51 | 0.66 | 115.73 | 3 | 1.97 |
| 380 | 2010 | 471 | 47.92 | 6 | 254.47 | 910.83 | 0 | 0 | 36 | 5 | 6.99 | 0.00% | 52.00% | 1.01 | 0.89 | 112.61 | 6 | 2.02 |
| 381 | 2010 | 746 | 47.77 | 7 | 238.84 | 152.41 | 0 | 0 | 44 | 2 | 5.54 | 3.00% | 100.00% | 0.43 | 0.36 | 106.91 | 3 | 2.19 |
| 382 | 2010 | 431 | 20.72 | 4 | 129.71 | 503.34 | 0 | 0 | 33 | 3 | 2.31 | 17.00% | 64.00% | 0.91 | 0.93 | 109.83 | 4 | 2.43 |
| 383 | 2010 | 465 | 21.82 | 4 | 120.03 | 518.94 | 0 | 0 | 52 | 3 | 2.32 | 20.00% | 56.00% | 0.98 | 1.07 | 109.02 | 3 | 1.53 |
| 384 | 2010 | 425 | 22.4 | 4 | 119.44 | 430.79 | 0 | 0 | 32 | 5 | 6.75 | 17.00% | 75.00% | 0.81 | 0.8 | 111.59 | 2 | 2.68 |
| 385 | 2010 | 694 | 21.54 | 5 | 131.5 | 486.41 | 0 | 0 | 47 | 4 | 5.75 | 0.00% | 78.00% | 0.47 | 0.43 | 105.90 | 4 | 1.45 |
| 386 | 2010 | 1091 | 20.78 | 4 | 119.05 | 809.36 | 0 | 0 | 37 | 2 | 19.78 | 0.00% | 81.00% | 1.1 | 0.46 | 115.05 | 5 | 2.64 |
| 387 | 2010 | 998 | 22.6 | 4 | 130.93 | 537.14 | 0 | 0 | 35 | 2 | 13.8 | 0.00% | 52.00% | 0.49 | 0.48 | 112.27 | 4 | 1.56 |
| 388 | 2010 | 783 | 23.51 | 3 | 125.3 | 678.84 | 0 | 0 | 53 | 2 | 12.86 | 0.00% | 66.00% | 0.49 | 0.4 | 119.46 | 4 | 2.26 |
| 389 | 2010 | 1157 | 20.35 | 4 | 129.82 | 1631.99 | 0 | 0 | 52 | 3 | 8.01 | 0.00% | 40.00% | 0.48 | 0.44 | 109.76 | 4 | 1.82 |
| 390 | 2010 | 881 | 57.28 | 8 | 317.73 | 508.11 | 0 | 0 | 49 | 3 | 2.29 | 16.00% | 84.00% | 0.98 | 0.36 | 110.10 | 2 | 2.23 |
| 391 | 2010 | 1225 | 58.28 | 8 | 317.75 | 481.95 | 0 | 0 | 51 | 4 | 5.73 | 0.00% | 63.00% | 1.06 | 0.37 | 112.07 | 3 | 1.64 |
| 392 | 2010 | 924 | 58.28 | 10 | 304.12 | 475.64 | 0 | 1 | 49 | 4 | 5.68 | 0.00% | 49.00% | 0.51 | 0.49 | 113.09 | 4 | 1.2 |
| 393 | 2010 | 870 | 58.39 | 9 | 311.78 | 474.9 | 0 | 0 | 33 | 4 | 3.22 | 0.00% | 68.00% | 0.94 | 0.29 | 109.56 | 3 | 1.28 |
| 394 | 2010 | 885 | 57.58 | 10 | 316.94 | 422.24 | 0 | 1 | 51 | 5 | 6.71 | 16.00% | 79.00% | 1.09 | 0.48 | 114.17 | 3 | 2.31 |
| 395 | 2010 | 813 | 59.99 | 9 | 299.42 | 1616.98 | 0 | 0 | 56 | 3 | 11.01 | 0.00% | 79.00% | 0.6 | 0.73 | 116.21 | 3 | 1.71 |
| 396 | 2010 | 1059 | 60.47 | 9 | 305.23 | 344.47 | 0 | 0 | 44 | 2 | 9.16 | 25.00% | 55.00% | 0.52 | 0.63 | 115.80 | 1 | 1.71 |
| 397 | 2010 | 915 | 58.65 | 10 | 311.63 | 492.96 | 0 | 0 | 49 | 4 | 5.7 | 0.00% | 86.00% | 0.42 | 0.35 | 116.14 | 4 | 1.84 |
| 398 | 2010 | 1170 | 150.3 | 9 | 762.42 | 486.98 | 0 | 0 | 37 | 4 | 5.74 | 0.00% | 50.00% | 0.52 | 0.47 | 111.93 | 4 | 1.09 |
| 399 | 2010 | 1022 | 152.14 | 8 | 774.17 | 478.64 | 0 | 0 | 30 | 3 | 5.18 | 1.00% | 83.00% | 0.97 | 1.19 | 116.61 | 3 | 2.51 |
| 400 | 2010 | 747 | 153.11 | 9 | 754.11 | 1704.41 | 0 | 0 | 37 | 3 | 10.53 | 0.00% | 53.00% | 0.49 | 0.6 | 113.43 | 3 | 1.61 |
| 401 | 2010 | 1007 | 150.88 | 8 | 763.08 | 476.05 | 0 | 0 | 30 | 4 | 3.14 | 0.00% | 61.00% | 0.99 | 0.47 | 107.25 | 4 | 1.15 |
| 402 | 2010 | 637 | 153.68 | 8 | 768.73 | 878.12 | 0 | 0 | 30 | 2 | 25.81 | 0.00% | 82.00% | 0.73 | 0.83 | 117.50 | 3 | 1.48 |
| 403 | 2010 | 857 | 151.54 | 8 | 763.44 | 343.88 | 0 | 0 | 32 | 2 | 9.07 | 28.00% | 50.00% | 0.51 | 0.6 | 119.67 | 2 | 1.71 |
| 404 | 2010 | 1102 | 153.36 | 8 | 754.74 | 1520.75 | 0 | 0 | 35 | 2 | 13.39 | 0.00% | 68.00% | 0.94 | 0.37 | 111.87 | 3 | 3.02 |
| 405 | 2010 | 1254 | 153.6 | 8 | 766.43 | 1529.28 | 0 | 0 | 29 | 2 | 13.41 | 0.00% | 79.00% | 0.23 | 0.27 | 113.09 | 2 | 1.58 |
| 406 | 2010 | 512 | 153.27 | 8 | 767.88 | 417.72 | 0 | 0 | 51 | 5 | 6.76 | 18.00% | 84.00% | 1.07 | 0.38 | 117.50 | 1 | 2.19 |
| 407 | 2010 | 508 | 153.61 | 8 | 767.93 | 815.15 | 0 | 0 | 33 | 2 | 19.79 | 0.00% | 67.00% | 0.77 | 0.72 | 109.97 | 2 | 1.86 |
| 408 | 2010 | 634 | 150.93 | 8 | 773.8 | 496.42 | 0 | 0 | 51 | 4 | 5.77 | 0.00% | 81.00% | 0.46 | 0.41 | 114.51 | 3 | 2.07 |
| 409 | 2010 | 546 | 153.05 | 8 | 754.5 | 484.5 | 0 | 0 | 28 | 4 | 3.24 | 0.00% | 57.00% | 0.88 | 0.46 | 116.34 | 3 | 1.28 |
| 410 | 2010 | 950 | 43.99 | 10 | 243.98 | 479.67 | 0 | 0 | 35 | 4 | 5.71 | 0.00% | 50.00% | 0.98 | 0.48 | 110.31 | 3 | 1.3 |
| 411 | 2010 | 1389 | 45.44 | 9 | 250.44 | 1709.42 | 0 | 1 | 49 | 3 | 10.56 | 0.00% | 45.00% | 0.46 | 0.48 | 109.08 | 3 | 2.39 |
| 412 | 2010 | 1258 | 46.22 | 10 | 244.46 | 859.56 | 0 | 0 | 30 | 2 | 25.76 | 0.00% | 83.00% | 0.98 | 0.38 | 111.19 | 2 | 2.24 |
| 413 | 2010 | 998 | 42.3 | 10 | 229.9 | 452.34 | 0 | 0 | 36 | 3 | 5.2 | 1.00% | 65.00% | 0.99 | 1.2 | 107.66 | 4 | 1.31 |
| 414 | 2010 | 1506 | 44.78 | 10 | 232.79 | 418.3 | 0 | 0 | 48 | 5 | 6.76 | 19.00% | 92.00% | 0.76 | 0.81 | 113.49 | 2 | 2.66 |
| 415 | 2010 | 1467 | 45.56 | 10 | 231.75 | 508.02 | 0 | 0 | 30 | 4 | 3.2 | 0.00% | 49.00% | 0.81 | 0.57 | 117.56 | 4 | 2.77 |
| 416 | 2010 | 761 | 44.51 | 9 | 231.42 | 493.16 | 0 | 0 | 51 | 4 | 3.16 | 0.00% | 58.00% | 0.85 | 0.27 | 115.73 | 3 | 1.12 |
| 417 | 2010 | 917 | 46.33 | 10 | 240.45 | 351.71 | 0 | 0 | 30 | 2 | 11.85 | 24.00% | 66.00% | 0.24 | 0.3 | 117.23 | 1 | 2.37 |
| 418 | 2010 | 1425 | 52.5 | 7 | 282.19 | 1507.55 | 0 | 1 | 42 | 2 | 13.3 | 0.00% | 70.00% | 0.96 | 0.33 | 116.28 | 3 | 1.86 |
| 419 | 2010 | 1198 | 54.93 | 7 | 292.96 | 1597.39 | 0 | 0 | 31 | 3 | 10.99 | 0.00% | 87.00% | 0.59 | 0.55 | 106.37 | 3 | 1.76 |
| 420 | 2010 | 1092 | 55.44 | 7 | 289.39 | 367.03 | 0 | 0 | 28 | 2 | 9.13 | 20.00% | 100.00% | 0.25 | 0.22 | 113.49 | 1 | 3.53 |
| 421 | 2010 | 975 | 54 | 7 | 281.01 | 468.18 | 0 | 1 | 44 | 3 | 5.2 | 7.00% | 81.00% | 0.45 | 0.36 | 109.29 | 3 | 2.16 |
| 422 | 2010 | 996 | 53.26 | 7 | 286.72 | 476.55 | 0 | 1 | 45 | 3 | 5.12 | 4.00% | 70.00% | 0.48 | 0.3 | 110.98 | 4 | 1.77 |
| 423 | 2010 | 1265 | 55.42 | 7 | 282.76 | 1158.07 | 0 | 1 | 45 | 3 | 54.49 | 0.00% | 52.00% | 1.1 | 0.48 | 112.14 | 3 | 1.1 |
| 424 | 2010 | 1468 | 52.19 | 8 | 277.92 | 448.59 | 0 | 0 | 28 | 3 | 5.18 | 0.00% | 79.00% | 0.25 | 0.29 | 112.21 | 2 | 1.11 |
| 425 | 2010 | 1119 | 54.6 | 8 | 289 | 802.59 | 0 | 0 | 30 | 2 | 19.89 | 0.00% | 67.00% | 0.93 | 0.34 | 112.95 | 1 | 3.09 |
| 426 | 2010 | 675 | 53.07 | 8 | 286.76 | 388.45 | 0 | 0 | 28 | 2 | 11.79 | 33.00% | 46.00% | 0.51 | 0.57 | 116.89 | 2 | 1.84 |
| 427 | 2010 | 1113 | 52.71 | 8 | 278.58 | 436.84 | 0 | 0 | 29 | 5 | 6.74 | 13.00% | 90.00% | 1.01 | 0.37 | 119.74 | 2 | 1.69 |
| 428 | 2010 | 1325 | 53.74 | 6 | 287.63 | 1687.4 | 0 | 1 | 45 | 3 | 10.52 | 0.00% | 65.00% | 0.49 | 0.58 | 117.29 | 4 | 1.68 |
| 429 | 2010 | 600 | 54.61 | 7 | 290.63 | 1604.4 | 0 | 0 | 27 | 3 | 10.96 | 0.00% | 78.00% | 0.27 | 0.22 | 108.07 | 3 | 1.98 |
| 430 | 2010 | 1323 | 45.61 | 2 | 253.92 | 1551.5 | 0 | 0 | 53 | 3 | 8.36 | 0.00% | 76.00% | 0.99 | 1.22 | 108.34 | 2 | 2.83 |
| 431 | 2010 | 1011 | 44.65 | 2 | 239.3 | 363.75 | 0 | 0 | 45 | 2 | 9.16 | 19.00% | 100.00% | 0.97 | 0.33 | 114.58 | 2 | 3.92 |
| 432 | 2010 | 1384 | 46.51 | 4 | 249.11 | 812.64 | 0 | 0 | 44 | 2 | 19.79 | 0.00% | 68.00% | 0.63 | 0.61 | 109.15 | 2 | 3.44 |
| 433 | 2010 | 1857 | 44.61 | 4 | 239.6 | 969.56 | 0 | 1 | 54 | 5 | 9.01 | 0.00% | 41.00% | 1.01 | 1.16 | 110.78 | 6 | 3.92 |
| 434 | 2010 | 1159 | 43.68 | 3 | 241 | 1623.4 | 0 | 0 | 45 | 3 | 8.13 | 0.00% | 42.00% | 0.52 | 0.52 | 116.07 | 3 | 2.7 |
| 435 | 2010 | 1770 | 43.58 | 4 | 246.55 | 530.46 | 0 | 0 | 51 | 3 | 2.3 | 16.00% | 85.00% | 0.42 | 0.31 | 111.32 | 3 | 4.06 |
| 436 | 2010 | 752 | 43.76 | 2 | 242.15 | 396.11 | 0 | 0 | 44 | 5 | 5.17 | 17.00% | 73.00% | 0.7 | 0.81 | 115.53 | 3 | 4.39 |
| 437 | 2010 | 788 | 42.48 | 3 | 243.04 | 490.96 | 0 | 0 | 45 | 4 | 2.43 | 0.00% | 59.00% | 0.93 | 0.55 | 108.20 | 3 | 1.88 |
| 438 | 2010 | 1740 | 46.46 | 4 | 254.14 | 508.15 | 0 | 0 | 49 | 3 | 2.29 | 20.00% | 98.00% | 0.94 | 0.4 | 116.55 | 3 | 2.84 |
| 439 | 2010 | 837 | 42.92 | 3 | 258.84 | 454.69 | 0 | 0 | 37 | 5 | 6.71 | 10.00% | 83.00% | 0.69 | 0.8 | 116.55 | 3 | 4.74 |
| 440 | 2010 | 1899 | 44.91 | 4 | 257.19 | 676.49 | 0 | 1 | 38 | 2 | 12.82 | 0.00% | 100.00% | 0.93 | 0.33 | 115.80 | 2 | 4.34 |
| 441 | 2010 | 724 | 43.82 | 3 | 251.51 | 561.01 | 0 | 0 | 36 | 3 | 20.45 | 0.00% | 82.00% | 1.36 | 0.33 | 119.26 | 2 | 2.52 |
| 442 | 2010 | 858 | 38.4 | 4 | 229.96 | 448.18 | 0 | 0 | 32 | 4 | 4.46 | 0.00% | 48.00% | 0.42 | 0.36 | 109.70 | 3 | 3.46 |
| 443 | 2010 | 1815 | 38.25 | 3 | 220.37 | 1634.65 | 0 | 0 | 30 | 3 | 8.04 | 0.00% | 36.00% | 0.54 | 0.58 | 113.16 | 4 | 1.68 |
| 444 | 2010 | 2098 | 37.12 | 4 | 213.1 | 834.82 | 0 | 0 | 38 | 2 | 19.81 | 0.00% | 69.00% | 0.99 | 0.35 | 116.07 | 3 | 4.34 |
| 445 | 2010 | 1500 | 40.76 | 4 | 215.56 | 525.43 | 0 | 0 | 39 | 3 | 2.33 | 13.00% | 92.00% | 0.44 | 0.32 | 113.56 | 3 | 4.19 |
| 446 | 2010 | 985 | 39.46 | 5 | 220.15 | 463.72 | 0 | 0 | 34 | 4 | 4.35 | 0.00% | 46.00% | 0.45 | 0.32 | 117.29 | 2 | 2.21 |
| 447 | 2010 | 1477 | 39.91 | 3 | 215.19 | 700.75 | 0 | 0 | 37 | 2 | 12.9 | 0.00% | 100.00% | 1.04 | 0.42 | 111.73 | 3 | 4.54 |
| 448 | 2010 | 1145 | 39.44 | 4 | 230.01 | 829.75 | 0 | 0 | 31 | 2 | 19.9 | 0.00% | 69.00% | 0.99 | 0.33 | 119.74 | 3 | 2.24 |
| 449 | 2010 | 2096 | 37.32 | 3 | 210.75 | 701.19 | 0 | 0 | 38 | 2 | 12.82 | 0.00% | 71.00% | 0.41 | 0.41 | 111.80 | 2 | 4.73 |
| 1 | 2017 | 9 | 19.64 | 2 | 120.44 | 556.02 | 0.2 | 1 | 39 | 1 | 6.7 | 0.00% | 74.00% | 1.09 | 0.88 | 109.63 | 4 | 0.02 |
| 2 | 2017 | 12 | 22 | 2 | 99.49 | 576.2 | 0.2 | 1 | 41 | 2 | 6.72 | 0.00% | 72.00% | 0.93 | 0.73 | 115.67 | 5 | 0.04 |
| 3 | 2017 | 9 | 19.64 | 2 | 118.23 | 559.77 | 0.17 | 1 | 38 | 1 | 6.76 | 0.00% | 64.00% | 1.04 | 0.48 | 115.19 | 2 | 0.07 |
| 4 | 2017 | 8 | 19.02 | 2 | 112.35 | 561.01 | 0.18 | 1 | 42 | 3 | 6.77 | 0.00% | 59.00% | 1.06 | 0.38 | 116.07 | 2 | 0.04 |
| 5 | 2017 | 12 | 19.64 | 3 | 109.37 | 786.08 | 0.21 | 0 | 32 | 3 | 2.14 | 21.00% | 70.00% | 0.43 | 0.43 | 117.70 | 4 | 0.06 |
| 6 | 2017 | 16 | 27.87 | 3 | 150.84 | 691.92 | 0.19 | 0 | 52 | 1 | 17.39 | 0.00% | 70.00% | 0.24 | 0.24 | 111.12 | 2 | 0.05 |
| 7 | 2017 | 17 | 27.24 | 4 | 150.34 | 664.83 | 0.2 | 0 | 38 | 2 | 9.83 | 0.00% | 72.00% | 0.8 | 0.73 | 114.51 | 6 | 0.03 |
| 8 | 2017 | 24 | 21.78 | 2 | 115.98 | 785.68 | 0.21 | 0 | 34 | 3 | 2.09 | 21.00% | 62.00% | 0.42 | 0.3 | 119.67 | 3 | 0.04 |
| 9 | 2017 | 16 | 26.37 | 3 | 146.88 | 636.1 | 0.17 | 0 | 31 | 2 | 16.15 | 0.00% | 59.00% | 1.08 | 0.93 | 115.94 | 2 | 0.05 |
| 10 | 2017 | 13 | 24.46 | 4 | 141.12 | 635.47 | 0.18 | 0 | 30 | 2 | 16.94 | 0.00% | 43.00% | 0.76 | 0.79 | 120.35 | 2 | 0.06 |
| 11 | 2017 | 16 | 20.51 | 2 | 103.67 | 983.23 | 0.19 | 1 | 51 | 2 | 23.45 | 0.00% | 70.00% | 1.51 | 0.33 | 113.70 | 3 | 0.06 |
| 12 | 2017 | 17 | 21.32 | 2 | 102.47 | 631.45 | 0.18 | 0 | 33 | 3 | 1.7 | 0.00% | 60.00% | 0.26 | 0.21 | 114.24 | 3 | 0.02 |
| 13 | 2017 | 23 | 24.64 | 3 | 141.23 | 1023.46 | 0.18 | 0 | 59 | 3 | 31.93 | 0.00% | 100.00% | 0.24 | 0.23 | 116.14 | 3 | 0.04 |
| 14 | 2017 | 12 | 27.39 | 4 | 143.47 | 209.51 | 0.19 | 0 | 51 | 5 | 3.49 | 8.00% | 40.00% | 0.47 | 0.4 | 113.97 | 4 | 0.06 |
| 15 | 2017 | 25 | 25.89 | 3 | 153.74 | 636.76 | 0.15 | 0 | 30 | 2 | 16.83 | 0.00% | 53.00% | 0.63 | 0.68 | 111.87 | 3 | 0.04 |
| 16 | 2017 | 11 | 28.08 | 3 | 154.41 | 203.59 | 0.18 | 0 | 58 | 2 | 3.66 | 50.00% | 62.00% | 0.41 | 0.37 | 110.58 | 3 | 0.04 |
| 17 | 2017 | 23 | 27.18 | 3 | 147.1 | 1401.2 | 0.19 | 0 | 32 | 3 | 23.16 | 0.00% | 55.00% | 1.06 | 0.34 | 119.67 | 3 | 0.09 |
| 18 | 2017 | 16 | 27.71 | 4 | 147.58 | 198.72 | 0.16 | 1 | 50 | 3 | 3.46 | 4.00% | 40.00% | 0.47 | 0.41 | 109.42 | 2 | 0.06 |
| 19 | 2017 | 26 | 28.12 | 3 | 137.72 | 621.63 | 0.18 | 0 | 32 | 3 | 16.06 | 0.00% | 51.00% | 1.01 | 0.95 | 106.98 | 2 | 0.01 |
| 20 | 2017 | 28 | 26.73 | 2 | 152.76 | 757.68 | 0.22 | 0 | 75 | 3 | 12.92 | 0.00% | 83.00% | 0.63 | 0.6 | 116.95 | 2 | 0.04 |
| 21 | 2017 | 14 | 24.6 | 3 | 148.71 | 616.19 | 0.16 | 0 | 31 | 2 | 16.12 | 0.00% | 41.00% | 0.93 | 0.95 | 109.90 | 2 | 0.05 |
| 22 | 2017 | 17 | 26.06 | 2 | 141.44 | 186.77 | 0.2 | 1 | 58 | 3 | 3.65 | 48.00% | 74.00% | 0.48 | 0.3 | 111.12 | 4 | 0.04 |
| 23 | 2017 | 14 | 27.11 | 4 | 137.22 | 179.89 | 0.2 | 1 | 53 | 2 | 3.37 | 4.00% | 63.00% | 0.48 | 0.46 | 109.08 | 3 | 0.03 |
| 24 | 2017 | 21 | 26.91 | 3 | 135.35 | 617.39 | 0.19 | 0 | 32 | 2 | 16.93 | 0.00% | 45.00% | 0.71 | 0.72 | 113.16 | 3 | 0.08 |
| 25 | 2017 | 28 | 25.34 | 3 | 133.76 | 645.07 | 0.18 | 0 | 39 | 3 | 9.9 | 0.00% | 81.00% | 0.74 | 0.72 | 119.19 | 1 | 0.06 |
| 26 | 2017 | 25 | 25.91 | 3 | 149.3 | 1032.82 | 0.19 | 0 | 58 | 2 | 31.95 | 0.00% | 56.00% | 0.91 | 0.87 | 109.22 | 3 | 0.05 |
| 27 | 2017 | 22 | 27.83 | 4 | 141.66 | 774.65 | 0.18 | 0 | 57 | 3 | 7.83 | 0.00% | 36.00% | 0.92 | 0.96 | 114.24 | 2 | 0.06 |
| 28 | 2017 | 16 | 24.93 | 2 | 140.99 | 753.98 | 0.2 | 0 | 74 | 3 | 13.03 | 0.00% | 56.00% | 0.94 | 0.98 | 111.80 | 4 | 0.03 |
| 29 | 2017 | 31 | 26.35 | 3 | 141.25 | 1382.71 | 0.18 | 0 | 34 | 3 | 23.15 | 0.00% | 78.00% | 1 | 0.4 | 112.68 | 2 | 0.06 |
| 30 | 2017 | 23 | 32.39 | 5 | 273.55 | 732.98 | 0.17 | 0 | 52 | 3 | 9 | 0.00% | 44.00% | 0.53 | 0.48 | 110.65 | 3 | 0.07 |
| 31 | 2017 | 18 | 27.27 | 3 | 139.4 | 763.4 | 0.2 | 0 | 76 | 2 | 12.95 | 0.00% | 59.00% | 0.98 | 0.68 | 120.48 | 5 | 0.09 |
| 32 | 2017 | 15 | 25.91 | 3 | 143.92 | 1014.74 | 0.2 | 1 | 58 | 4 | 31.81 | 0.00% | 61.00% | 0.95 | 0.88 | 109.97 | 6 | 0.14 |
| 33 | 2017 | 39 | 27.02 | 4 | 137.66 | 1369.39 | 0.2 | 0 | 33 | 5 | 23.16 | 0.00% | 71.00% | 0.92 | 0.36 | 115.67 | 2 | 0.12 |
| 34 | 2017 | 26 | 48.19 | 7 | 256.82 | 767.64 | 0.17 | 0 | 50 | 4 | 9.1 | 0.00% | 43.00% | 0.6 | 0.51 | 117.63 | 6 | 0.06 |
| 35 | 2017 | 37 | 29.87 | 7 | 263.35 | 555.42 | 0.17 | 0 | 42 | 2 | 6.68 | 0.00% | 65.00% | 1.03 | 0.96 | 110.17 | 3 | 0.05 |
| 36 | 2017 | 29 | 21.03 | 2 | 112.44 | 1617.48 | 0.21 | 1 | 44 | 3 | 17.35 | 0.00% | 80.00% | 0.45 | 0.47 | 117.43 | 5 | 0.07 |
| 37 | 2017 | 21 | 32.09 | 5 | 263.89 | 957.18 | 0.16 | 0 | 52 | 2 | 23.47 | 0.00% | 72.00% | 1.47 | 0.45 | 106.57 | 2 | 0.04 |
| 38 | 2017 | 25 | 6.15 | 4 | 31.62 | 710.35 | 0.21 | 1 | 69 | 4 | 10.05 | 0.00% | 50.00% | 0.96 | 0.91 | 111.32 | 3 | 0.05 |
| 39 | 2017 | 25 | 19.15 | 3 | 92.4 | 374.85 | 0.2 | 1 | 43 | 5 | 11.85 | 23.00% | 51.00% | 0.92 | 0.25 | 113.90 | 5 | 0.05 |
| 40 | 2017 | 28 | 49.41 | 5 | 263.15 | 951.8 | 0.13 | 0 | 50 | 3 | 23.47 | 0.00% | 93.00% | 0.7 | 0.76 | 114.58 | 2 | 0.04 |
| 41 | 2017 | 45 | 20.9 | 3 | 108.5 | 1619.7 | 0.22 | 0 | 45 | 4 | 17.35 | 0.00% | 100.00% | 0.96 | 0.33 | 119.53 | 3 | 0.12 |
| 42 | 2017 | 38 | 21.41 | 2 | 124.25 | 959.64 | 0.21 | 1 | 53 | 2 | 24.56 | 0.00% | 71.00% | 0.26 | 0.24 | 111.66 | 3 | 0.08 |
| 43 | 2017 | 18 | 19.68 | 4 | 99.51 | 847.44 | 0.22 | 1 | 53 | 5 | 25.87 | 0.00% | 73.00% | 1.05 | 0.36 | 109.56 | 2 | 0.13 |
| 44 | 2017 | 24 | 19.5 | 2 | 109.46 | 154.48 | 0.22 | 1 | 55 | 3 | 2.82 | 38.00% | 100.00% | 0.42 | 0.42 | 107.66 | 4 | 0.03 |
| 45 | 2017 | 35 | 20.76 | 2 | 119.2 | 735.18 | 0.18 | 1 | 72 | 3 | 10.01 | 0.00% | 45.00% | 0.94 | 0.26 | 114.10 | 2 | 0.04 |
| 46 | 2017 | 17 | 21.74 | 2 | 120.29 | 736.4 | 0.2 | 1 | 70 | 4 | 10.06 | 0.00% | 61.00% | 1.06 | 0.79 | 111.39 | 4 | 0.08 |
| 47 | 2017 | 34 | 47 | 5 | 274.86 | 627.05 | 0.16 | 0 | 30 | 3 | 1.66 | 0.00% | 100.00% | 0.26 | 0.24 | 116.82 | 2 | 0.09 |
| 48 | 2017 | 35 | 6.58 | 5 | 35.99 | 634.36 | 0.18 | 1 | 29 | 3 | 16.94 | 0.00% | 47.00% | 0.66 | 0.68 | 108.20 | 2 | 0.06 |
| 49 | 2017 | 22 | 50.88 | 5 | 255.6 | 793.56 | 0.18 | 0 | 34 | 3 | 2.02 | 19.00% | 76.00% | 0.47 | 0.37 | 116.55 | 4 | 0.08 |
| 50 | 2017 | 19 | 17.71 | 3 | 101.3 | 478.91 | 0.16 | 1 | 51 | 3 | 6.81 | 6.00% | 89.00% | 0.98 | 0.95 | 109.08 | 3 | 0.1 |
| 51 | 2017 | 48 | 3.64 | 4 | 23.98 | 635.43 | 0.23 | 1 | 31 | 3 | 16.85 | 0.00% | 62.00% | 0.69 | 0.69 | 114.78 | 3 | 0.06 |
| 52 | 2017 | 46 | 30.61 | 6 | 270.35 | 756.85 | 0.16 | 0 | 52 | 2 | 9.12 | 0.00% | 41.00% | 0.58 | 0.55 | 113.63 | 6 | 0.05 |
| 53 | 2017 | 20 | 19.26 | 5 | 102.69 | 1301.8 | 0.18 | 0 | 28 | 3 | 17.85 | 0.00% | 52.00% | 0.98 | 0.44 | 111.39 | 3 | 0.13 |
| 54 | 2017 | 32 | 2.74 | 3 | 37.41 | 671.2 | 0.21 | 1 | 54 | 2 | 17.28 | 0.00% | 100.00% | 0.26 | 0.3 | 111.80 | 2 | 0.08 |
| 55 | 2017 | 28 | 3.95 | 4 | 32.66 | 500.01 | 0.22 | 1 | 54 | 2 | 6.73 | 7.00% | 79.00% | 0.24 | 0.22 | 114.38 | 2 | 0.05 |
| 56 | 2017 | 37 | 19.22 | 5 | 100.47 | 849.29 | 0.21 | 0 | 52 | 4 | 25.85 | 0.00% | 91.00% | 1.07 | 0.39 | 119.26 | 3 | 0.18 |
| 57 | 2017 | 52 | 19.95 | 4 | 87.78 | 1587.47 | 0.21 | 0 | 45 | 3 | 17.31 | 0.00% | 62.00% | 0.25 | 0.23 | 118.58 | 4 | 0.15 |
| 58 | 2017 | 53 | 16.01 | 4 | 91.16 | 957.44 | 0.2 | 0 | 50 | 3 | 24.46 | 0.00% | 80.00% | 0.25 | 0.21 | 110.37 | 2 | 0.09 |
| 59 | 2017 | 33 | 19.67 | 2 | 124.53 | 726.95 | 0.21 | 0 | 68 | 4 | 9.98 | 0.00% | 64.00% | 0.99 | 0.86 | 120.35 | 2 | 0.18 |
| 60 | 2017 | 22 | 22.45 | 3 | 107.91 | 586.05 | 0.19 | 0 | 67 | 3 | 12.39 | 0.00% | 35.00% | 1.09 | 1.1 | 109.76 | 4 | 0.12 |
| 61 | 2017 | 32 | 7.01 | 4 | 27.53 | 602.05 | 0.21 | 1 | 67 | 3 | 13.07 | 0.00% | 47.00% | 0.72 | 0.64 | 115.73 | 3 | 0.14 |
| 62 | 2017 | 32 | 18.96 | 4 | 89.91 | 358.9 | 0.2 | 0 | 43 | 3 | 11.76 | 30.00% | 54.00% | 0.98 | 0.29 | 114.31 | 1 | 0.13 |
| 63 | 2017 | 50 | 26 | 4 | 137.9 | 747.21 | 0.18 | 0 | 37 | 5 | 1.56 | 12.00% | 66.00% | 0.45 | 0.31 | 113.90 | 5 | 0.15 |
| 64 | 2017 | 39 | 6.38 | 4 | 29.86 | 210.73 | 0.21 | 1 | 49 | 5 | 3.45 | 10.00% | 64.00% | 0.45 | 0.54 | 110.44 | 5 | 0.21 |
| 65 | 2017 | 37 | 6.39 | 4 | 21.43 | 617.06 | 0.23 | 1 | 34 | 4 | 16.15 | 0.00% | 50.00% | 0.97 | 0.95 | 111.46 | 3 | 0.23 |
| 66 | 2017 | 43 | 27.48 | 3 | 139.16 | 728.56 | 0.19 | 0 | 57 | 3 | 22.53 | 0.00% | 71.00% | 0.24 | 0.26 | 106.03 | 2 | 0.06 |
| 67 | 2017 | 59 | 23.68 | 3 | 144.12 | 718.12 | 0.17 | 0 | 60 | 3 | 22.61 | 0.00% | 59.00% | 0.23 | 0.21 | 108.14 | 4 | 0.06 |
| 68 | 2017 | 52 | 27.96 | 3 | 140.7 | 933.59 | 0.15 | 0 | 46 | 4 | 18.02 | 0.00% | 73.00% | 0.68 | 0.75 | 106.10 | 1 | 0.06 |
| 69 | 2017 | 58 | 24.4 | 3 | 142.47 | 709.22 | 0.17 | 0 | 60 | 4 | 22.53 | 0.00% | 62.00% | 0.25 | 0.23 | 116.21 | 3 | 0.06 |
| 70 | 2017 | 48 | 26.5 | 2 | 142.38 | 206.75 | 0.19 | 0 | 57 | 4 | 4.47 | 5.00% | 66.00% | 0.42 | 0.47 | 113.36 | 2 | 0.05 |
| 71 | 2017 | 64 | 24.97 | 4 | 134.74 | 701.58 | 0.21 | 0 | 45 | 3 | 12.77 | 0.00% | 71.00% | 0.74 | 0.85 | 113.56 | 3 | 0.06 |
| 72 | 2017 | 44 | 25.65 | 2 | 146.99 | 723.82 | 0.19 | 0 | 61 | 4 | 22.54 | 0.00% | 69.00% | 0.26 | 0.22 | 109.08 | 3 | 0.1 |
| 73 | 2017 | 42 | 19.76 | 3 | 55.45 | 883.58 | 0.22 | 0 | 51 | 4 | 25.81 | 0.00% | 81.00% | 1.05 | 0.35 | 119.46 | 3 | 0.09 |
| 74 | 2017 | 47 | 21.96 | 3 | 59.82 | 883.87 | 0.22 | 0 | 50 | 4 | 25.75 | 0.00% | 87.00% | 0.65 | 0.6 | 115.39 | 3 | 0.17 |
| 75 | 2017 | 31 | 22.49 | 2 | 61.26 | 1692.36 | 0.2 | 1 | 55 | 3 | 10.45 | 0.00% | 57.00% | 0.47 | 0.56 | 117.02 | 3 | 0.1 |
| 76 | 2017 | 59 | 22.4 | 3 | 64.76 | 586.88 | 0.2 | 1 | 67 | 3 | 12.34 | 0.00% | 60.00% | 0.96 | 1.22 | 107.66 | 4 | 0.05 |
| 77 | 2017 | 70 | 21.67 | 4 | 58.97 | 855.71 | 0.22 | 0 | 49 | 4 | 25.8 | 0.00% | 81.00% | 1.07 | 0.44 | 115.53 | 4 | 0.13 |
| 78 | 2017 | 74 | 21.88 | 4 | 58.64 | 377.52 | 0.22 | 1 | 41 | 3 | 11.75 | 32.00% | 61.00% | 0.87 | 0.32 | 109.08 | 2 | 0.13 |
| 79 | 2017 | 36 | 21.25 | 4 | 55.84 | 355.87 | 0.2 | 0 | 43 | 3 | 11.79 | 30.00% | 59.00% | 0.25 | 0.21 | 115.33 | 3 | 0.16 |
| 80 | 2017 | 75 | 21.95 | 3 | 50.35 | 876.16 | 0.24 | 1 | 49 | 4 | 25.82 | 0.00% | 73.00% | 1 | 0.36 | 110.71 | 2 | 0.21 |
| 81 | 2017 | 73 | 23.63 | 2 | 119.07 | 193.45 | 0.18 | 1 | 51 | 4 | 2.86 | 39.00% | 69.00% | 0.47 | 0.41 | 106.91 | 5 | 0.11 |
| 82 | 2017 | 75 | 22.65 | 2 | 117.44 | 955.5 | 0.22 | 0 | 50 | 4 | 24.47 | 0.00% | 80.00% | 0.95 | 0.95 | 117.23 | 6 | 0.16 |
| 83 | 2017 | 44 | 26.12 | 2 | 111.09 | 981.96 | 0.2 | 0 | 50 | 3 | 24.5 | 0.00% | 83.00% | 0.6 | 0.55 | 116.07 | 2 | 0.17 |
| 84 | 2017 | 89 | 39.57 | 2 | 209.68 | 200.37 | 0.21 | 0 | 58 | 5 | 4.51 | 11.00% | 79.00% | 0.47 | 0.37 | 116.34 | 7 | 0.12 |
| 85 | 2017 | 50 | 24.12 | 3 | 77.05 | 705.63 | 0.23 | 0 | 69 | 4 | 10 | 0.00% | 55.00% | 0.69 | 0.58 | 113.90 | 3 | 0.13 |
| 86 | 2017 | 85 | 23.83 | 2 | 132.15 | 1214.61 | 0.18 | 0 | 64 | 4 | 70.8 | 0.00% | 51.00% | 1.04 | 0.4 | 111.87 | 2 | 0.03 |
| 87 | 2017 | 43 | 38 | 3 | 207.33 | 706.08 | 0.16 | 0 | 60 | 5 | 22.61 | 0.00% | 79.00% | 0.24 | 0.26 | 115.33 | 7 | 0.11 |
| 88 | 2017 | 32 | 24.2 | 2 | 70.77 | 1213.23 | 0.2 | 0 | 62 | 5 | 70.82 | 0.00% | 60.00% | 0.97 | 0.44 | 110.37 | 5 | 0.11 |
| 89 | 2017 | 48 | 40.06 | 3 | 210.32 | 834.38 | 0.15 | 0 | 62 | 5 | 10.14 | 0.00% | 48.00% | 0.98 | 0.77 | 113.29 | 7 | 0.04 |
| 90 | 2017 | 73 | 22.56 | 2 | 79.06 | 1321.71 | 0.21 | 1 | 30 | 5 | 17.87 | 0.00% | 75.00% | 0.95 | 0.38 | 111.05 | 6 | 0.19 |
| 91 | 2017 | 86 | 24.38 | 2 | 89.61 | 1216.85 | 0.18 | 1 | 64 | 4 | 70.8 | 0.00% | 60.00% | 1.08 | 0.47 | 109.15 | 7 | 0.09 |
| 92 | 2017 | 64 | 24.49 | 2 | 118.55 | 179.78 | 0.22 | 1 | 54 | 3 | 2.83 | 37.00% | 65.00% | 0.44 | 0.3 | 117.23 | 2 | 0.05 |
| 93 | 2017 | 56 | 25.83 | 3 | 124.1 | 168.97 | 0.17 | 0 | 55 | 3 | 2.86 | 44.00% | 57.00% | 0.44 | 0.39 | 112.82 | 3 | 0.06 |
| 94 | 2017 | 45 | 25.86 | 2 | 110.72 | 1303.54 | 0.23 | 1 | 30 | 4 | 17.79 | 0.00% | 81.00% | 0.93 | 0.43 | 109.15 | 3 | 0.24 |
| 95 | 2017 | 53 | 36.93 | 3 | 218.43 | 740.4 | 0.14 | 0 | 61 | 3 | 22.6 | 0.00% | 81.00% | 0.27 | 0.23 | 112.95 | 3 | 0.09 |
| 96 | 2017 | 58 | 22.54 | 2 | 132.11 | 724.97 | 0.23 | 1 | 71 | 4 | 9.95 | 0.00% | 55.00% | 0.91 | 0.28 | 120.28 | 6 | 0.18 |
| 97 | 2017 | 38 | 23.65 | 2 | 85.65 | 1310.4 | 0.21 | 1 | 30 | 5 | 17.89 | 0.00% | 52.00% | 1.08 | 0.45 | 114.44 | 6 | 0.12 |
| 98 | 2017 | 41 | 25.49 | 3 | 116.35 | 502.19 | 0.19 | 0 | 54 | 3 | 6.77 | 2.00% | 88.00% | 0.27 | 0.2 | 111.26 | 2 | 0.14 |
| 99 | 2017 | 34 | 38.97 | 2 | 217.3 | 214.54 | 0.2 | 0 | 58 | 3 | 4.4 | 13.00% | 72.00% | 0.43 | 0.45 | 118.72 | 2 | 0.09 |
| 100 | 2017 | 97 | 37.36 | 3 | 204.38 | 792.63 | 0.17 | 0 | 61 | 5 | 10.13 | 0.00% | 56.00% | 0.95 | 0.72 | 106.30 | 6 | 0.18 |
| 101 | 2017 | 60 | 23.4 | 2 | 117.96 | 475.01 | 0.19 | 0 | 52 | 2 | 6.8 | 0.00% | 87.00% | 0.23 | 0.27 | 116.28 | 4 | 0.11 |
| 102 | 2017 | 34 | 24.01 | 2 | 121.27 | 468.85 | 0.16 | 0 | 51 | 3 | 6.78 | 0.00% | 48.00% | 0.49 | 0.34 | 106.51 | 3 | 0.15 |
| 103 | 2017 | 35 | 23.61 | 3 | 88.91 | 618.8 | 0.2 | 0 | 57 | 3 | 12.37 | 0.00% | 33.00% | 0.98 | 0.86 | 110.98 | 2 | 0.11 |
| 104 | 2017 | 48 | 22.15 | 2 | 85.82 | 606.53 | 0.19 | 0 | 57 | 3 | 12.37 | 0.00% | 51.00% | 1.07 | 0.96 | 108.27 | 3 | 0.08 |
| 105 | 2017 | 54 | 23.4 | 2 | 121.53 | 504.32 | 0.2 | 1 | 52 | 3 | 6.75 | 1.00% | 56.00% | 0.47 | 0.4 | 117.43 | 3 | 0.19 |
| 106 | 2017 | 48 | 39.32 | 3 | 201.63 | 193.45 | 0.19 | 0 | 59 | 3 | 3.57 | 49.00% | 75.00% | 0.43 | 0.4 | 115.94 | 4 | 0.16 |
| 107 | 2017 | 70 | 22.01 | 3 | 77.05 | 500.64 | 0.21 | 1 | 53 | 3 | 6.68 | 0.00% | 77.00% | 0.98 | 0.89 | 107.46 | 2 | 0.11 |
| 108 | 2017 | 52 | 38.65 | 2 | 205.64 | 824.2 | 0.18 | 0 | 62 | 3 | 10.12 | 0.00% | 81.00% | 1.1 | 0.33 | 109.97 | 3 | 0.2 |
| 109 | 2017 | 94 | 23.1 | 2 | 131.04 | 648.96 | 0.19 | 0 | 33 | 3 | 16.17 | 0.00% | 47.00% | 1.05 | 0.82 | 117.50 | 3 | 0.07 |
| 110 | 2017 | 77 | 23.28 | 4 | 143.75 | 728 | 0.19 | 0 | 71 | 3 | 9.93 | 0.00% | 57.00% | 0.71 | 0.65 | 112.68 | 3 | 0.19 |
| 111 | 2017 | 40 | 22.35 | 2 | 139.01 | 592.1 | 0.17 | 0 | 47 | 3 | 13.07 | 0.00% | 42.00% | 0.75 | 0.6 | 115.19 | 2 | 0.15 |
| 112 | 2017 | 78 | 25.58 | 3 | 139.61 | 211.99 | 0.17 | 1 | 52 | 3 | 3.4 | 2.00% | 55.00% | 0.47 | 0.38 | 111.39 | 4 | 0.08 |
| 113 | 2017 | 87 | 23.1 | 3 | 127.04 | 746.36 | 0.2 | 0 | 78 | 2 | 12.94 | 0.00% | 97.00% | 0.75 | 0.75 | 118.51 | 3 | 0.17 |
| 114 | 2017 | 94 | 28.24 | 4 | 113.46 | 1609.65 | 0.19 | 0 | 46 | 4 | 17.39 | 0.00% | 100.00% | 0.9 | 0.33 | 115.60 | 6 | 0.19 |
| 115 | 2017 | 44 | 28.8 | 4 | 121.18 | 1611.19 | 0.17 | 0 | 44 | 4 | 17.38 | 0.00% | 100.00% | 0.99 | 0.31 | 120.21 | 6 | 0.24 |
| 116 | 2017 | 60 | 21.56 | 2 | 58.3 | 1525.45 | 0.21 | 0 | 38 | 3 | 13.42 | 0.00% | 68.00% | 0.26 | 0.29 | 111.32 | 5 | 0.27 |
| 117 | 2017 | 78 | 27.76 | 3 | 112.76 | 1592.87 | 0.18 | 0 | 45 | 4 | 17.31 | 0.00% | 79.00% | 0.4 | 0.51 | 114.92 | 5 | 0.5 |
| 118 | 2017 | 119 | 28.62 | 3 | 120.21 | 1204.09 | 0.16 | 0 | 63 | 3 | 70.85 | 0.00% | 56.00% | 1.06 | 0.34 | 116.75 | 4 | 0.2 |
| 119 | 2017 | 51 | 18.17 | 3 | 56.47 | 1710.09 | 0.17 | 0 | 56 | 2 | 10.56 | 0.00% | 53.00% | 0.46 | 0.48 | 108.61 | 5 | 0.2 |
| 120 | 2017 | 118 | 18.6 | 4 | 59.65 | 961.77 | 0.19 | 0 | 52 | 3 | 24.55 | 0.00% | 72.00% | 0.23 | 0.22 | 112.82 | 2 | 0.36 |
| 121 | 2017 | 61 | 22.39 | 3 | 124.51 | 638.69 | 0.15 | 0 | 33 | 3 | 16.11 | 0.00% | 57.00% | 0.94 | 0.91 | 112.54 | 3 | 0.41 |
| 122 | 2017 | 43 | 29.87 | 3 | 112.87 | 194.02 | 0.19 | 0 | 49 | 4 | 3.49 | 10.00% | 42.00% | 0.41 | 0.49 | 106.03 | 1 | 0.15 |
| 123 | 2017 | 107 | 19.49 | 2 | 54.68 | 732.52 | 0.21 | 1 | 71 | 5 | 10.01 | 0.00% | 62.00% | 1 | 0.96 | 114.31 | 5 | 0.16 |
| 124 | 2017 | 49 | 20.16 | 2 | 62.28 | 1222.72 | 0.2 | 1 | 62 | 3 | 70.86 | 0.00% | 62.00% | 0.98 | 0.43 | 116.21 | 3 | 0.19 |
| 125 | 2017 | 103 | 23.48 | 3 | 143.23 | 1301.19 | 0.2 | 0 | 28 | 5 | 17.76 | 0.00% | 72.00% | 1.04 | 0.4 | 109.02 | 5 | 0.18 |
| 126 | 2017 | 73 | 27 | 3 | 116.4 | 1312.51 | 0.19 | 0 | 53 | 3 | 17.81 | 0.00% | 52.00% | 1 | 0.39 | 111.87 | 3 | 0.48 |
| 127 | 2017 | 65 | 20.91 | 4 | 61.19 | 478.95 | 0.17 | 1 | 47 | 1 | 5.26 | 4.00% | 79.00% | 0.27 | 0.24 | 106.57 | 4 | 0.16 |
| 128 | 2017 | 72 | 25.6 | 3 | 141.92 | 761.14 | 0.17 | 0 | 57 | 3 | 7.8 | 0.00% | 58.00% | 1.08 | 0.43 | 109.63 | 2 | 0.31 |
| 129 | 2017 | 100 | 28.76 | 3 | 105.26 | 494.07 | 0.19 | 0 | 52 | 3 | 6.78 | 0.00% | 58.00% | 0.45 | 0.43 | 110.78 | 3 | 0.4 |
| 130 | 2017 | 112 | 20.71 | 2 | 56.03 | 1536.23 | 0.22 | 1 | 39 | 3 | 13.29 | 0.00% | 83.00% | 0.24 | 0.23 | 118.31 | 3 | 0.48 |
| 131 | 2017 | 89 | 18.92 | 4 | 73.27 | 1139.12 | 0.18 | 0 | 57 | 3 | 54.49 | 0.00% | 57.00% | 0.93 | 0.49 | 111.12 | 4 | 0.22 |
| 132 | 2017 | 119 | 30.15 | 3 | 118.73 | 1214.13 | 0.15 | 0 | 63 | 5 | 70.81 | 0.00% | 56.00% | 0.96 | 0.35 | 111.80 | 5 | 0.26 |
| 133 | 2017 | 57 | 16.36 | 3 | 92.09 | 481.69 | 0.18 | 0 | 45 | 3 | 5.16 | 0.00% | 76.00% | 0.24 | 0.28 | 115.73 | 2 | 0.13 |
| 134 | 2017 | 102 | 15.14 | 3 | 82.52 | 1547.02 | 0.22 | 1 | 40 | 3 | 13.33 | 0.00% | 81.00% | 0.46 | 0.49 | 119.19 | 3 | 0.44 |
| 135 | 2017 | 69 | 15.8 | 3 | 86.59 | 605.92 | 0.18 | 1 | 27 | 4 | 12.37 | 0.00% | 58.00% | 0.91 | 0.86 | 106.51 | 6 | 0.46 |
| 136 | 2017 | 88 | 15.95 | 4 | 78.34 | 459.26 | 0.16 | 0 | 46 | 3 | 5.12 | 5.00% | 69.00% | 0.94 | 1.07 | 110.92 | 3 | 0.36 |
| 137 | 2017 | 46 | 14.57 | 4 | 79.34 | 393.84 | 0.22 | 1 | 43 | 3 | 11.89 | 29.00% | 54.00% | 0.89 | 0.36 | 110.31 | 2 | 0.16 |
| 138 | 2017 | 81 | 14.39 | 3 | 81.19 | 579.16 | 0.17 | 0 | 37 | 3 | 12.37 | 0.00% | 47.00% | 1.03 | 0.85 | 112.21 | 3 | 0.19 |
| 139 | 2017 | 78 | 16.55 | 3 | 86.13 | 506.8 | 0.17 | 1 | 52 | 3 | 6.78 | 0.00% | 77.00% | 1.08 | 0.98 | 109.63 | 3 | 0.3 |
| 140 | 2017 | 92 | 17.13 | 3 | 75.07 | 878.58 | 0.21 | 0 | 51 | 3 | 25.87 | 0.00% | 83.00% | 1.03 | 0.33 | 109.63 | 3 | 0.58 |
| 141 | 2017 | 75 | 13.5 | 4 | 78.75 | 1512.79 | 0.2 | 1 | 40 | 3 | 13.35 | 0.00% | 82.00% | 0.46 | 0.4 | 110.78 | 3 | 0.26 |
| 142 | 2017 | 104 | 14.54 | 3 | 75.1 | 389.9 | 0.2 | 1 | 40 | 4 | 11.88 | 25.00% | 53.00% | 0.53 | 0.5 | 109.29 | 6 | 0.54 |
| 143 | 2017 | 54 | 15.23 | 2 | 75.79 | 843.63 | 0.23 | 1 | 51 | 3 | 25.75 | 0.00% | 81.00% | 0.7 | 0.61 | 109.56 | 2 | 0.29 |
| 144 | 2017 | 111 | 17.32 | 3 | 75.1 | 1125.39 | 0.2 | 1 | 59 | 4 | 54.53 | 0.00% | 60.00% | 0.96 | 0.41 | 105.90 | 6 | 0.38 |
| 145 | 2017 | 80 | 29.33 | 3 | 129.28 | 791.26 | 0.2 | 0 | 34 | 3 | 2.04 | 16.00% | 72.00% | 0.42 | 0.34 | 115.67 | 2 | 0.54 |
| 146 | 2017 | 84 | 14.63 | 3 | 78.01 | 633.82 | 0.18 | 1 | 31 | 1 | 16.93 | 0.00% | 60.00% | 0.72 | 0.76 | 106.57 | 5 | 0.43 |
| 147 | 2017 | 129 | 31.7 | 2 | 168.69 | 1005.79 | 0.21 | 1 | 59 | 3 | 31.84 | 0.00% | 49.00% | 0.95 | 0.92 | 119.40 | 3 | 0.38 |
| 148 | 2017 | 190 | 33.75 | 4 | 166.97 | 763.62 | 0.14 | 0 | 57 | 4 | 7.78 | 0.00% | 73.00% | 0.99 | 0.43 | 113.49 | 3 | 0.42 |
| 149 | 2017 | 149 | 32.79 | 2 | 180.63 | 1592.26 | 0.2 | 0 | 44 | 4 | 17.41 | 0.00% | 71.00% | 0.25 | 0.27 | 114.17 | 7 | 0.62 |
| 150 | 2017 | 105 | 15.04 | 2 | 90.22 | 796.5 | 0.17 | 1 | 64 | 2 | 10.07 | 0.00% | 71.00% | 1 | 0.35 | 114.99 | 6 | 0.46 |
| 151 | 2017 | 117 | 32.6 | 3 | 186.23 | 1395.52 | 0.19 | 0 | 32 | 5 | 23.12 | 0.00% | 58.00% | 1.04 | 0.34 | 118.31 | 6 | 0.5 |
| 152 | 2017 | 135 | 33.38 | 3 | 185.4 | 1364.9 | 0.19 | 0 | 35 | 4 | 23.15 | 0.00% | 47.00% | 0.94 | 0.34 | 111.93 | 6 | 0.52 |
| 153 | 2017 | 87 | 32.28 | 3 | 129.17 | 978.37 | 0.18 | 0 | 54 | 4 | 23.54 | 0.00% | 60.00% | 0.25 | 0.29 | 109.56 | 6 | 0.61 |
| 154 | 2017 | 146 | 31.11 | 3 | 129.76 | 499.53 | 0.16 | 0 | 37 | 3 | 1.24 | 16.00% | 46.00% | 0.91 | 0.4 | 108.68 | 2 | 0.3 |
| 155 | 2017 | 154 | 32.38 | 3 | 139.03 | 611.12 | 0.15 | 0 | 34 | 3 | 1.65 | 0.00% | 100.00% | 0.23 | 0.24 | 116.41 | 2 | 0.18 |
| 156 | 2017 | 168 | 33.86 | 2 | 177.09 | 682.98 | 0.17 | 0 | 54 | 3 | 17.33 | 0.00% | 59.00% | 0.27 | 0.24 | 112.88 | 3 | 0.26 |
| 157 | 2017 | 97 | 12.01 | 4 | 78.86 | 1396.41 | 0.21 | 0 | 34 | 3 | 23.18 | 0.00% | 79.00% | 1.07 | 0.39 | 110.03 | 3 | 0.46 |
| 158 | 2017 | 116 | 12.46 | 2 | 71.57 | 205.07 | 0.22 | 1 | 56 | 5 | 4.38 | 10.00% | 66.00% | 0.45 | 0.53 | 107.46 | 5 | 0.67 |
| 159 | 2017 | 202 | 13.19 | 3 | 86.76 | 730.54 | 0.21 | 0 | 77 | 3 | 13.01 | 0.00% | 56.00% | 1 | 0.34 | 118.38 | 3 | 0.59 |
| 160 | 2017 | 151 | 33.22 | 3 | 127.5 | 561.38 | 0.16 | 0 | 38 | 4 | 6.68 | 0.00% | 75.00% | 1.05 | 0.85 | 109.70 | 5 | 0.19 |
| 161 | 2017 | 110 | 31.79 | 3 | 165.25 | 606.01 | 0.14 | 0 | 49 | 4 | 13.03 | 0.00% | 39.00% | 0.72 | 0.65 | 111.19 | 3 | 0.21 |
| 162 | 2017 | 91 | 32.68 | 2 | 176.11 | 1620.4 | 0.18 | 0 | 44 | 3 | 17.43 | 0.00% | 75.00% | 0.43 | 0.42 | 116.55 | 4 | 0.39 |
| 163 | 2017 | 110 | 13.55 | 3 | 90.94 | 771.54 | 0.2 | 0 | 76 | 4 | 13.02 | 0.00% | 100.00% | 0.74 | 0.85 | 113.36 | 3 | 0.45 |
| 164 | 2017 | 167 | 30.06 | 3 | 137.48 | 972 | 0.19 | 0 | 51 | 4 | 23.49 | 0.00% | 52.00% | 0.27 | 0.24 | 116.75 | 3 | 0.31 |
| 165 | 2017 | 191 | 14.24 | 2 | 77.08 | 746.54 | 0.24 | 1 | 76 | 3 | 13.03 | 0.00% | 48.00% | 1.06 | 0.29 | 112.07 | 3 | 0.53 |
| 166 | 2017 | 230 | 32.83 | 3 | 168.75 | 755.61 | 0.21 | 0 | 75 | 3 | 13.03 | 0.00% | 56.00% | 1.09 | 0.91 | 114.92 | 3 | 0.57 |
| 167 | 2017 | 138 | 34.02 | 3 | 164.97 | 184.83 | 0.2 | 0 | 53 | 2 | 2.79 | 38.00% | 100.00% | 0.41 | 0.36 | 114.65 | 4 | 0.32 |
| 168 | 2017 | 191 | 31.46 | 4 | 131.45 | 560.33 | 0.15 | 0 | 42 | 4 | 6.78 | 0.00% | 62.00% | 0.98 | 0.42 | 113.63 | 3 | 0.31 |
| 169 | 2017 | 196 | 32.68 | 4 | 185.27 | 1030.64 | 0.17 | 0 | 55 | 5 | 31.91 | 0.00% | 100.00% | 0.27 | 0.21 | 111.59 | 3 | 0.47 |
| 170 | 2017 | 166 | 32.73 | 3 | 177.89 | 701.47 | 0.21 | 0 | 69 | 4 | 10.01 | 0.00% | 47.00% | 0.75 | 0.79 | 113.22 | 4 | 0.34 |
| 171 | 2017 | 166 | 14.19 | 3 | 79.29 | 743.21 | 0.21 | 1 | 75 | 4 | 13.04 | 0.00% | 40.00% | 0.98 | 0.29 | 119.60 | 2 | 0.59 |
| 172 | 2017 | 116 | 29.84 | 3 | 119.94 | 740.64 | 0.18 | 0 | 50 | 3 | 9.09 | 0.00% | 59.00% | 0.62 | 0.61 | 108.27 | 3 | 0.24 |
| 173 | 2017 | 314 | 25.3 | 3 | 112.11 | 1156.2 | 0.19 | 1 | 41 | 2 | 46.33 | 0.00% | 87.00% | 0.91 | 0.86 | 111.46 | 4 | 0.4 |
| 174 | 2017 | 258 | 25.58 | 4 | 108.89 | 881.32 | 0.18 | 1 | 38 | 3 | 37.87 | 0.00% | 100.00% | 0.69 | 0.79 | 109.97 | 3 | 0.27 |
| 175 | 2017 | 285 | 25.71 | 4 | 122.99 | 890.55 | 0.18 | 1 | 36 | 3 | 37.9 | 0.00% | 100.00% | 0.7 | 0.69 | 113.29 | 3 | 0.29 |
| 176 | 2017 | 270 | 23.56 | 2 | 118.01 | 899.62 | 0.16 | 1 | 38 | 2 | 37.95 | 0.00% | 100.00% | 0.65 | 0.62 | 113.16 | 2 | 0.54 |
| 177 | 2017 | 305 | 25.27 | 4 | 123.84 | 901.34 | 0.15 | 0 | 36 | 3 | 37.94 | 0.00% | 100.00% | 0.49 | 0.34 | 108.81 | 3 | 0.63 |
| 178 | 2017 | 155 | 25.3 | 3 | 108.54 | 891.37 | 0.18 | 1 | 38 | 4 | 37.89 | 0.00% | 100.00% | 0.41 | 0.3 | 107.46 | 2 | 0.33 |
| 179 | 2017 | 318 | 25.17 | 2 | 114.48 | 896.44 | 0.16 | 0 | 39 | 4 | 37.87 | 0.00% | 100.00% | 0.96 | 0.46 | 107.12 | 4 | 0.81 |
| 180 | 2017 | 302 | 24.21 | 2 | 103.99 | 877.16 | 0.17 | 0 | 37 | 3 | 37.98 | 0.00% | 100.00% | 0.94 | 0.34 | 111.87 | 3 | 0.33 |
| 181 | 2017 | 344 | 20.78 | 3 | 130.24 | 577.65 | 0.15 | 1 | 47 | 2 | 13.02 | 0.00% | 46.00% | 0.66 | 0.67 | 108.75 | 2 | 0.56 |
| 182 | 2017 | 286 | 20.04 | 4 | 136.18 | 957.37 | 0.21 | 1 | 51 | 2 | 24.52 | 0.00% | 78.00% | 0.23 | 0.28 | 111.26 | 2 | 0.74 |
| 183 | 2017 | 285 | 22.69 | 2 | 125.04 | 164.31 | 0.18 | 1 | 54 | 3 | 2.76 | 40.00% | 55.00% | 0.42 | 0.38 | 106.24 | 3 | 0.39 |
| 184 | 2017 | 319 | 22.81 | 3 | 131.93 | 1331.96 | 0.17 | 1 | 29 | 3 | 17.79 | 0.00% | 69.00% | 1.08 | 0.41 | 110.31 | 4 | 0.37 |
| 185 | 2017 | 180 | 23.68 | 2 | 127.21 | 1614.87 | 0.18 | 0 | 47 | 3 | 17.32 | 0.00% | 75.00% | 0.25 | 0.23 | 116.34 | 3 | 0.37 |
| 186 | 2017 | 346 | 21.84 | 3 | 118.44 | 735.03 | 0.2 | 0 | 69 | 2 | 9.97 | 0.00% | 48.00% | 0.93 | 0.34 | 118.65 | 3 | 0.94 |
| 187 | 2017 | 144 | 21.36 | 2 | 120.4 | 958.53 | 0.22 | 0 | 50 | 3 | 24.5 | 0.00% | 65.00% | 0.52 | 0.48 | 109.97 | 4 | 0.81 |
| 188 | 2017 | 240 | 22.84 | 4 | 117.11 | 576.04 | 0.17 | 0 | 52 | 3 | 13.03 | 0.00% | 49.00% | 0.65 | 0.64 | 116.89 | 3 | 0.57 |
| 189 | 2017 | 307 | 54.16 | 4 | 458.21 | 1132.24 | 0.17 | 0 | 41 | 3 | 46.46 | 0.00% | 85.00% | 0.66 | 0.5 | 112.68 | 3 | 0.21 |
| 190 | 2017 | 270 | 36.33 | 2 | 195.26 | 763.47 | 0.16 | 0 | 56 | 3 | 7.77 | 0.00% | 25.00% | 0.98 | 0.82 | 114.38 | 2 | 0.28 |
| 191 | 2017 | 258 | 36.58 | 3 | 192.69 | 639.74 | 0.16 | 0 | 31 | 3 | 16.15 | 0.00% | 45.00% | 1.08 | 1.11 | 116.48 | 3 | 0.26 |
| 192 | 2017 | 333 | 36.96 | 3 | 206.77 | 956.81 | 0.19 | 0 | 50 | 5 | 24.48 | 0.00% | 78.00% | 1.08 | 1 | 110.31 | 5 | 0.56 |
| 193 | 2017 | 358 | 51.16 | 5 | 461.95 | 1143.27 | 0.17 | 0 | 44 | 3 | 46.32 | 0.00% | 88.00% | 0.98 | 0.97 | 116.55 | 3 | 0.38 |
| 194 | 2017 | 184 | 54.92 | 4 | 471.16 | 1146.8 | 0.15 | 0 | 40 | 4 | 46.37 | 0.00% | 55.00% | 0.26 | 0.29 | 108.68 | 3 | 0.52 |
| 195 | 2017 | 181 | 53.95 | 5 | 472.16 | 1132.48 | 0.14 | 0 | 40 | 5 | 46.31 | 0.00% | 83.00% | 0.63 | 0.67 | 116.14 | 7 | 0.28 |
| 196 | 2017 | 182 | 55.41 | 3 | 478.49 | 1126.43 | 0.16 | 0 | 42 | 5 | 46.43 | 0.00% | 81.00% | 1.01 | 0.96 | 117.16 | 6 | 0.67 |
| 197 | 2017 | 164 | 36.59 | 2 | 199.44 | 783.14 | 0.19 | 0 | 55 | 3 | 7.8 | 0.00% | 63.00% | 1.07 | 0.48 | 111.39 | 3 | 0.59 |
| 198 | 2017 | 286 | 52.94 | 5 | 473.53 | 96.79 | 0.21 | 1 | 55 | 4 | 5.09 | 0.00% | 100.00% | 0.62 | 0.69 | 118.99 | 2 | 0.96 |
| 199 | 2017 | 238 | 55.17 | 4 | 477.29 | 1127.61 | 0.16 | 0 | 43 | 5 | 46.38 | 0.00% | 43.00% | 0.24 | 0.21 | 109.36 | 6 | 0.35 |
| 200 | 2017 | 355 | 38.09 | 4 | 200.07 | 1002.29 | 0.18 | 0 | 56 | 3 | 31.87 | 0.00% | 59.00% | 0.98 | 0.93 | 111.93 | 3 | 0.47 |
| 201 | 2017 | 150 | 53.28 | 5 | 466.7 | 101.93 | 0.22 | 1 | 59 | 3 | 5.12 | 0.00% | 100.00% | 0.63 | 0.63 | 115.39 | 3 | 0.59 |
| 202 | 2017 | 138 | 34.86 | 4 | 210.27 | 1595.33 | 0.18 | 0 | 44 | 3 | 17.32 | 0.00% | 65.00% | 0.27 | 0.27 | 109.42 | 2 | 0.32 |
| 203 | 2017 | 206 | 35 | 3 | 209.31 | 754 | 0.15 | 0 | 54 | 3 | 7.72 | 0.00% | 34.00% | 1.07 | 0.78 | 117.63 | 4 | 0.48 |
| 204 | 2017 | 260 | 54.16 | 3 | 478.19 | 1130.83 | 0.13 | 0 | 42 | 3 | 46.39 | 0.00% | 84.00% | 1.05 | 1.19 | 112.41 | 2 | 0.59 |
| 205 | 2017 | 259 | 34.84 | 2 | 194.06 | 623.16 | 0.18 | 0 | 29 | 5 | 16.9 | 0.00% | 38.00% | 0.75 | 0.69 | 107.86 | 5 | 0.63 |
| 206 | 2017 | 232 | 52.19 | 4 | 475.44 | 115.9 | 0.22 | 1 | 57 | 3 | 5.12 | 0.00% | 66.00% | 0.59 | 0.59 | 112.41 | 2 | 1.06 |
| 207 | 2017 | 295 | 37.73 | 3 | 205.31 | 188.73 | 0.19 | 0 | 54 | 3 | 2.81 | 43.00% | 80.00% | 0.48 | 0.43 | 109.49 | 2 | 0.91 |
| 208 | 2017 | 308 | 51.21 | 4 | 465.07 | 1140.97 | 0.16 | 0 | 41 | 3 | 46.42 | 0.00% | 89.00% | 0.66 | 0.52 | 114.38 | 3 | 0.51 |
| 209 | 2017 | 174 | 38.25 | 4 | 212.8 | 618.65 | 0.13 | 0 | 32 | 4 | 16.11 | 0.00% | 56.00% | 0.96 | 1.22 | 115.33 | 3 | 0.39 |
| 210 | 2017 | 137 | 34.37 | 4 | 191.38 | 202.11 | 0.16 | 1 | 50 | 4 | 3.42 | 10.00% | 48.00% | 0.47 | 0.5 | 108.07 | 6 | 1.12 |
| 211 | 2017 | 368 | 37.91 | 3 | 207.27 | 668.74 | 0.14 | 0 | 54 | 2 | 17.29 | 0.00% | 100.00% | 0.26 | 0.26 | 113.49 | 3 | 0.47 |
| 212 | 2017 | 169 | 53.99 | 4 | 465.7 | 1092.55 | 0.15 | 0 | 36 | 2 | 35.7 | 0.00% | 59.00% | 0.26 | 0.29 | 108.75 | 7 | 0.92 |
| 213 | 2017 | 172 | 16.4 | 4 | 93.35 | 111.41 | 0.22 | 1 | 49 | 2 | 3.9 | 0.00% | 43.00% | 0.65 | 0.64 | 111.46 | 3 | 0.47 |
| 214 | 2017 | 374 | 12.6 | 3 | 84.32 | 179.68 | 0.24 | 1 | 57 | 3 | 3.66 | 51.00% | 81.00% | 0.47 | 0.36 | 116.95 | 3 | 1.05 |
| 215 | 2017 | 250 | 16.08 | 4 | 101.86 | 123.14 | 0.2 | 1 | 52 | 4 | 3.97 | 0.00% | 74.00% | 0.55 | 0.54 | 116.89 | 3 | 0.53 |
| 216 | 2017 | 267 | 16.5 | 4 | 112.87 | 1424.62 | 0.18 | 1 | 55 | 2 | 1.31 | 0.00% | 59.00% | 1.1 | 0.43 | 114.51 | 3 | 0.76 |
| 217 | 2017 | 319 | 13.14 | 3 | 71.57 | 1027.01 | 0.2 | 1 | 58 | 3 | 31.87 | 0.00% | 100.00% | 0.26 | 0.29 | 120.28 | 4 | 0.99 |
| 218 | 2017 | 230 | 19.26 | 4 | 93.37 | 1448.44 | 0.19 | 1 | 57 | 5 | 1.2 | 0.00% | 72.00% | 0.99 | 0.47 | 108.88 | 4 | 0.29 |
| 219 | 2017 | 324 | 13.37 | 3 | 78.12 | 201.61 | 0.24 | 1 | 55 | 2 | 4.45 | 7.00% | 65.00% | 0.47 | 0.44 | 111.53 | 3 | 0.58 |
| 220 | 2017 | 385 | 15.96 | 4 | 95.35 | 1448.03 | 0.16 | 1 | 54 | 2 | 1.31 | 0.00% | 61.00% | 1.1 | 0.43 | 115.73 | 3 | 0.44 |
| 221 | 2017 | 212 | 12.6 | 2 | 79.1 | 655.43 | 0.24 | 1 | 39 | 3 | 9.9 | 0.00% | 72.00% | 0.69 | 0.63 | 116.21 | 2 | 0.93 |
| 222 | 2017 | 148 | 13.77 | 3 | 86.24 | 675.73 | 0.18 | 1 | 52 | 3 | 17.28 | 0.00% | 71.00% | 0.23 | 0.28 | 112.41 | 2 | 0.45 |
| 223 | 2017 | 238 | 15.45 | 2 | 74.16 | 815.87 | 0.18 | 1 | 60 | 3 | 10.17 | 0.00% | 52.00% | 1.09 | 0.74 | 107.93 | 3 | 0.5 |
| 224 | 2017 | 392 | 18.11 | 3 | 111.09 | 110.72 | 0.18 | 1 | 51 | 2 | 3.88 | 0.00% | 74.00% | 0.57 | 0.54 | 115.12 | 3 | 0.87 |
| 225 | 2017 | 386 | 19.26 | 5 | 113.29 | 1447.86 | 0.18 | 0 | 57 | 5 | 1.26 | 0.00% | 67.00% | 0.97 | 0.45 | 106.44 | 1 | 0.56 |
| 226 | 2017 | 348 | 19.14 | 4 | 110.28 | 1440.52 | 0.16 | 1 | 55 | 1 | 1.33 | 0.00% | 51.00% | 1.05 | 0.43 | 112.07 | 5 | 0.31 |
| 227 | 2017 | 143 | 11.56 | 2 | 72.7 | 749.35 | 0.22 | 1 | 75 | 2 | 12.95 | 0.00% | 63.00% | 1.07 | 0.96 | 115.26 | 2 | 0.87 |
| 228 | 2017 | 170 | 13.2 | 2 | 77.86 | 189.23 | 0.25 | 1 | 56 | 3 | 4.45 | 10.00% | 58.00% | 0.41 | 0.41 | 116.00 | 4 | 1.38 |
| 229 | 2017 | 414 | 44.81 | 2 | 1912.18 | 935.27 | 0.14 | 0 | 43 | 2 | 49.33 | 0.00% | 92.00% | 0.94 | 0.35 | 116.48 | 0 | 0.82 |
| 230 | 2017 | 223 | 43.81 | 3 | 1910.99 | 927.76 | 0.14 | 0 | 44 | 4 | 49.26 | 0.00% | 100.00% | 0.74 | 0.78 | 107.66 | 3 | 0.63 |
| 231 | 2017 | 396 | 44.01 | 3 | 1928.74 | 932.2 | 0.16 | 0 | 42 | 2 | 49.23 | 0.00% | 80.00% | 0.45 | 0.36 | 107.39 | 2 | 0.25 |
| 232 | 2017 | 256 | 45.57 | 3 | 1927.63 | 930.85 | 0.17 | 0 | 44 | 3 | 49.33 | 0.00% | 95.00% | 1.05 | 0.4 | 110.44 | 3 | 0.46 |
| 233 | 2017 | 201 | 5.52 | 2 | 144.01 | 723.47 | 0.22 | 1 | 72 | 2 | 10.05 | 0.00% | 62.00% | 0.72 | 0.81 | 112.82 | 2 | 0.77 |
| 234 | 2017 | 161 | 6.65 | 3 | 140.38 | 968.73 | 0.2 | 1 | 49 | 3 | 24.48 | 0.00% | 83.00% | 0.58 | 0.51 | 113.97 | 4 | 0.5 |
| 235 | 2017 | 428 | 5.89 | 2 | 145.32 | 730.11 | 0.21 | 1 | 70 | 2 | 9.93 | 0.00% | 63.00% | 0.97 | 0.27 | 119.53 | 2 | 1.05 |
| 236 | 2017 | 304 | 7.72 | 2 | 147.3 | 571.23 | 0.2 | 1 | 63 | 2 | 12.97 | 0.00% | 40.00% | 0.64 | 0.6 | 113.43 | 2 | 0.59 |
| 237 | 2017 | 328 | 43.61 | 3 | 1915.86 | 909.7 | 0.16 | 0 | 43 | 4 | 49.28 | 0.00% | 78.00% | 1.08 | 0.46 | 113.22 | 3 | 1.1 |
| 238 | 2017 | 405 | 44.25 | 3 | 1923.02 | 929.89 | 0.17 | 0 | 44 | 3 | 49.3 | 0.00% | 100.00% | 0.66 | 0.76 | 116.55 | 3 | 0.15 |
| 239 | 2017 | 407 | 6.08 | 2 | 139.05 | 488.22 | 0.24 | 1 | 53 | 2 | 6.75 | 2.00% | 56.00% | 0.42 | 0.3 | 114.24 | 3 | 0.83 |
| 240 | 2017 | 246 | 8.07 | 1 | 160.42 | 979.14 | 0.21 | 1 | 49 | 2 | 24.59 | 0.00% | 68.00% | 1.01 | 1.18 | 114.92 | 3 | 0.52 |
| 241 | 2017 | 336 | 42.48 | 4 | 1920.15 | 942.77 | 0.16 | 0 | 42 | 2 | 49.37 | 0.00% | 96.00% | 0.45 | 0.37 | 106.71 | 1 | 0.42 |
| 242 | 2017 | 177 | 7.34 | 2 | 141.46 | 1611.04 | 0.22 | 1 | 43 | 4 | 17.43 | 0.00% | 91.00% | 0.43 | 0.44 | 116.95 | 3 | 0.99 |
| 243 | 2017 | 271 | 46.1 | 2 | 1913.6 | 914.42 | 0.13 | 0 | 42 | 3 | 49.31 | 0.00% | 100.00% | 0.62 | 0.6 | 106.03 | 3 | 1 |
| 244 | 2017 | 382 | 5.8 | 2 | 146.73 | 1191.32 | 0.21 | 1 | 64 | 3 | 70.87 | 0.00% | 61.00% | 0.96 | 0.37 | 110.10 | 4 | 0.67 |
| 245 | 2017 | 325 | 5.64 | 3 | 139.83 | 591.99 | 0.18 | 1 | 46 | 4 | 12.34 | 0.00% | 35.00% | 0.99 | 0.85 | 106.64 | 3 | 0.42 |
| 246 | 2017 | 338 | 7.12 | 2 | 152.54 | 503.28 | 0.21 | 1 | 55 | 3 | 6.67 | 0.00% | 74.00% | 1.1 | 0.97 | 112.34 | 4 | 0.44 |
| 247 | 2017 | 235 | 8.23 | 3 | 149.95 | 982.18 | 0.21 | 1 | 51 | 3 | 24.59 | 0.00% | 77.00% | 0.94 | 0.97 | 109.56 | 4 | 0.73 |
| 248 | 2017 | 261 | 6.04 | 2 | 159.09 | 586.95 | 0.2 | 1 | 48 | 3 | 13.07 | 0.00% | 51.00% | 0.67 | 0.57 | 112.48 | 2 | 0.78 |
| 249 | 2017 | 476 | 20.29 | 4 | 131.26 | 976.89 | 0.19 | 1 | 50 | 3 | 24.55 | 0.00% | 65.00% | 0.54 | 0.54 | 110.92 | 4 | 0.69 |
| 250 | 2017 | 456 | 19.98 | 3 | 128.82 | 165.75 | 0.2 | 1 | 53 | 2 | 2.8 | 38.00% | 100.00% | 0.42 | 0.32 | 109.56 | 2 | 0.58 |
| 251 | 2017 | 409 | 19.25 | 3 | 146.45 | 1324.5 | 0.18 | 1 | 29 | 3 | 17.83 | 0.00% | 55.00% | 1.09 | 0.47 | 119.67 | 4 | 1.44 |
| 252 | 2017 | 329 | 19.65 | 4 | 128.95 | 1210.82 | 0.17 | 1 | 63 | 3 | 70.8 | 0.00% | 58.00% | 1.07 | 0.43 | 110.71 | 3 | 1.13 |
| 253 | 2017 | 240 | 18.87 | 3 | 130.19 | 584.01 | 0.19 | 0 | 49 | 3 | 13.03 | 0.00% | 46.00% | 0.74 | 0.78 | 113.90 | 2 | 0.42 |
| 254 | 2017 | 323 | 20.43 | 3 | 137.4 | 1601.72 | 0.18 | 0 | 46 | 4 | 17.35 | 0.00% | 100.00% | 0.92 | 0.32 | 116.14 | 4 | 1.42 |
| 255 | 2017 | 335 | 20.37 | 4 | 129.39 | 473.66 | 0.15 | 0 | 51 | 4 | 6.71 | 5.00% | 83.00% | 0.25 | 0.25 | 108.27 | 4 | 0.77 |
| 256 | 2017 | 231 | 18.24 | 3 | 140.53 | 592.71 | 0.19 | 0 | 37 | 3 | 12.42 | 0.00% | 32.00% | 1.01 | 0.86 | 108.34 | 2 | 1.12 |
| 257 | 2017 | 283 | 36 | 5 | 206.85 | 459.17 | 0.14 | 0 | 47 | 2 | 7.07 | 0.00% | 100.00% | 0.25 | 0.21 | 113.63 | 1 | 0.8 |
| 258 | 2017 | 375 | 34.28 | 4 | 199.17 | 153.98 | 0.2 | 0 | 53 | 3 | 3.62 | 0.00% | 68.00% | 0.42 | 0.49 | 109.76 | 3 | 0.55 |
| 259 | 2017 | 243 | 34.54 | 4 | 203.68 | 462.67 | 0.17 | 0 | 47 | 3 | 7.08 | 0.00% | 100.00% | 0.27 | 0.28 | 109.08 | 4 | 0.7 |
| 260 | 2017 | 347 | 37.04 | 4 | 187.29 | 771.19 | 0.21 | 0 | 55 | 3 | 11.79 | 0.00% | 83.00% | 0.53 | 0.55 | 109.76 | 3 | 0.57 |
| 261 | 2017 | 446 | 35.44 | 5 | 189.97 | 172.95 | 0.16 | 0 | 53 | 5 | 3.68 | 0.00% | 75.00% | 1.45 | 0.64 | 115.87 | 2 | 0.75 |
| 262 | 2017 | 455 | 33.13 | 3 | 201.57 | 431.29 | 0.18 | 0 | 47 | 3 | 7.08 | 0.00% | 100.00% | 0.26 | 0.27 | 106.17 | 3 | 0.54 |
| 263 | 2017 | 356 | 36.92 | 4 | 206.35 | 163.2 | 0.19 | 0 | 54 | 5 | 3.62 | 0.00% | 60.00% | 1.08 | 0.71 | 114.99 | 2 | 0.65 |
| 264 | 2017 | 370 | 34.13 | 4 | 189.84 | 619.11 | 0.18 | 0 | 45 | 4 | 8.75 | 0.00% | 84.00% | 0.98 | 0.82 | 113.22 | 2 | 0.28 |
| 265 | 2017 | 334 | 36.37 | 3 | 198.76 | 510.39 | 0.18 | 0 | 39 | 3 | 1.22 | 16.00% | 69.00% | 0.92 | 0.34 | 106.03 | 3 | 1.05 |
| 266 | 2017 | 326 | 33.24 | 5 | 192.32 | 784.03 | 0.16 | 0 | 55 | 3 | 11.78 | 0.00% | 71.00% | 0.55 | 0.67 | 114.72 | 2 | 1.56 |
| 267 | 2017 | 273 | 34.16 | 3 | 190.03 | 784.1 | 0.15 | 0 | 54 | 3 | 11.7 | 0.00% | 76.00% | 0.57 | 0.67 | 106.57 | 3 | 0.89 |
| 268 | 2017 | 385 | 36.56 | 5 | 195.17 | 433.36 | 0.16 | 0 | 45 | 3 | 7.15 | 0.00% | 100.00% | 0.26 | 0.24 | 109.83 | 4 | 0.68 |
| 269 | 2017 | 326 | 34.44 | 3 | 101.51 | 624.61 | 0.17 | 0 | 30 | 4 | 1.69 | 0.00% | 100.00% | 0.25 | 0.3 | 114.24 | 6 | 1.17 |
| 270 | 2017 | 393 | 35.94 | 4 | 87.67 | 775.17 | 0.21 | 0 | 32 | 4 | 2.06 | 22.00% | 67.00% | 0.46 | 0.36 | 110.78 | 3 | 0.87 |
| 271 | 2017 | 478 | 37.4 | 3 | 96.03 | 157.96 | 0.21 | 1 | 53 | 5 | 3.71 | 0.00% | 69.00% | 0.47 | 0.53 | 114.04 | 2 | 0.95 |
| 272 | 2017 | 517 | 38.52 | 2 | 97.49 | 485.54 | 0.18 | 0 | 37 | 3 | 1.22 | 15.00% | 43.00% | 1.08 | 0.41 | 114.31 | 3 | 0.47 |
| 273 | 2017 | 434 | 37.34 | 3 | 83.41 | 756.7 | 0.2 | 0 | 33 | 4 | 2.1 | 24.00% | 70.00% | 0.43 | 0.32 | 115.12 | 2 | 1.11 |
| 274 | 2017 | 512 | 35.59 | 3 | 99.99 | 631.36 | 0.18 | 0 | 33 | 5 | 1.73 | 0.00% | 65.00% | 0.24 | 0.2 | 112.00 | 5 | 0.31 |
| 275 | 2017 | 474 | 35.52 | 2 | 87.46 | 153.11 | 0.2 | 1 | 51 | 3 | 3.62 | 0.00% | 70.00% | 0.44 | 0.52 | 113.77 | 3 | 0.9 |
| 276 | 2017 | 357 | 36.38 | 3 | 85.87 | 477.66 | 0.19 | 0 | 38 | 3 | 1.21 | 16.00% | 54.00% | 1.09 | 0.41 | 108.20 | 3 | 0.91 |
| 277 | 2017 | 377 | 37.71 | 2 | 83.86 | 506.08 | 0.21 | 1 | 36 | 3 | 1.23 | 23.00% | 55.00% | 0.98 | 0.44 | 111.39 | 5 | 0.52 |
| 278 | 2017 | 506 | 35.68 | 2 | 97.97 | 485.35 | 0.21 | 1 | 37 | 3 | 1.31 | 20.00% | 86.00% | 1.1 | 0.34 | 110.98 | 4 | 0.78 |
| 279 | 2017 | 441 | 36.53 | 3 | 87.15 | 467.98 | 0.2 | 0 | 46 | 5 | 7.16 | 0.00% | 100.00% | 0.25 | 0.22 | 105.96 | 1 | 0.92 |
| 280 | 2017 | 491 | 34.31 | 3 | 88.54 | 170.23 | 0.18 | 0 | 52 | 2 | 3.64 | 0.00% | 78.00% | 1.05 | 0.71 | 111.26 | 4 | 0.36 |
| 281 | 2017 | 521 | 31.89 | 3 | 193.12 | 752.57 | 0.19 | 0 | 39 | 4 | 1.6 | 20.00% | 42.00% | 0.45 | 0.38 | 119.80 | 4 | 0.9 |
| 282 | 2017 | 540 | 30.88 | 2 | 176.8 | 955.35 | 0.2 | 1 | 51 | 4 | 23.5 | 0.00% | 73.00% | 1.28 | 0.48 | 106.44 | 3 | 0.81 |
| 283 | 2017 | 481 | 30.51 | 2 | 188.88 | 930.81 | 0.19 | 1 | 47 | 2 | 18.04 | 0.00% | 73.00% | 1.33 | 0.44 | 110.65 | 2 | 0.75 |
| 284 | 2017 | 481 | 30.59 | 3 | 174.24 | 923.62 | 0.17 | 0 | 47 | 3 | 18.15 | 0.00% | 75.00% | 1.29 | 0.37 | 114.10 | 3 | 0.67 |
| 285 | 2017 | 505 | 31.82 | 3 | 192.1 | 911.03 | 0.16 | 0 | 47 | 2 | 18.08 | 0.00% | 64.00% | 0.76 | 0.75 | 111.46 | 3 | 0.67 |
| 286 | 2017 | 245 | 30.16 | 3 | 191.99 | 938.81 | 0.16 | 0 | 46 | 4 | 18.09 | 0.00% | 67.00% | 0.23 | 0.23 | 107.86 | 5 | 0.88 |
| 287 | 2017 | 336 | 30.59 | 2 | 184.12 | 743.45 | 0.18 | 0 | 28 | 2 | 1.64 | 19.00% | 61.00% | 0.44 | 0.3 | 109.15 | 4 | 0.93 |
| 288 | 2017 | 389 | 30.41 | 4 | 181.5 | 940.14 | 0.18 | 0 | 48 | 3 | 18.05 | 0.00% | 79.00% | 0.25 | 0.21 | 106.85 | 2 | 0.55 |
| 289 | 2017 | 532 | 33.47 | 6 | 186.7 | 968.58 | 0.15 | 0 | 52 | 2 | 23.56 | 0.00% | 80.00% | 1.33 | 0.44 | 114.72 | 4 | 0.82 |
| 290 | 2017 | 534 | 35.33 | 5 | 199.39 | 761.27 | 0.2 | 0 | 33 | 3 | 2.03 | 22.00% | 70.00% | 0.49 | 0.31 | 119.40 | 3 | 1.48 |
| 291 | 2017 | 447 | 32.68 | 6 | 189.25 | 496.34 | 0.18 | 0 | 38 | 2 | 1.21 | 13.00% | 84.00% | 1.08 | 0.33 | 116.95 | 2 | 0.74 |
| 292 | 2017 | 214 | 35.11 | 6 | 196.71 | 642.76 | 0.15 | 0 | 34 | 3 | 1.6 | 0.00% | 85.00% | 0.28 | 0.22 | 113.63 | 2 | 1 |
| 293 | 2017 | 225 | 35.96 | 6 | 206.66 | 497.03 | 0.14 | 0 | 39 | 4 | 1.21 | 23.00% | 78.00% | 0.98 | 0.37 | 115.73 | 3 | 1.25 |
| 294 | 2017 | 560 | 36.71 | 6 | 197.19 | 623.81 | 0.17 | 0 | 33 | 2 | 1.65 | 0.00% | 61.00% | 0.27 | 0.22 | 115.73 | 3 | 1.53 |
| 295 | 2017 | 373 | 34.36 | 6 | 191.34 | 611.54 | 0.18 | 0 | 33 | 3 | 1.68 | 0.00% | 62.00% | 0.24 | 0.21 | 117.43 | 3 | 0.76 |
| 296 | 2017 | 270 | 33.37 | 5 | 196.65 | 767.17 | 0.18 | 0 | 31 | 5 | 2.01 | 15.00% | 57.00% | 0.43 | 0.37 | 108.95 | 2 | 1.54 |
| 297 | 2017 | 653 | 29.94 | 4 | 177.52 | 737.27 | 0.18 | 1 | 60 | 3 | 22.5 | 0.00% | 67.00% | 0.26 | 0.25 | 109.42 | 4 | 0.45 |
| 298 | 2017 | 669 | 32.66 | 4 | 172.71 | 684.5 | 0.19 | 1 | 45 | 3 | 12.88 | 0.00% | 74.00% | 0.8 | 0.79 | 118.79 | 4 | 0.62 |
| 299 | 2017 | 526 | 32.04 | 3 | 188.86 | 188.18 | 0.17 | 1 | 60 | 5 | 3.62 | 49.00% | 76.00% | 0.5 | 0.37 | 114.38 | 2 | 1.07 |
| 300 | 2017 | 338 | 33.12 | 5 | 184.57 | 202.81 | 0.18 | 1 | 59 | 2 | 3.69 | 52.00% | 67.00% | 0.46 | 0.34 | 110.78 | 1 | 0.95 |
| 301 | 2017 | 587 | 33.28 | 4 | 172.1 | 672.66 | 0.2 | 1 | 47 | 1 | 12.88 | 0.00% | 75.00% | 0.72 | 0.69 | 117.70 | 4 | 1.45 |
| 302 | 2017 | 627 | 30.47 | 4 | 183.51 | 185.75 | 0.18 | 1 | 58 | 2 | 3.63 | 50.00% | 87.00% | 0.45 | 0.43 | 110.44 | 3 | 1.05 |
| 303 | 2017 | 646 | 29.71 | 4 | 187.66 | 690.79 | 0.16 | 1 | 51 | 2 | 17.3 | 0.00% | 100.00% | 0.25 | 0.23 | 113.90 | 2 | 0.76 |
| 304 | 2017 | 514 | 20.22 | 3 | 253.88 | 900.62 | 0.18 | 1 | 48 | 2 | 18.14 | 0.00% | 67.00% | 0.74 | 0.7 | 113.36 | 4 | 2.19 |
| 305 | 2017 | 375 | 22.18 | 3 | 253.05 | 793.41 | 0.18 | 1 | 61 | 2 | 10.08 | 0.00% | 48.00% | 0.99 | 0.84 | 111.12 | 4 | 1.56 |
| 306 | 2017 | 266 | 22.93 | 4 | 256.36 | 905.58 | 0.16 | 1 | 44 | 3 | 18.11 | 0.00% | 68.00% | 1.45 | 0.36 | 110.24 | 2 | 0.88 |
| 307 | 2017 | 339 | 32.93 | 4 | 176.91 | 822.87 | 0.19 | 1 | 62 | 4 | 10.09 | 0.00% | 73.00% | 1.08 | 0.34 | 107.86 | 6 | 0.73 |
| 308 | 2017 | 397 | 23.48 | 2 | 256.99 | 904.21 | 0.15 | 1 | 46 | 3 | 18.05 | 0.00% | 66.00% | 1.32 | 0.38 | 111.46 | 3 | 0.79 |
| 309 | 2017 | 231 | 20.8 | 3 | 253.88 | 920.12 | 0.15 | 0 | 45 | 2 | 18.06 | 0.00% | 72.00% | 0.26 | 0.23 | 110.98 | 3 | 0.94 |
| 310 | 2017 | 398 | 21.68 | 3 | 257.06 | 746.93 | 0.17 | 1 | 28 | 3 | 1.58 | 12.00% | 59.00% | 0.43 | 0.39 | 117.02 | 2 | 1.07 |
| 311 | 2017 | 401 | 21.16 | 4 | 241.89 | 909.33 | 0.17 | 0 | 46 | 2 | 18.09 | 0.00% | 74.00% | 0.72 | 0.69 | 117.09 | 3 | 1.79 |
| 312 | 2017 | 388 | 21.19 | 3 | 244.52 | 696.44 | 0.22 | 0 | 45 | 5 | 12.9 | 0.00% | 75.00% | 0.69 | 0.86 | 120.28 | 3 | 1.94 |
| 313 | 2017 | 400 | 22.93 | 3 | 255.9 | 728 | 0.2 | 0 | 27 | 4 | 1.67 | 14.00% | 74.00% | 0.49 | 0.43 | 120.28 | 7 | 1.22 |
| 314 | 2017 | 656 | 22.1 | 2 | 256.32 | 713.85 | 0.16 | 0 | 58 | 2 | 22.5 | 0.00% | 76.00% | 0.24 | 0.23 | 116.68 | 2 | 0.92 |
| 315 | 2017 | 471 | 21.76 | 3 | 249.55 | 731.54 | 0.2 | 0 | 27 | 5 | 1.62 | 19.00% | 46.00% | 0.41 | 0.36 | 109.29 | 5 | 1.12 |
| 316 | 2017 | 404 | 20.31 | 3 | 249.48 | 909.59 | 0.19 | 0 | 45 | 3 | 18.04 | 0.00% | 65.00% | 0.24 | 0.22 | 112.14 | 3 | 0.84 |
| 317 | 2017 | 325 | 41.16 | 3 | 232.03 | 148.91 | 0.18 | 1 | 52 | 3 | 3.68 | 0.00% | 68.00% | 1.45 | 0.65 | 111.39 | 3 | 0.48 |
| 318 | 2017 | 275 | 39.82 | 3 | 234.51 | 438.78 | 0.15 | 0 | 46 | 3 | 7.15 | 0.00% | 100.00% | 0.27 | 0.24 | 107.66 | 3 | 0.28 |
| 319 | 2017 | 393 | 41.25 | 2 | 231.49 | 642.8 | 0.17 | 0 | 36 | 2 | 2.12 | 0.00% | 100.00% | 0.27 | 0.2 | 113.97 | 4 | 1.36 |
| 320 | 2017 | 528 | 40.96 | 4 | 239.8 | 165.07 | 0.17 | 0 | 55 | 2 | 3.61 | 0.00% | 75.00% | 1.4 | 0.57 | 106.78 | 3 | 0.7 |
| 321 | 2017 | 706 | 41.65 | 2 | 238.19 | 799.78 | 0.21 | 0 | 57 | 2 | 11.73 | 0.00% | 71.00% | 0.53 | 0.51 | 116.95 | 3 | 0.77 |
| 322 | 2017 | 246 | 40.72 | 3 | 242.11 | 780.03 | 0.21 | 0 | 57 | 4 | 11.71 | 0.00% | 70.00% | 0.57 | 0.53 | 116.89 | 4 | 1.1 |
| 323 | 2017 | 403 | 42.4 | 4 | 227.46 | 652.18 | 0.15 | 0 | 36 | 3 | 2.11 | 0.00% | 67.00% | 0.24 | 0.25 | 113.29 | 4 | 0.61 |
| 324 | 2017 | 620 | 41.9 | 2 | 228.36 | 146.97 | 0.2 | 1 | 54 | 2 | 3.72 | 0.00% | 72.00% | 1.41 | 0.51 | 115.80 | 2 | 1.26 |
| 325 | 2017 | 700 | 43.18 | 3 | 232.25 | 581.46 | 0.16 | 0 | 48 | 4 | 8.72 | 0.00% | 54.00% | 0.97 | 0.45 | 108.88 | 3 | 1.26 |
| 326 | 2017 | 302 | 41.72 | 2 | 244.85 | 148.36 | 0.22 | 1 | 54 | 4 | 3.75 | 0.00% | 76.00% | 1.06 | 0.92 | 112.54 | 6 | 1.26 |
| 327 | 2017 | 723 | 41.57 | 2 | 240.35 | 597.35 | 0.18 | 0 | 44 | 2 | 8.81 | 0.00% | 69.00% | 1.01 | 0.67 | 113.63 | 3 | 0.95 |
| 328 | 2017 | 341 | 41.65 | 4 | 228.86 | 682.28 | 0.16 | 0 | 37 | 3 | 2.15 | 0.00% | 100.00% | 0.26 | 0.2 | 107.59 | 3 | 0.55 |
| 329 | 2017 | 338 | 37.53 | 3 | 401.96 | 754.28 | 0.15 | 0 | 50 | 4 | 9.09 | 0.00% | 100.00% | 0.59 | 0.67 | 112.68 | 7 | 1.51 |
| 330 | 2017 | 342 | 35.1 | 4 | 385.81 | 719.77 | 0.17 | 0 | 26 | 3 | 1.62 | 16.00% | 34.00% | 0.47 | 0.42 | 113.09 | 4 | 1.24 |
| 331 | 2017 | 511 | 35.76 | 3 | 390.8 | 982.25 | 0.18 | 0 | 51 | 2 | 23.54 | 0.00% | 64.00% | 0.23 | 0.24 | 110.51 | 2 | 0.84 |
| 332 | 2017 | 771 | 35.81 | 3 | 401.96 | 977.33 | 0.16 | 0 | 53 | 3 | 23.53 | 0.00% | 82.00% | 0.77 | 0.69 | 113.63 | 4 | 0.95 |
| 333 | 2017 | 703 | 33.76 | 3 | 398.7 | 724.36 | 0.2 | 0 | 27 | 2 | 1.61 | 20.00% | 65.00% | 0.49 | 0.39 | 115.19 | 2 | 1.15 |
| 334 | 2017 | 743 | 34.83 | 4 | 404.01 | 975 | 0.17 | 0 | 54 | 2 | 23.44 | 0.00% | 55.00% | 0.25 | 0.21 | 114.51 | 3 | 1.11 |
| 335 | 2017 | 565 | 33.92 | 3 | 391.04 | 772.43 | 0.14 | 0 | 51 | 3 | 9.09 | 0.00% | 60.00% | 0.6 | 0.66 | 110.37 | 3 | 1.17 |
| 336 | 2017 | 546 | 36.88 | 4 | 384.75 | 756.07 | 0.18 | 0 | 49 | 3 | 9 | 0.00% | 100.00% | 0.54 | 0.68 | 111.39 | 3 | 0.67 |
| 337 | 2017 | 610 | 35.24 | 3 | 400.44 | 561.81 | 0.18 | 0 | 41 | 5 | 6.78 | 0.00% | 55.00% | 0.98 | 0.4 | 108.95 | 2 | 1.05 |
| 338 | 2017 | 606 | 35.83 | 3 | 392.93 | 746.04 | 0.15 | 0 | 52 | 3 | 9.07 | 0.00% | 100.00% | 0.6 | 0.49 | 106.51 | 2 | 1.08 |
| 339 | 2017 | 610 | 33.44 | 2 | 401.02 | 955.48 | 0.15 | 0 | 54 | 4 | 23.48 | 0.00% | 77.00% | 0.68 | 0.72 | 115.73 | 2 | 0.71 |
| 340 | 2017 | 349 | 33.79 | 2 | 404.2 | 955.46 | 0.18 | 0 | 50 | 4 | 23.49 | 0.00% | 88.00% | 0.76 | 0.81 | 106.51 | 2 | 0.81 |
| 341 | 2017 | 639 | 23 | 3 | 128.28 | 699.32 | 0.18 | 0 | 55 | 4 | 17.36 | 0.00% | 60.00% | 0.27 | 0.25 | 116.28 | 6 | 1.48 |
| 342 | 2017 | 729 | 20.75 | 4 | 129.87 | 1008.84 | 0.19 | 1 | 59 | 5 | 31.92 | 0.00% | 85.00% | 0.55 | 0.67 | 117.09 | 4 | 0.85 |
| 343 | 2017 | 270 | 22.2 | 3 | 122.27 | 1400.83 | 0.2 | 1 | 35 | 2 | 23.23 | 0.00% | 74.00% | 0.91 | 0.38 | 119.53 | 4 | 0.84 |
| 344 | 2017 | 389 | 24.37 | 2 | 130.67 | 793.91 | 0.18 | 1 | 55 | 2 | 7.85 | 0.00% | 39.00% | 1.09 | 0.68 | 117.50 | 2 | 0.79 |
| 345 | 2017 | 638 | 21.02 | 4 | 136.24 | 762.9 | 0.2 | 0 | 76 | 4 | 12.98 | 0.00% | 49.00% | 1 | 0.36 | 116.21 | 3 | 1.9 |
| 346 | 2017 | 469 | 22.3 | 2 | 126.49 | 998 | 0.22 | 1 | 58 | 3 | 31.94 | 0.00% | 100.00% | 0.24 | 0.21 | 113.16 | 3 | 3.16 |
| 347 | 2017 | 357 | 21.94 | 3 | 126.38 | 750.58 | 0.23 | 1 | 75 | 4 | 12.91 | 0.00% | 92.00% | 0.77 | 0.67 | 113.90 | 4 | 1.04 |
| 348 | 2017 | 761 | 24.17 | 3 | 127.12 | 214.12 | 0.22 | 1 | 50 | 2 | 3.38 | 9.00% | 48.00% | 0.45 | 0.41 | 115.12 | 2 | 1.48 |
| 349 | 2017 | 580 | 20.28 | 3 | 127.78 | 1374.76 | 0.22 | 1 | 35 | 2 | 23.19 | 0.00% | 53.00% | 1.1 | 0.36 | 117.16 | 2 | 0.69 |
| 350 | 2017 | 348 | 22.6 | 3 | 127.28 | 622.92 | 0.18 | 1 | 32 | 2 | 16.83 | 0.00% | 65.00% | 0.74 | 0.72 | 115.73 | 4 | 0.93 |
| 351 | 2017 | 544 | 20.46 | 3 | 121.51 | 168.08 | 0.22 | 1 | 60 | 3 | 3.65 | 52.00% | 70.00% | 0.49 | 0.38 | 119.12 | 4 | 2.33 |
| 352 | 2017 | 396 | 35.53 | 4 | 213.49 | 611.93 | 0.16 | 0 | 47 | 3 | 8.8 | 0.00% | 53.00% | 1.07 | 0.48 | 109.36 | 4 | 1.15 |
| 353 | 2017 | 613 | 38.24 | 5 | 216.15 | 581.35 | 0.16 | 0 | 48 | 2 | 8.83 | 0.00% | 70.00% | 0.95 | 0.91 | 109.15 | 3 | 0.99 |
| 354 | 2017 | 689 | 36.8 | 6 | 215.78 | 132.89 | 0.21 | 0 | 51 | 2 | 3.64 | 0.00% | 72.00% | 0.43 | 0.47 | 106.17 | 2 | 1.95 |
| 355 | 2017 | 648 | 20.79 | 2 | 140.92 | 780.55 | 0.17 | 1 | 56 | 2 | 7.84 | 0.00% | 59.00% | 0.98 | 0.43 | 114.58 | 2 | 0.99 |
| 356 | 2017 | 610 | 35.69 | 6 | 221.5 | 584.49 | 0.16 | 0 | 44 | 2 | 8.73 | 0.00% | 56.00% | 1.06 | 0.47 | 112.88 | 1 | 1.62 |
| 357 | 2017 | 368 | 37.48 | 4 | 203.85 | 144.77 | 0.17 | 1 | 54 | 3 | 3.6 | 0.00% | 58.00% | 0.98 | 0.69 | 108.68 | 2 | 0.55 |
| 358 | 2017 | 440 | 38.18 | 5 | 219.61 | 794.91 | 0.2 | 0 | 55 | 3 | 11.73 | 0.00% | 75.00% | 0.61 | 0.49 | 110.78 | 4 | 3.27 |
| 359 | 2017 | 461 | 35.49 | 5 | 219.35 | 810.77 | 0.2 | 0 | 55 | 4 | 11.77 | 0.00% | 67.00% | 0.52 | 0.52 | 119.67 | 2 | 2.68 |
| 360 | 2017 | 750 | 38.4 | 6 | 208.9 | 443.87 | 0.17 | 0 | 46 | 3 | 7.05 | 0.00% | 73.00% | 0.27 | 0.3 | 108.75 | 3 | 1.73 |
| 361 | 2017 | 291 | 86.57 | 4 | 589.27 | 926.45 | 0.13 | 0 | 44 | 5 | 49.28 | 0.00% | 86.00% | 1.06 | 0.39 | 110.71 | 2 | 1.9 |
| 362 | 2017 | 575 | 24.24 | 3 | 140.7 | 454.9 | 0.18 | 0 | 53 | 2 | 9.27 | 0.00% | 100.00% | 0.25 | 0.21 | 109.70 | 2 | 1.74 |
| 363 | 2017 | 554 | 21.34 | 2 | 146.23 | 141.36 | 0.24 | 1 | 61 | 5 | 4.83 | 0.00% | 85.00% | 1.08 | 0.77 | 107.19 | 1 | 1.35 |
| 364 | 2017 | 617 | 24.05 | 3 | 134.76 | 1352.41 | 0.19 | 0 | 49 | 3 | 1.01 | 0.00% | 48.00% | 1.09 | 0.34 | 111.05 | 3 | 1.11 |
| 365 | 2017 | 650 | 20.39 | 4 | 132.04 | 152.48 | 0.21 | 1 | 58 | 4 | 4.78 | 0.00% | 84.00% | 1.3 | 0.54 | 117.29 | 4 | 1.79 |
| 366 | 2017 | 741 | 24.43 | 3 | 130.91 | 150.5 | 0.21 | 1 | 60 | 4 | 4.81 | 0.00% | 91.00% | 1.53 | 0.64 | 109.83 | 5 | 0.88 |
| 367 | 2017 | 696 | 22.15 | 3 | 138.59 | 146.67 | 0.21 | 1 | 58 | 4 | 4.73 | 0.00% | 100.00% | 0.49 | 0.39 | 109.08 | 2 | 1.55 |
| 368 | 2017 | 601 | 22.76 | 4 | 140.36 | 1359.6 | 0.16 | 0 | 51 | 2 | 1.02 | 0.00% | 51.00% | 1.03 | 0.34 | 117.63 | 1 | 1.31 |
| 369 | 2017 | 532 | 20.59 | 4 | 132.54 | 1363.49 | 0.16 | 0 | 51 | 4 | 1 | 0.00% | 51.00% | 1.1 | 0.34 | 116.55 | 4 | 3.18 |
| 370 | 2017 | 993 | 30.47 | 9 | 344.36 | 1351.17 | 0.15 | 0 | 48 | 2 | 1.05 | 0.00% | 50.00% | 0.94 | 0.43 | 116.41 | 2 | 1.77 |
| 371 | 2017 | 628 | 31.96 | 9 | 346.01 | 1347.95 | 0.18 | 0 | 50 | 2 | 1.04 | 0.00% | 63.00% | 1.05 | 0.37 | 110.51 | 2 | 3.23 |
| 372 | 2017 | 435 | 33.24 | 8 | 336.55 | 1347.89 | 0.14 | 0 | 50 | 3 | 0.91 | 0.00% | 55.00% | 1.06 | 0.39 | 106.78 | 3 | 0.79 |
| 373 | 2017 | 494 | 31.09 | 8 | 327.76 | 1453.36 | 0.14 | 1 | 57 | 2 | 1.21 | 0.00% | 52.00% | 1.1 | 0.42 | 111.87 | 2 | 2.07 |
| 374 | 2017 | 677 | 31.31 | 8 | 329.35 | 1418.39 | 0.16 | 0 | 55 | 2 | 1.34 | 0.00% | 52.00% | 1.08 | 0.4 | 107.52 | 2 | 0.7 |
| 375 | 2017 | 525 | 33.39 | 7 | 329.45 | 1355.37 | 0.15 | 0 | 50 | 3 | 1.01 | 0.00% | 50.00% | 1 | 0.4 | 115.12 | 3 | 0.66 |
| 376 | 2017 | 853 | 34.21 | 9 | 333.89 | 1359.27 | 0.17 | 0 | 48 | 3 | 1.02 | 0.00% | 52.00% | 0.92 | 0.43 | 111.93 | 2 | 2.12 |
| 377 | 2017 | 616 | 31.48 | 9 | 344.1 | 1438.54 | 0.14 | 0 | 57 | 3 | 1.27 | 0.00% | 63.00% | 0.97 | 0.46 | 113.63 | 2 | 1.33 |
| 378 | 2017 | 852 | 37.42 | 2 | 217.19 | 149.26 | 0.2 | 1 | 60 | 3 | 4.81 | 0.00% | 69.00% | 0.99 | 0.67 | 111.46 | 3 | 1.43 |
| 379 | 2017 | 903 | 37.37 | 2 | 218.32 | 156.63 | 0.21 | 1 | 61 | 3 | 4.71 | 0.00% | 89.00% | 1.54 | 0.69 | 110.03 | 3 | 1.91 |
| 380 | 2017 | 665 | 38.36 | 2 | 203.22 | 509.46 | 0.18 | 1 | 43 | 5 | 1.66 | 22.00% | 49.00% | 1 | 0.41 | 113.02 | 6 | 1.48 |
| 381 | 2017 | 486 | 36.63 | 2 | 211.73 | 466.92 | 0.18 | 0 | 51 | 2 | 9.3 | 0.00% | 100.00% | 0.26 | 0.3 | 109.42 | 3 | 1.7 |
| 382 | 2017 | 1037 | 36.4 | 3 | 203.61 | 645.18 | 0.18 | 0 | 40 | 3 | 2.22 | 0.00% | 68.00% | 0.24 | 0.26 | 108.81 | 4 | 2.59 |
| 383 | 2017 | 963 | 35.56 | 1 | 205.42 | 182.37 | 0.22 | 0 | 59 | 3 | 4.71 | 0.00% | 76.00% | 1.1 | 0.93 | 115.73 | 3 | 2.77 |
| 384 | 2017 | 398 | 37.05 | 2 | 220.52 | 658.52 | 0.18 | 0 | 39 | 5 | 2.13 | 0.00% | 67.00% | 0.23 | 0.24 | 107.59 | 2 | 3.2 |
| 385 | 2017 | 937 | 36.77 | 3 | 224.16 | 493.25 | 0.17 | 0 | 54 | 4 | 9.2 | 0.00% | 100.00% | 0.24 | 0.29 | 112.07 | 2 | 1.84 |
| 386 | 2017 | 759 | 36.2 | 2 | 213.23 | 500.64 | 0.17 | 0 | 44 | 2 | 1.66 | 21.00% | 65.00% | 1.09 | 0.38 | 107.19 | 3 | 2.67 |
| 387 | 2017 | 715 | 38.56 | 2 | 216.95 | 525.08 | 0.19 | 0 | 42 | 2 | 1.58 | 25.00% | 100.00% | 0.98 | 0.48 | 107.39 | 4 | 1.53 |
| 388 | 2017 | 659 | 37.83 | 2 | 210.95 | 174.78 | 0.17 | 0 | 60 | 2 | 4.83 | 0.00% | 100.00% | 0.42 | 0.54 | 106.30 | 4 | 2.57 |
| 389 | 2017 | 1016 | 38.01 | 2 | 207.51 | 173.89 | 0.2 | 0 | 59 | 3 | 4.73 | 0.00% | 100.00% | 0.48 | 0.38 | 106.71 | 4 | 1.28 |
| 390 | 2017 | 1080 | 29.69 | 5 | 261.08 | 1022.2 | 0.18 | 0 | 56 | 3 | 31.94 | 0.00% | 86.00% | 0.59 | 0.66 | 118.24 | 2 | 2.45 |
| 391 | 2017 | 882 | 30.99 | 4 | 265.91 | 993.5 | 0.19 | 0 | 58 | 4 | 31.93 | 0.00% | 90.00% | 0.54 | 0.47 | 115.87 | 3 | 1.65 |
| 392 | 2017 | 866 | 29.7 | 6 | 271.57 | 207.9 | 0.21 | 1 | 56 | 4 | 4.52 | 11.00% | 81.00% | 0.46 | 0.54 | 118.11 | 4 | 1.48 |
| 393 | 2017 | 962 | 31.95 | 4 | 273.62 | 664.72 | 0.18 | 0 | 40 | 4 | 9.89 | 0.00% | 84.00% | 0.79 | 0.86 | 112.14 | 3 | 2.4 |
| 394 | 2017 | 637 | 31.45 | 5 | 272.59 | 215.78 | 0.17 | 1 | 58 | 5 | 4.42 | 5.00% | 63.00% | 0.43 | 0.38 | 115.73 | 3 | 2.25 |
| 395 | 2017 | 1063 | 31.86 | 5 | 261.93 | 810.01 | 0.15 | 0 | 63 | 3 | 10.09 | 0.00% | 83.00% | 1.09 | 0.4 | 113.70 | 3 | 1.57 |
| 396 | 2017 | 571 | 33.08 | 5 | 258.38 | 671.27 | 0.16 | 0 | 51 | 2 | 17.37 | 0.00% | 65.00% | 0.23 | 0.3 | 109.02 | 2 | 1.52 |
| 397 | 2017 | 979 | 32.78 | 5 | 272.81 | 1018.37 | 0.22 | 0 | 56 | 4 | 31.88 | 0.00% | 79.00% | 0.53 | 0.61 | 118.72 | 3 | 3.18 |
| 398 | 2017 | 620 | 43.59 | 7 | 266.52 | 911.92 | 0.17 | 1 | 44 | 4 | 49.23 | 0.00% | 100.00% | 0.69 | 0.61 | 117.23 | 4 | 1.23 |
| 399 | 2017 | 747 | 44 | 8 | 270.74 | 888.35 | 0.16 | 1 | 37 | 3 | 37.9 | 0.00% | 100.00% | 0.49 | 0.44 | 115.12 | 3 | 1.58 |
| 400 | 2017 | 957 | 44.92 | 9 | 263.32 | 927.39 | 0.14 | 1 | 44 | 3 | 49.29 | 0.00% | 84.00% | 0.43 | 0.41 | 110.17 | 3 | 1.89 |
| 401 | 2017 | 1061 | 45.15 | 9 | 254.79 | 898.64 | 0.18 | 1 | 37 | 4 | 37.87 | 0.00% | 100.00% | 1.05 | 0.37 | 116.75 | 4 | 0.89 |
| 402 | 2017 | 789 | 46.25 | 8 | 267.63 | 874.79 | 0.17 | 1 | 37 | 2 | 37.95 | 0.00% | 100.00% | 0.64 | 0.68 | 113.56 | 3 | 1.49 |
| 403 | 2017 | 593 | 45.05 | 8 | 258.56 | 867.57 | 0.17 | 1 | 39 | 2 | 37.93 | 0.00% | 100.00% | 0.41 | 0.44 | 116.89 | 2 | 1.91 |
| 404 | 2017 | 1060 | 45.45 | 7 | 253.36 | 924.58 | 0.17 | 1 | 42 | 2 | 49.34 | 0.00% | 81.00% | 0.41 | 0.37 | 114.99 | 3 | 3.03 |
| 405 | 2017 | 941 | 45.54 | 8 | 266.63 | 858.52 | 0.17 | 1 | 36 | 2 | 37.97 | 0.00% | 100.00% | 0.96 | 0.38 | 107.93 | 2 | 1.53 |
| 406 | 2017 | 838 | 85.17 | 7 | 844.07 | 123.34 | 0.19 | 1 | 58 | 5 | 5.07 | 0.00% | 66.00% | 0.54 | 0.67 | 119.60 | 2 | 2.2 |
| 407 | 2017 | 1227 | 85.35 | 6 | 843.76 | 1131.13 | 0.15 | 0 | 40 | 2 | 46.35 | 0.00% | 39.00% | 0.23 | 0.24 | 110.85 | 2 | 2.53 |
| 408 | 2017 | 638 | 87.28 | 7 | 849.94 | 105.17 | 0.2 | 1 | 58 | 4 | 5.13 | 0.00% | 100.00% | 0.63 | 0.53 | 118.24 | 3 | 4.14 |
| 409 | 2017 | 818 | 87.25 | 7 | 848.55 | 1100.25 | 0.16 | 0 | 35 | 4 | 35.73 | 0.00% | 62.00% | 0.96 | 1.19 | 115.26 | 3 | 1.24 |
| 410 | 2017 | 1010 | 85.76 | 7 | 847.07 | 1149.48 | 0.17 | 0 | 42 | 4 | 46.4 | 0.00% | 43.00% | 0.25 | 0.24 | 114.31 | 3 | 1.03 |
| 411 | 2017 | 1279 | 86.38 | 7 | 834.71 | 94.29 | 0.23 | 1 | 56 | 3 | 5.19 | 0.00% | 100.00% | 0.61 | 0.54 | 108.68 | 3 | 3.46 |
| 412 | 2017 | 701 | 84.81 | 8 | 850.44 | 1081.87 | 0.16 | 0 | 37 | 2 | 35.64 | 0.00% | 57.00% | 0.25 | 0.24 | 111.93 | 2 | 1.2 |
| 413 | 2017 | 829 | 85.08 | 7 | 846.31 | 1131.11 | 0.17 | 0 | 43 | 3 | 46.32 | 0.00% | 94.00% | 0.61 | 0.64 | 106.10 | 4 | 1.22 |
| 414 | 2017 | 970 | 86.36 | 8 | 833.34 | 122.36 | 0.21 | 0 | 55 | 5 | 5.14 | 0.00% | 71.00% | 0.57 | 0.6 | 110.37 | 2 | 4.15 |
| 415 | 2017 | 537 | 87.32 | 6 | 839.82 | 1076.93 | 0.15 | 0 | 37 | 4 | 35.63 | 0.00% | 72.00% | 0.56 | 0.6 | 109.36 | 4 | 3.9 |
| 416 | 2017 | 591 | 83.84 | 7 | 839.96 | 123.25 | 0.23 | 0 | 58 | 4 | 5.13 | 0.00% | 57.00% | 0.55 | 0.69 | 118.38 | 3 | 1.08 |
| 417 | 2017 | 1277 | 85.64 | 7 | 833.69 | 1081.65 | 0.15 | 0 | 37 | 2 | 35.73 | 0.00% | 73.00% | 0.56 | 0.67 | 112.27 | 1 | 1.91 |
| 418 | 2017 | 1029 | 52.18 | 7 | 309 | 96.73 | 0.21 | 1 | 49 | 2 | 4.01 | 0.00% | 56.00% | 0.56 | 0.67 | 113.77 | 3 | 2.15 |
| 419 | 2017 | 745 | 48.85 | 6 | 321.12 | 1113.85 | 0.15 | 0 | 38 | 3 | 35.62 | 0.00% | 74.00% | 0.66 | 0.68 | 110.71 | 3 | 1.03 |
| 420 | 2017 | 1226 | 51.81 | 5 | 312.55 | 1111.72 | 0.18 | 0 | 35 | 2 | 35.76 | 0.00% | 67.00% | 0.98 | 0.85 | 108.47 | 1 | 4.1 |
| 421 | 2017 | 919 | 49.01 | 5 | 318.55 | 115.85 | 0.21 | 1 | 51 | 3 | 4.02 | 0.00% | 75.00% | 0.62 | 0.67 | 106.57 | 3 | 2.28 |
| 422 | 2017 | 879 | 49.16 | 7 | 305.39 | 105.84 | 0.19 | 1 | 52 | 3 | 3.92 | 0.00% | 47.00% | 0.65 | 0.49 | 114.78 | 4 | 1.9 |
| 423 | 2017 | 1377 | 50.94 | 6 | 318.99 | 101.64 | 0.22 | 1 | 52 | 3 | 4.02 | 0.00% | 61.00% | 0.64 | 0.69 | 106.51 | 3 | 0.43 |
| 424 | 2017 | 764 | 52.52 | 7 | 302.36 | 1081.08 | 0.18 | 0 | 35 | 3 | 35.7 | 0.00% | 66.00% | 0.27 | 0.23 | 108.14 | 2 | 0.89 |
| 425 | 2017 | 1391 | 48.53 | 6 | 317.75 | 1085.04 | 0.19 | 0 | 37 | 2 | 35.72 | 0.00% | 69.00% | 0.23 | 0.21 | 106.71 | 1 | 2.62 |
| 426 | 2017 | 700 | 52.07 | 6 | 306.91 | 1095.27 | 0.19 | 0 | 35 | 2 | 35.64 | 0.00% | 56.00% | 0.64 | 0.64 | 114.10 | 2 | 1.36 |
| 427 | 2017 | 1131 | 52.36 | 7 | 317.55 | 1080.47 | 0.16 | 0 | 36 | 5 | 35.74 | 0.00% | 63.00% | 0.99 | 0.97 | 112.82 | 2 | 1.91 |
| 428 | 2017 | 1014 | 48.3 | 5 | 305.13 | 94.66 | 0.22 | 1 | 52 | 3 | 3.99 | 0.00% | 41.00% | 0.58 | 0.72 | 108.34 | 4 | 3.66 |
| 429 | 2017 | 729 | 52.42 | 6 | 317.12 | 1106.7 | 0.19 | 0 | 34 | 3 | 35.73 | 0.00% | 71.00% | 1 | 0.98 | 108.34 | 3 | 2.54 |
| 430 | 2017 | 1195 | 39.01 | 4 | 246.22 | 147.3 | 0.19 | 0 | 60 | 3 | 4.8 | 0.00% | 93.00% | 1.32 | 0.73 | 115.53 | 2 | 2.7 |
| 431 | 2017 | 1150 | 36.62 | 2 | 239.19 | 454.93 | 0.2 | 0 | 52 | 2 | 9.2 | 0.00% | 100.00% | 0.24 | 0.28 | 109.15 | 2 | 4.85 |
| 432 | 2017 | 789 | 38.21 | 3 | 240.95 | 481.04 | 0.17 | 0 | 51 | 2 | 9.32 | 0.00% | 100.00% | 0.25 | 0.27 | 111.12 | 2 | 4.04 |
| 433 | 2017 | 1062 | 40.87 | 2 | 241.93 | 144.69 | 0.25 | 1 | 61 | 5 | 4.74 | 0.00% | 100.00% | 0.44 | 0.55 | 116.61 | 6 | 7.34 |
| 434 | 2017 | 1590 | 39.4 | 3 | 233.36 | 464.13 | 0.2 | 0 | 52 | 3 | 9.24 | 0.00% | 100.00% | 0.27 | 0.24 | 112.75 | 3 | 2.97 |
| 435 | 2017 | 905 | 37.65 | 3 | 244.63 | 181.68 | 0.22 | 0 | 58 | 3 | 4.82 | 0.00% | 80.00% | 0.91 | 0.8 | 110.51 | 3 | 3 |
| 436 | 2017 | 889 | 38.2 | 3 | 247.05 | 474.75 | 0.21 | 0 | 51 | 5 | 9.33 | 0.00% | 100.00% | 0.24 | 0.26 | 115.87 | 3 | 4.49 |
| 437 | 2017 | 1011 | 37.66 | 3 | 238.3 | 475.07 | 0.18 | 0 | 52 | 4 | 9.3 | 0.00% | 100.00% | 0.25 | 0.25 | 110.85 | 3 | 1.76 |
| 438 | 2017 | 1374 | 24.84 | 2 | 280.64 | 773.17 | 0.21 | 0 | 56 | 3 | 11.73 | 0.00% | 85.00% | 0.59 | 0.47 | 111.73 | 3 | 4.51 |
| 439 | 2017 | 1771 | 26.16 | 2 | 276.64 | 537.24 | 0.23 | 0 | 44 | 5 | 1.61 | 22.00% | 52.00% | 0.91 | 0.48 | 110.98 | 3 | 5.05 |
| 440 | 2017 | 952 | 25.85 | 2 | 276.49 | 599.81 | 0.21 | 1 | 45 | 2 | 8.7 | 0.00% | 54.00% | 1.03 | 0.37 | 105.83 | 2 | 5.99 |
| 441 | 2017 | 1680 | 26.42 | 2 | 267.28 | 536.7 | 0.25 | 0 | 43 | 3 | 1.59 | 21.00% | 100.00% | 0.93 | 0.33 | 110.17 | 2 | 3.49 |
| 442 | 2017 | 1923 | 28.14 | 2 | 264.43 | 676.38 | 0.21 | 0 | 39 | 4 | 2.09 | 0.00% | 100.00% | 0.27 | 0.21 | 115.80 | 3 | 4.36 |
| 443 | 2017 | 1976 | 28.64 | 1 | 275.01 | 660.65 | 0.25 | 0 | 37 | 3 | 2.14 | 0.00% | 100.00% | 0.24 | 0.3 | 114.38 | 4 | 3.52 |
| 444 | 2017 | 1960 | 24.99 | 3 | 283.04 | 591.71 | 0.19 | 0 | 45 | 2 | 8.8 | 0.00% | 77.00% | 1.01 | 0.94 | 115.46 | 3 | 3.74 |
| 445 | 2017 | 2257 | 25.49 | 2 | 273.25 | 470.14 | 0.21 | 0 | 46 | 3 | 7.07 | 0.00% | 87.00% | 0.28 | 0.27 | 114.65 | 3 | 5.28 |
| 446 | 2017 | 888 | 26.44 | 2 | 269.92 | 521.36 | 0.2 | 0 | 41 | 4 | 1.71 | 19.00% | 100.00% | 0.98 | 0.47 | 114.04 | 3 | 2.14 |
| 447 | 2017 | 2228 | 28.5 | 2 | 263.65 | 536.05 | 0.22 | 0 | 44 | 2 | 1.65 | 22.00% | 48.00% | 0.96 | 0.38 | 113.56 | 3 | 7.07 |
| 448 | 2017 | 1229 | 28.08 | 2 | 265.2 | 642.22 | 0.24 | 0 | 38 | 2 | 2.2 | 0.00% | 75.00% | 0.23 | 0.3 | 107.86 | 3 | 2.84 |
| 449 | 2017 | 1288 | 28.83 | 3 | 280.93 | 503.69 | 0.25 | 1 | 45 | 2 | 1.61 | 24.00% | 100.00% | 1.1 | 0.47 | 111.53 | 4 | 6.45 |
