# Supplementary material for: Does the grassland ecological compensation policy improve the herders’ breeding technical efficiency in China?—Based on the parallel mediation effect model
Source: PLoS One. 2021 Apr 29;16(4):e0249990. doi: 10.1371/journal.pone.0249990 (PMC8084240; doi:10.1371/journal.pone.0249990)
Supplement: Attachment — Submitted filename: PLOS ONE REVIEW.docx [file pone.0249990.s006.docx]

**Does the Grassland Ecological Compensation Policy improve the herders' breeding technical efficiency in China?—Based on the parallel mediation effect model**

I congratulate the authors for spending their time to research into an emerging field. The current paper needs much work to be done.

Abstract does not reflect the main purpose of the study and major findings. The originality is not properly written. In abstract, the authors should present what the conclusions serve for. Clearly demonstrate the objective of this study by adding a paragraph at the end of introduction section.

The introduction should flow from the global understanding or argument around your topic or key variable. Why the study need to be done? What is significance of the research? What is the motivation for this study? What are the specific objectives of the study? What is the potential contribution of this study? None of them are reported in the introduction section. Many mistakes and unclear sentences also appear quite often in the texts, a native English speaker would be helpful in improving the quality of language.

There is no literature review section. At least, the authors could define the research variable and report what had been done by other researchers within their research setting. The author should also demonstrate any research gap in the literature review.

Your methodology is poorly explained and confusing. What is your research paradigm? Why descriptive design? If you are looking at identified factors that influence recreational cost of park visitors, then why descriptive design? Reconsider the research design. Where do you adopt your survey?

The conclusion did not capture the summary of each of the objectives. What new have contributed to existing literature? Where are the recommendations in this paper? The study did not show any of the following implications: theoretical, managerial and practical implications.
