# Supplementary material for: Does the grassland ecological compensation policy improve the herders’ breeding technical efficiency in China?—Based on the parallel mediation effect model
Source: PLoS One. 2021 Apr 29;16(4):e0249990. doi: 10.1371/journal.pone.0249990 (PMC8084240; doi:10.1371/journal.pone.0249990)
Supplement: Attachment — Submitted filename: Response to Reviewers.docx [file pone.0249990.s007.docx]

**Responses to the Reviewers’ Suggestions**

Many thanks to the PLOS ONE editor office for sending us the reviewers’ suggestions. We have discussed each suggestion from the reviewers seriously and made corresponding revision, and the following are our responses.

**Reviewer #1:**

(1) General comments
    -The font type should be checked,the font type of the title should be the same with other parts of the manuscript

-Set your computer to English language to avoid mixing up of Chinese and English font type. Example,the commas used in the lines 311,312,and 316, together with the character used in line 527 are in Chinese format,Please consider to change them to English format.

**Response:** Thank you for this suggestion. We have carefully checked the fonts and ensured that they are consistent.

-The whole manuscript text should be double-spaced when submitting
-Indicate the number on headings and subheadings(example. 1.Introductions,2.Materials and Methods,2.1.Description of the study area etc.
-Indicate the continuous page numbers on the manuscript
**Response:** Thank you for this suggestion. We have made corresponding amendments as required, including double-spaced, numbering on headings and subheadings, and indicate the continuous page numbers on the manuscript.

-The language quality score is 48/60, which is good,but try to recheck for minor improvements.The minor issues to check are; punctuation,article usage,prepositions,singular-plural form,subject-verb agreement,verb form, and avoid unnecessary word capitalization
-Consider replacing the word "herdsmen" with "herders" if applicable in the given context in the whole manuscript(consider uniformity when using the words)
-Consider replacing the word "paper" with "study" if applicable in the given context, or use the preferred word whether the study,paper, or article on the whole manuscript(these words been used in the lines 45,178,218,226,,292,322,3345, and 353)

**Response:** Thank you for this suggestion. We re-checked the spelling and grammar of the words in the manuscript, and unified the related expressions. We use "study" instead of "paper" and "herders" instead of "herdmens".We also have invited a professional editing group to improve the language.

-Indicate the Keywords below the abstract, minimum of 5 and maximum of 10 key words
-The funding statement section should appear after the conclusion section

**Response:** Thank you for this suggestion. We added five keywords(Grassland Ecological Compensation and Award Policy;Parallel Mediation Effect Model;Data Envelopment Analysis(DEA);Breeding Efficiency;Inner Mongolia) below the abstract and added the funding statement contents after the conclusion.

-Ethics statement should appear in the materials and methods section.In the ethics statement,PLOS ONE requires that,for studies reporting research involving human participants,the author(s) should confirm that the specific study was reviewed and approved by an institutional review board (ethics committee) before the study began. Please provide the specific name of the ethics committee/IRB that approved your study, or explain why you did not seek approval in this case.Also,please provide additional details regarding participant consents before data collection.

**Response:** Thank you for reminding. This herders’ survey was approved and carried out by the School of Economics and Management of Beijing Forestry University and the School of Finance and Economics of Jiangsu University, and was assisted by the local government. The main source of funding was the National Natural Science Foundation of China (No. 71704067). All herdsmen surveyed are aware of the purpose of the survey data. We have added corresponding instructions in the materials and methods section.

(2) Abstract section

-In the abstract section,please indicate briefly the specific study area(s),sampling methods/technique of the respondents and the sample size, data sources/types (primary or secondary data)and data collection method(whether by interview,questionnaire(cross-sectional or panel data). Also indicate the significance effect levels(eg. at P<0.05(5% etc) for results indicated in the abstract.Meaning that, you should consider to summarize the most important results and their significance levels.
**Response:** Thanks for your suggestions. We have rewritten the summary section, which introduces the source of the data and how it was collected, while supplementing the most important results and their significance levels. The specific description of the survey data will be described in more detail in the data source section(4.1Description of the study area).

(3) Introduction section
-The whole first paragraph in the introduction is not cited,please consider to cite it.
-Again, consider to cite(indicate the sources) the words from line 75 to line 81,from line 84 to line 87 , and from line 98 to line 119 . Please,consider to indicate the sources of all words or sentences which are not yours
**Response:** Thanks for your suggestions. We have combined the opinions of multiple reviewers and rewritten the original introduction part. The new content will be divided into two parts: introduction and literature review. Regarding the citation problem you mentioned, we have revised it in the new content and guaranteed to indicate the sources of all words or sentences which are not ours.

(4) .Materials and Methods section

-Please recheck the model formula,you have indicated that i(i=1,...,m) is the inputs and r(r=1,...,s) is the output,but they do not appear in the formula,consider to recheck it or elaborate more the model formula in detail.

**Response:** Thanks for your suggestions. Sorry, there was an error in our statement,Where $i(i=1,\ldots\text{，m})$is the subscript number of input, $r(r=1,\ldots\text{，s})$ is the subscript number of output, the actual input items and output items are represented by x and y.We have modified this part of the content.

-Restructure(regroup) the materials and methods section to indicate clearly the;

-(i) Description of the specific study area/site.Consider to put a map if possible

-(ii) Sampling procedures of the participants(respondents) together with sample size if applicable

-(iii) Data collection methods and data sources if applicable

-(iv) Analytical models/data analysis models

-(v) Conceptual framework of the study

-(vi) Variables selection and justification supported by literature if available.You can also include the hypothesized effect of variables on bleeding efficiency(optional)

In addition,clarify in this section why you intend to publish 2018 surveyed data in 2020? and not earlier?say 2019
**Response:** Thanks for your suggestions. Combined with the comments of reviewer 3, we rewrite the Data and variables section, which adds a detailed description of the survey visit area.

-(i) (ii) (iii) :The data used in this study comes from a field questionnaire interview on herders in the pastoral area of Inner Mongolia conducted by the research team. from June to November 2018. First, based on the regional characteristics of Inner Mongolia’s grasslands and pastoral areas, the Xinbarag Right(Hulunbeier City), Chenbarag Qi (Hulunbeier City), West Ujimqin (Xilinguole League) and Siziwang (Ulanchabu) were selected as the geographical distribution of the 4 sample counties is shown in the figure1. Then, taking into account the differences in population and grassland area, 4 to 8 sample towns (Sumu) were selected in each sample county, and a questionnaire survey was conducted with herders in the form of "one-to-one" interviews. The questionnaire survey includes the following aspects: basic characteristics of herders households (sex, age, education level, grassland area, etc.); basic household conditions (total population, household income level and structure, etc.); livestock production investment, Livestock breeding expenses, livestock income, living expenses, grassland compensation policy and other subsidies. A total of 449 valid questionnaires were collected in this survey.

Figure 1 Locations of the study area

-(iv) (v) :We have provided a research framework diagram (Fig. 2) in the new submission. Fig.2 briefly and clearly shows the motivation, data sources, Mechanism analysis and modeling steps, Model results and discussion, conclusions of our work.

-In addition,the main reason we did not publish 2018 surveyed data in 2019 is that we did not use this set of data in time. GECP's research on herder farming efficiency is the result of changing herders' breeding behavior. Before this study, we paid more attention to the changes of herdsmen's breeding behavior before and after the implementation of the policy.

Figure 2 The research framework diagram.

-(vi): We rewrote the methods and data parts, and gave a detailed introduction to the selected methods and selected variables.

(5) Results and discussion section

-When reporting and discussing the results ,please indicate the significance effect levels of every variables included in your model (eg. X has a positive impact on Y and statistically significant at P<0.05(5%),0.01(10%), etc or otherwise)

-State what other similar studies in other countries have reported(if available)
**Response:** Thank you for your suggestion. We have marked the significance level of each variable in the results section of the resubmitted manuscript. Because GECP is implemented in China, although there are similar ecological compensation policies in the world, the implementation process and reward methods are quite different from those of GECP and are not comparable. At the same time, the related research rarely involves the impact on the breeding efficiency of herdsmen. We have selected two articles by Chinese scholars to support our conclusion (Liu et al. (2019) and Han et al. (2019)).

(5) Referencing and citation:
-There are wrong citations in the lines 155,159,164,165,172,173,220,316,318,333, and 488,consider to recheck,please citations should be by numbers and not by year of publication,example[10] and not (Zhu Ning, 2016)
-References number 3,4,5,15,16,17,19,21,22,23,24,30,and 31 are in the reference list but not cited in the manuscript,consider to remove or cite them.In addition reference number 40 and 42 are been cited in line 454 but are not listed in the reference list,consider to add them. For more details visit the citation and referencing guidelines on the PLOS ONE homepage
-You are encouraged to use citation and referencing tools such as End Note to avoid errors.
**Response:** Thank you for your suggestion. We carefully refer to the citations and reference guide on the PLOS ONE homepage and revise the citations of the manuscript with the help of ENDnote.

**Reviewer #2:**

The authors provide first-hand survey data, hoping to prove the research conclusion. The authors have provided data on all the findings in their manuscripts.Two suggestions for amendment.

1. Supplement the latest academic literature related to this paper in 2020.

**Response:** Thanks for your suggestions. We have supplemented the latest relevant academic literature in 2020 as much as possible in the article.

2. Add discussion on the research process and conclusion of the paper.

**Respons**e: Thanks for your suggestions. We have added a description of the research process of the article to the newly submitted manuscript. First, we used the DEA method to measure the efficiency of herder farming in 2010 and 2017, and then used the package in R: "lavan" and "Mediation" calculates the mediation effect model, in which farming scale, farming structure and pasture circulation are used as mediating variables. At the same time, we divided herders into large-scale and small-scale, and discussed the heterogeneity of the impact of GECP on herders' breeding efficiency.

**Reviewer #3:**  I congratulate the authors for spending their time to research into an emerging field. The current paper needs much work to be done.
Abstract does not reflect the main purpose of the study and major findings. The originality is not properly written. In abstract, the authors should present what the conclusions serve for.

**Response:** Thanks for this suggestion. We have rewritten the abstract in the new submission. In the new abstract, we elaborated on the motivation, method, result of this article. The main purpose of this research is to answer whether the implementation of the GECP policy can improve the grassland The breeding efficiency of herders, and the effect path of scale changes, grassland circulation behavior and changes in breeding structure are analyzed. The main conclusion drawn is that the total effect of the grassland ecological compensation policy on the breeding efficiency of Inner Mongolia grassland herdsmen is a positive effect (P<0.01), and the change of breeding methods (direct effect) is the main influence path, and the grassland circulation behavior (P<0.01) and the scale of breeding (P<0.01) are part of the mediating effect, and the mediating effect of the breeding structure is not significant (P>0.1).

Clearly demonstrate the objective of this study by adding a paragraph at the end of introduction section.The introduction should flow from the global understanding or argument around your topic or key variable. Why the study need to be done? What is significance of the research? What is the motivation for this study? What are the specific objectives of the study? What is the potential contribution of this study? None of them are reported in the introduction section. Many mistakes and unclear sentences also appear quite often in the texts, a native English speaker would be helpful in improving the quality of language.

**Response:** Thanks for this suggestion. We have rewritten the introduction part based on the opinions of you and reviewer 1, which describes the research purpose and significance of this article.The main purpose of this research is to answer whether the implementation of the GECP policy can improve the grassland The breeding efficiency of herders, and the effect path of scale changes, grassland circulation behavior and changes in breeding structure are analyzed. The main conclusion drawn is that the total effect of the grassland ecological compensation policy on the breeding efficiency of Inner Mongolia grassland herdsmen is a positive effect (P<0.01), and the change of breeding methods (direct effect) is the main influence path, and the grassland circulation behavior (P<0.01) and the scale of breeding (P<0.01) are part of the mediating effect, and the mediating effect of the breeding structure is not significant (P>0.1).

In the literature review section, we propose the marginal contribution of this research.This study makes the following contributions on the basis of previous studies: 1) enrich the research of GECP on livestock breeding efficiency, and study the impact of policies on livestock breeding efficiency from a micro perspective based on the survey data of Inner Mongolia Pastoral Areas; 2) establish a quantitative model to analyze the impact of GECP on the technical efficiency of herders’ breeding, because GECP changes the efficiency by influencing herders’ breeding behavior. Therefore, the parallel mediating effect model was utilized to analyze the influence mechanism of GECP on aquaculture efficiency from three action paths: breeding scale, breeding structure and grassland circulation.

There is no literature review section. At least, the authors could define the research variable and report what had been done by other researchers within their research setting. The author should also demonstrate any research gap in the literature review.

**Response:** Thanks for this suggestion. We have combined the opinions of multiple reviewers and rewritten the original introduction part. The new content will be divided into two parts: introduction and literature review. Reviewing the previous literature on the implementation effect of grassland ecological compensation policy, it can be summarized into two aspects. On the one hand, the impact of GECP on grassland ecological environment, that is, ecological effect, mainly focuses on the changes of the thickness of the grass layer of natural grassland, grassland coverage and biomass before and after the implementation of GECP. Based on the perspective of herders’ animal husbandry production, another part of the works studies the impact of GECP, that is, economic effect. However, there are two shortcomings in this kind of studies: firstly, the data adopted is open and macro; secondly, this kind of studies only compare the breeding efficiency prior to and after the implementation of GECP, but do not conduct the precise estimate through metering model or do not analyze the effecting mechanisms.

Therefore, this study makes the following contributions on the basis of previous studies: 1) enrich the research of GECP on livestock breeding efficiency, and study the impact of policies on livestock breeding efficiency from a micro perspective based on the survey data of Inner Mongolia Pastoral Areas; 2) establish a quantitative model to analyze the impact of GECP on the technical efficiency of herders’ breeding, because GECP changes the efficiency by influencing herders’ breeding behavior. Therefore, the parallel mediating effect model was utilized to analyze the influence mechanism of GECP on aquaculture efficiency from three action paths: breeding scale, breeding structure and grassland circulation.

Your methodology is poorly explained and confusing. What is your research paradigm? Why descriptive design? If you are looking at identified factors that influence recreational cost of park visitors, then why descriptive design? Reconsider the research design.

**Response:** Thanks for this suggestion. We have provided a research framework diagram (Fig. 2) in the new submission. Fig.2 briefly and clearly shows the motivation, data sources, Mechanism analysis and modeling steps, Model results and discussion, conclusions & Suggestions of our work. I'm sorry that I don't understand the "entertainment cost affecting park visitors". If you are not satisfied with our modification, we welcome your suggestions for further modification. Thank you very much.

Figure 3 The research framework diagram.

Where do you adopt your survey?

**Response:** The data used in this study comes from a field questionnaire interview on herders in the pastoral area of Inner Mongolia conducted by the research team. from June to November 2018. First, based on the regional characteristics of Inner Mongolia’s grasslands and pastoral areas, the Xinbarag Right(Hulunbeier City), Chenbarag Qi (Hulunbeier City), West Ujimqin (Xilinguole League) and Siziwang (Ulanchabu) were selected as the geographical distribution of the 4 sample counties is shown in the figure. Then, taking into account the differences in population and grassland area, 4 to 8 sample towns (Sumu) were selected in each sample county, and a questionnaire survey was conducted with herders in the form of "one-to-one" interviews. The questionnaire survey includes the following aspects: basic characteristics of herders households (sex, age, education level, grassland area, etc.); basic household conditions (total population, household income level and structure, etc.); livestock production investment, Livestock breeding expenses, livestock income, living expenses, grassland compensation policy and other subsidies. A total of 449 valid questionnaires were collected in this survey.

The conclusion did not capture the summary of each of the objectives. What new have contributed to existing literature? Where are the recommendations in this paper? The study did not show any of the following implications: theoretical, managerial and practical implications.

**Response:** Thank you for your suggestion. We have rewritten the introduction, literature review and conclusion in the newly submitted manuscript. In the Literature review section we pointed out the contribution of this research：1) enrich the research of GECP on livestock breeding efficiency, and study the impact of policies on livestock breeding efficiency from a micro perspective based on the survey data of Inner Mongolia Pastoral Areas; 2) establish a quantitative model to analyze the impact of GECP on the technical efficiency of herders’ breeding, because GECP needs to influence herders’ breeding behavior. At the end of this article, we rewrote the policy recommendations part, combined with the research conclusions of this article, we put forward three recommendations: 1)While continuing to implement the current grassland ecological protection compensation policy, Chinese government should formulate corresponding supporting policies to support the transformation and development of livestock husbandry in pastoral areas through technology and funds, such as the policy support for the construction of breeding land and for transforming beef cattle grazing to house breeding, forming a joint force with the ecological compensation policy to promote the transformation ;2)the government should actively establish corresponding grassland circulation platform to avoid grassland waste and improve resource utilization efficiency;3) improving the breeding level of herders by means of technical training is helpful to further enhance the economic benefits of livestock husbandry, and will contribute to the professional and modern development of animal husbandry

**Reviewer #4:**

1. Generally speaking, mediator should remain independent. However, in the model construction of the influence path study, scale is used as an mediator, and at the same time, the production efficiency calculated by the scale is used as the outcome variable, so that the independence of the mediator variable does not exist, and the result will be unstable.

**Response:** Thank you very much for your valuable suggestion which we have not considered. Next, I will elaborate on the following three points: First, due to our misrepresentation, the output variable that we estimate the efficiency of breeding is: the Total outcome Livestock number (standard sheep), this variable is not consistent with scale (farming scale) , According to our understanding the Total Livestock number (standard sheep)=scale *the ratio of outgoing stocks, but we agree with your statement "the production efficiency calculated by the scale is used as the outcome variable", because Y and scale have a strong correlation; Second, in the study, we conducted a discussion on the heterogeneity of herders of different sizes, and divided herders into large-scale herders and small-scale herders according to the median of the sample size in 2011, and established mediation effects respectively. The model is estimated. From the results of the model, the two types of herdsmen are not affected by the GECP policy. Therefore, we believe that our results are robust. Finally, in order to further ensure the accuracy of the results, we have The output variables were standardized. The output variables were divided by the slaughter amount to 1, and the input variables were changed to the area, lab, feed, and other required to produce 1 unit of standard sheep. The efficiency was re-estimated (average breeding in 2010). The efficiency was 0.2760, and the average breeding efficiency in 2017 was 0.2911), and the mediation effect model was established to obtain the following results: It can be found that the sign and significance of the core explanatory variables (policy) in each equation has not changed significantly. The final mediating effect path analysis can find that the direct effect is positive and accounted for 76.17%, and the indirect effect is positive and accounted for 23.86%, which is not much different from the original results (ADE, 82.22%; ACME, 17.78%), so it is believed that eliminating the impact of scale did not cause a major change in the results.

Table 1 mediation model results (calculation efficiency after data standardization)

|  | Dependent variable: | | | |
| --- | --- | --- | --- | --- |
|  | Larea | scale | farmstr | eff |
| scale |  |  |  | 0.051*** |
|  |  |  |  | (-0.008) |
| farmstr |  |  |  | 0.003 |
|  |  |  |  | (-0.029) |
| Larea |  |  |  | 0.048*** |
|  |  |  |  | (-0.012) |
| policy | 6.584*** | 8.893*** | 0.512 | 2.442*** |
|  | (-0.825) | (-1.211) | (-0.34) | （-0.317） |
| inc | 0.001 | -0.001 | 0.002*** | -0.002*** |
|  | (-0.001) | (-0.002) | (-0.001) | （-0.0004） |
| incstr | 0.161 | 0.29 | -0.011 | -0.064 |
|  | (-0.172) | (-0.252) | (-0.071) | （-0.062） |
| price | -0.001 | 0.002 | 0.001 | -0.002** |
|  | (-0.004) | (-0.006) | (-0.002) | （-0.001） |
| age | 0.004*** | -0.001 | 0.0002 | 0.001*** |
|  | (-0.001) | (-0.002) | (-0.001) | （-0.0005） |
| edu | -0.034** | -0.008 | -0.003 | -0.003 |
|  | (-0.015) | (-0.022) | (-0.006) | （-0.005） |
| lab | 0.022* | -0.005 | -0.012*** | 0.005 |
|  | (-0.011) | (-0.017) | (-0.005) | （-0.004） |
| dis1 | 0.125** | -0.046 | -0.035* | 0.006 |
|  | (-0.05) | (-0.073) | (-0.02) | （-0.018） |
| dis2 | -0.065 | （-0.031） | -0.139*** | 0.018 |
|  | （-0.066） | （-0.097） | -0.027 | （-0.024） |
| Constant | 309.834*** | 444.257*** | 23.976 | 129.121*** |
|  | （-44.99） | （-65.987） | （-18.516） | （-17.032） |
| Year | Yes | Yes | Yes | Yes |
| Firm | Yes | Yes | Yes | Yes |
| R2 | 0.141 | 0.634 | 0.1 | 0.621 |
| F Statistic | 13.170*** | 139.598*** | 8.989*** | 103.497*** |

*** p<0.01, ** p<0.05, * p<0.1

|  | Theoretical path | coefficients | Path coefficients | Total path coefficient  (effect ratio) |
| --- | --- | --- | --- | --- |
| ADE  （Direct effect） |  | $d$ | 2.442***  (0.312) | 2.442***  （76.17%） |
| ACME  (Indirect effect) | Policy→Structure→EFF | $d*n$ | 0.002  (0.015) | 0.765***  （23.86%） |
|  |  |  |  |  |
|  | Policy→Scale→EFF | $b*f$ | 0.449***  (0.088) |  |
|  |  |  |  |  |
|  | Policy→Circulation→  （ADE）/Scale(ACME)→EFF | $c*g$ | 0.314***  (0.094) |  |
|  |  |  |  |  |
| ATE  （Total effect） |  |  | 4.561***  (0.303) | 3.206***  （100%） |

*** p<0.01, ** p<0.05, * p<0.1

Based on the above three points, we believe that the results of this study are robust and credible. Thank you again for pointing out the points that we have not noticed. If you are not satisfied with our modification or have other question, we welcome your suggestions for further modification. Thank you very much.

2. In the influence path study, the time trend is not stripped. That is, in 2010-2017, there will be various exogenous variables, rather than the factors of the policy itself, which will affect the production efficiency of farmers, leading to the effect of the policy being magnified.

**Response:** Thank you for your suggestion. We very much agree with your opinion. Before the start of this study, we tried to consider using the PSM-DID model to eliminate common trends in time. However, since the GECP policy covers almost all herder families, there is almost no control Group (ie the control group not affected by GECP) for comparison; However, this study refers to related research trying to control the influence of GECP and other factors by using a fixed-effects model, which can Control most of the time-varying variables, including livestock prices, household factors and climate factors, to obtain an accurate estimate of GECP's breeding efficiency for herders.

3. In Table 1, what are the specific reasons for the positive impact of grassland policy on the scale of household animal husbandry? Is there such a positive effect? Please specify. Does this positive effect really exist? Please be specific.

**Response:** Thank you for your suggestion. In response to your questions, I will answer them one by one: I think that the GECP policy has a positive effect on the increase of herd farming scale. We quote macro data to prove this point. Table 3 records the Average number of rearing per household, It can be found that this value has increased. The specific reasons will be explained from the following two aspects:

Table 2 Average number of rearing per household from 2011 to 2017

| Year | 2011 | 2012 | 2013 | 2014 | 2015 | 2016 | 2017 |
| --- | --- | --- | --- | --- | --- | --- | --- |
| Number of herders households in Inner Mongolia pastoral area (ten households) | 48.87 | 48.45 | 55.50 | 57.28 | 57.33 | 57.67 | 56.11 |
| Number of sheep and goat in Inner Mongolia pastoral area at the end of the year (ten thousand) | 5276.00 | 5144.00 | 5239.20 | 5569.28 | 5777.80 | 5506.24 | 6111.93 |
| Average number of rearing per household | 107.96 | 106.17 | 94.40 | 97.23 | 100.79 | 95.48 | 108.92 |

1：The purpose of the GECP policy itself is not to reduce the scale of pastoral farming: the purpose of the GECP policy itself is to protect the grassland ecology in China and promote the transformation and upgrading of animal husbandry. It does not mean that the implementation of the policy is to reduce the scale of herdsmen’s breeding, and reducing the intensity of pasture utilization is not the same as reducing the scale of herders’ breeding, because herders can switch to shed-raising methods or rent out herdsmen who give up livestock production. Expansion of the pasture. We used two data to prove this statement: one is the value of fixed assets produced by herdsmen in Inner Mongolia at the end of the year (data source: "Inner Mongolia Statistical Yearbook") and the average amount of food consumed to raise a sheep (data source: "Compilation of National Agricultural Product Cost and Benefit Data"), it can be found that these two values have fluctuated and increased from 2011 to 2017. Therefore, we believe that herders are gradually changing traditional grazing and feeding methods, and the reduction in the intensity of pasture utilization does not mean the expansion of herdsmen’s farming scale.

|  | Average value of fixed assets produced by herdsmen at the end of the year($ in 2015) |
| --- | --- |
| 2011 | 4148.71 |
| 2012 | 4116.19 |
| 2013 | 5174.74 |
| 2014 | 6200.77 |
| 2015 | 6388.86 |
| 2016 | 6848.49 |
| 2017 | 6872.23 |

Figure 4 Average value of fixed assets produced by herdsmen at the end of the year($ in 2015)

Table 3 Quantity of food consumed by sheep from 2011 to 2017

|  | Year | unit | sheep | goat |
| --- | --- | --- | --- | --- |
| Quantity of food consumed per 100 | 2011 | kg | 816.66 | 2584.45 |
| Quantity of food consumed per 101 | 2012 | kg | 1004.27 | 2790.63 |
| Quantity of food consumed per 102 | 2013 | kg | 847.24 | 3616.24 |
| Quantity of food consumed per 103 | 2014 | kg | 1077.77 | 3867.56 |
| Quantity of food consumed per 104 | 2015 | kg | 1208.54 | 3401.42 |
| Quantity of food consumed per 105 | 2016 | kg | 1218.28 | 4197.59 |
| Quantity of food consumed per 106 | 2017 | kg | 1267.62 | 4077.75 |

2 . GECP's own subsidies and supervision are not very strong. As a rational person, herders have more economic rationality than ecological rationality. After receiving supplementary funds, they are more inclined to use funds for the expansion of production scale, which has led to grassland ecology to a certain extent. The compensation policy for environmental protection is ineffective; Secondly, the compensation for the grassland ecological compensation policy is low, and at the same time, the implementation of policy supervision is difficult, and illegal grazing often occurs(Hu Yuanning, 2019).

**Reviewer #5:**

This article discusses an interesting topic. The ecological compensation policy plays an important role in improving China's environment. This article discusses the impact of grassland ecological compensation policy on the efficiency of herders’breeding technical efficiency. And further, it focuses on the impact mechanism of the policy on the efficiency. The structure of article is reasonable and it can arouse readers' interest. I recommend publishing in its current form

**Response:** Thanks for your postive comments.

**Reviewer #6:**

Try to be more specific and clearer of variables measurement and explain the model employed. Please, kindly take time to read the instructions for authors and follow closely the guidelines of the journal. The paper contains a lot of English and grammatical errors. Kindly edit your English. Recheck the authors guide on references and citations and update your paper to fit the journal requirements.

**Response:** Thanks for your suggestions. We rewrote the methods and data parts, and gave a detailed introduction to the selected methods and selected variables. We followed the requirements of the journal to review the unreasonable places in the paper, and invited a professional English editing team to improve the language. If you are not satisfied with our modification or have other question, we welcome your suggestions for further modification. Thank you very much.
