# Supplementary material for: Does the grassland ecological compensation policy improve the herders’ breeding technical efficiency in China?—Based on the parallel mediation effect model
Source: PLoS One. 2021 Apr 29;16(4):e0249990. doi: 10.1371/journal.pone.0249990 (PMC8084240; doi:10.1371/journal.pone.0249990)
Supplement: Attachment — Submitted filename: Manuscript (4).docx [file pone.0249990.s008.docx]

Does the Grassland Ecological Compensation Policy improve the herders' breeding technical efficiency in China?

—Based on the parallel mediation effect model

Yun Wang^1, #a^,Yaqiong Han^2^,Yijun Han^1,#a^ , Wenchao Li^3*,#b^

1	College of Economics and Management, China Agricultural University, Beijing, China;

2	School of economics & management, Beijing Forestry University, Beijing, China;

3 School of Financial & Economics ,Jiangsu University,Zhenjiang,china;

#a Current Address: College of Economics and Management, China Agricultural University, Beijing, China

#b Current Address: School of Financial & Economics ,Jiangsu University,Zhenjiang,China

* Corresponding author:

E-mail: 68345954@ujs.edu.cn(WL)

**Funding:** This research was funded by National Natural Science Foundation of China (No. 71704067) and Humanity Science Foundation of Ministry of Education ( No. 17YJC790080)

# Abstract

The Grassland Ecological Compensation Policy (abbreviated as GECP), which aims to realize the ecological protection by reducing the stock-carrying capacity of pastures and promote the transformation of pasture animal husbandry by improving the herders’ breeding methods, has been a major project in China’s grassland pastoral areas and grassland ecological construction. GECP makes herders get financial subsidies while reducing the utilization of grassland, which brings challenges and opportunities to herders’ traditional livestock production. Based on the two-stage data of 449 herders obtained by stratified random sampling in Inner Mongolia grassland area of China in 2018, this study used data envelopment analysis (DEA) to measure the breeding efficiency of herders before and after the implementation of GECP, and then constructed a parallel mediating effect model from the three action paths of livestock breeding scale, grassland circulation behavior and breeding structure change to analyze the effect and the effecting path of the implementation of GECP on the efficiency of herders’ livestock breeding. The research results show that the general effect of GECP on the breeding efficiency of herders in inner Mongolia is positive (P<0.01), and the change of breeding methods (direct effect) is the main influence path. Specifically, the grassland circulation behavior (P<0.01), the scale of breeding (P<0.01) is part of the mediating effect, and the mediating effect of the breeding structure is not significant (P>0.1). This study also shows that the non-agricultural and animal husbandry income of herders has a negative impact on the breeding efficiency (P<0.01), and herders’ age and breeding scale have a positive effect on the breeding efficiency (P<0.01). This research has not only answered the question whether the GECP can improve the efficiency of husbandry, but also focused on the analysis of the impacting mechanism of policies on efficiency. It is of great significance to further improve GECP and the related supporting policies and promote the transformation of China’s grassland animal husbandry.

**Keyword**

Grassland Ecological Compensation and Award Policy (GECP); Parallel Mediation Effect Model; Data Envelopment Analysis(DEA);Breeding Efficiency; Inner Mongolia

# 1. Introduction

In recent decades, influenced by the natural factors such as climate change and the human factors such as overloading and overgrazing, the ecological environment of grassland has been deteriorating in china [ ] The area of natural grassland in northern China is about 313 million hectares, accounting for 79.7% of the national natural grassland area, which is of great significance to the economic development of pastoral areas and even the whole country. However, from 1988 to 2008, the grassland coverage had been declined by about 2.3 million hectares[1]. On the one hand, most grassland in China locates in arid and semi-arid areas where the natural environment is relatively bad []. Extreme climatic conditions such as drought, sandstorm and snow disaster occur frequently. At the same time, the precipitation is small, generally less than 300 mm/year, and the annual and spatial distribution is uneven, resulting in serious degradation of grassland ecological function in northern China. On the other hand, the extensive development of grassland animal husbandry in the early stage only focused on the development and utilization of grassland, but did not pay attention to the technical efficiency of breeding. The extensive development mode of high consumption and low output has degraded seriously the grassland ecological environment[2]. About 90% of the available grassland in China have been degraded to varying degrees, and the grassland ecological service function in some areas has been weakening, thus affecting the sustainable development of pastoral areas and even the ecological security of our country. Meanwhile, the grassland yield has gradually decreased, which affects livestock production and threatens the production function and livelihood of the grassland[3-5].

Given the worsening trend of grassland ecology in northern China, the Chinese government has taken a series of measures to protect the ecology of northern pastoral areas and promote the sustainable development of grassland. In 2003, China started the project of returning grazing land to grassland. In June 2011, the State Council of China issued Several Opinions on Promoting the Sound and Rapid Development of Pastoral Areas[6]. To implement GECP in eight major grassland and pastoral areas, Including Inner Mongolia, Sichuan, Yunnan, Tibet, Gansu, Ningxia, Qinghai, Xinjiang and establish a grassland ecological protection subsidy incentive mechanism from "single compensatory subsidy" to "coexistence of compensatory and incentive subsidies" (hereinafter referred to as Grassland Ecological Compensation Mechanism). By 2012, the scope of policy implementation has been expanded to cover 268 pastoral and semi pastoral areas and counties in China[7]. Grazing prohibition and grassland livestock balance system are two important contents of GECP, which limit the herders’ free grazing behavior. Grazing prohibition refers to measures that prohibit the use of grazing on natural grasslands within a certain period of time. The period is generally more than one year, during which no transfer of grazing is allowed.Grass-livestock balance refers to the limitation of grassland development and utilization based on the carrying capacity of grasslands outside the grazing prohibition area. The two methods have different restrictions on herders' grazing behavior, and there are also differences in the amount of rewards. The grazing prohibition subsidy and the forage-livestock balance reward standard are 30.46 USD/ha and 10.13 USD/ha, respectively.

Since then, the long-term implementation of this policy has brought opportunities and challenges to herders' livestock breeding.Herders need to reduce the number of livestock grazing on the grassland, or increase the purchase of forage and carry out house feeding for livestock. These measures brought great challenges to the livestock breeding in grassland pastoral areas, but also give some opportunities for herders’ animal husbandry in terms of grassland grazing prohibition subsidy, grass livestock balance reward and performance appraisal reward. Considering both the opportunities and the challenges, the improvement of breeding efficiency becomes the main content of this study. The improvement of herders’ breeding efficiency is also the key to promoting the transformation and upgrading of livestock husbandry. Therefore, this study is of great significance to the further improvement of GECP, the related supporting policies as well as the promotion of the transformation of China’s grassland livestock husbandry.

This study is divided into five parts. After the introduction, Section 2 summarizes the relevant literature on the implementing effect of GECP. Section 3 and section 4 present the methods and data descriptive statistics.Section 5 and section6 summarize the results and discussions. Finally, Section 7 Summarizes the conclusions of this study and section8 puts forward some relevant policy recommendations.

**2.** **Literature review**

Reviewing the previous literature on the implementation effect of grassland ecological compensation policy, it can be summarized into two aspects. On the one hand, the impact of GECP on grassland ecological environment, that is, ecological effect, mainly focuses on the changes of the thickness of the grass layer of natural grassland, grassland coverage and biomass before and after the implementation of GECP, the results of researches differ due to the factors of different research areas and so on: Some studies have shown that under the influence of policies, grassland ecology has been generally improved, and the height, coverage and biomass of grassland vegetation have increased (Yang et al., 2016; Liu Aijun, 2014)[8, 9], but the effect of policies has been different in different regions (Liu et al, 2017)[10]. Based on the perspective of herders’ animal husbandry production, another part of the works studies the impact of GECP, that is, economic effect. As the direct users of grassland and the provider of ecological services, the impact of policy on livestock production mainly lies in its impact on livestock breeding behavior of herders, and the results of the relevant studies show: in terms of grassland grazing, some studies show that the implementation of GECP has effectively reduced the grazing time of herders and the number of livestock per unit grassland(Wang,2017;Hu, 2019)[11, 12]. However, some studies believe that the subsidy standard of policy is low and the compensation standard of the grass-livestock balance policy is also relatively low, and it is difficult to alleviate the livelihood pressure of small and medium-sized herders (Li, 2014)[13].Liu Chunpeng and Xiao Haifeng (2019) studied the production situation of cashmere sheep farmers in five provinces and regions and showed that the grassland grazing prohibition policy has a positive impact on the technical efficiency of farmers and herdsmen, and the impact effect is different due to the different breeding scale of farmers and herdsmen. And it is difficult to improve the breeding efficiency of small-scale herdsmen, that is, the impact of grazing prohibition policy on the production efficiency of herdsmen has a threshold effect[14].In addition, the improvement of herdsmen’s breeding efficiency also depends on factors such as the age of the head of herdsmen (Zhu Ning, 2016), the family labor force (Yang Xudong, 2018; Li Jie, 2019), regional environment (Wei Yanjiao, 2019) and other factors[15-18].

However, there are two shortcomings in this kind of studies: firstly, the data adopted is open and macro; secondly, this kind of studies only compare the breeding efficiency prior to and after the implementation of GECP[19], but do not conduct the precise estimate through metering model or do not analyze the effecting mechanisms.Therefore, enrich the research of GECP on livestock breeding efficiency, and study the impact of policies on livestock breeding efficiency from a micro perspective based on the survey data of Inner Mongolia Pastoral Areas; 2) establish a quantitative model to analyze the impact of GECP on the technical efficiency of herders’ breeding, because GECP needs to influence herders’ breeding behavior. Therefore, the parallel mediating effect model was utilized to analyze the influence mechanism of GECP on aquaculture efficiency from three action paths: breeding scale, breeding structure and grassland circulation. The research framework is as follows (Fig. 1).

**Figure 1 The research framework diagram**

# 3. Methodology

## 3.1. Data Envelopment Analysis

Before analyzing the impact of GECP on breeding efficiency, it is necessary to measure it first. This study adopts the data envelopment analysis (DEA) method. The main advantage of this method is that it does not have strict requirements on the form of the model[20, 21]. DEA mainly sets the highest output frontier that can be achieved under a certain input level as the efficiency frontier, and measures the gap between the actual output level of the decision-making unit and the efficiency frontier as its relative efficiency value. The deviation is more concise and effective for the calculation of agricultural production efficiency. This study adopts the input-oriented data envelopment analysis model to convert the fractional programming with the best input and output efficiency into its equivalent linear and dual programming, introduce slack variables and non-Archimedean infinitesimals, and rewrite the inequality constraints after the equality constraints. And then the following measurement model is obtained:

$\begin{matrix} \min[\theta-\varepsilon(e_{1}^{T}s^{-}+e_{2}^{T}s^{+})] \\ s.t.\left\{\begin{matrix} \sum_{j=1}^{n} \lambda_{j}x_{ij}+s^{-}=\theta x_{i0} \\ \sum_{j=1}^{n} \lambda_{j}y_{rj}-s^{+}=y_{r0} \\ \lambda_{j}\geq0,s^{+}\geq0,s^{-}\geq0 \end{matrix}\right. \end{matrix}$ （1）

Where $i(i=1,\ldots\text{，m})$is the subscript number of input, $r(r=1,\ldots\text{，s})$ is the subscript number of output, $j(j=1,\ldots\text{，n})$is the subscript number of decision-making unit, $x_{ij}$ is the i-th input of the j-th DMU，$y_{rj}$ is the r-th output of the j-th decision-making unit, $\lambda_{j}$is the weight coefficient, $s^{-}$and $s^{+}$ are the input redundancy variables, $\theta$ represents the overall efficiency of the decision-making unit ，and$\varepsilon$ is non-Archimedean infinitesimal quantity.$e_{1}^{T}\text{=（1，}\ldots\text{，1）}\in\text{E}_{m}$，$e_{2}^{T}\text{=（1，}\ldots\text{，1）}\in\text{E}_{s}$，$\text{E}_{m}$, $\text{E}_{m}$are m-dimensional and s-dimensional unit vector spaces, respectively. When the effectiveness of the decision-making unit DMU 0 is analyzed, its input is $x_{i0}$, the output is $y_{r0}$, and the optimal solution can be obtained; when $\theta^{\text{*}}$=1, and or, DMU 0 is weak DEA valid; when，DMU 0 is DEA valid，and its economic activity has both technical and scale effectiveness.

## 3.2. Parallel Mediation Effect Model

The efficiency improvement of breeding technology will enable Herders to reduce their dependence on the natural environment in the process of animal husbandry and reduce the pressure of grazing on natural grasslands. This is an important way to improve the comprehensive strength of animal husbandry in pastoral areas. Based on this, the herd farming efficiency studied in this study refers specifically to the technical efficiency of herd farming. The impact mechanism of GECP on the efficiency of herders’ livestock breeding is complicated. Zhang et.al (2012) believes that “improved varieties + artificial supplementary feeding + shed overwintering + timely delivery” is the main way for herders to improve production levels[22], and GECP is mandatory. The new system and compensatory funds will transform the herders’ breeding behavior and enable herders to improve their production levels. In general, GECP has a profound impact on the objective conditions and subjective motivations of herders engaged in animal husbandry. Therefore, this study proposes the following path to analyze the mechanism of the impact of compensation policy on herders’ breeding efficiency:

- Path 1: GECP shortens the grazing time on the grassland for herders to raise livestock, and changes the ratio of herders’ house-feeding to directly affect the efficiency of breeding.Nomadic grazing and free grazing are traditional production methods in grassland pastoral areas.In Mongolia grassland there is the withered grass period from this November to May of the next year. Herders used traditional production methods to raise livestock, causing them to lose fat and into malnutrition or even die due to insufficient feed, which brought huge loss to herders' production. Under the promotion of the grassland ecological compensation policy, the herders changed the "year-round grazing" to "seasonal rotation grazing" or "regional rotation grazing", and implemented rest grazing and divisional rotation grazing in the grass-livestock balance area. The breeding method has gradually become an inevitable choice for herders’ livestock breeding. Herders raise livestock in sheds, so that herders can avoid weight loss and death caused by lack of pasture in winter and spring and cold weather. The herders will correspondingly improve the technical level of shed feeding and pay more attention to feeding in the breeding process. Feeding technology, disease control and livestock breeding systems have increased the rate of livestock slaughter and speeded up the turnover of the herd. But from another perspective, as the price of forage has been increasing year by year, the increase in the proportion of house-feeding will directly lead to an increase in the cost of breeding. At the same time, the Herders have a lower level of education, older age and poor ability to acquire new technology. The technical level of captive breeding has not been improved, so the direct impact of the grassland compensation policy on the breeding efficiency of herders is not clear.
- Path 2: GECP affects the efficiency of animal husbandry by changing the traditional breeding structure of Herders. Due to the reduction in the area of pasture available for grazing and the shortening of grazing time, the pastoral structure of Herders has also been changed, and the proportion of livestock that are more suitable for house feeding has expanded. At the same time, in order to reduce the cost of feeding and management, herders gradually choose single animal breeding. From this level, it also contributes to the improvement of herders’ breeding efficiency.
- Path 3: GECP affects breeding efficiency by promoting grassland circulation. Some herders will quit animal husbandry production and engage in other industries due to policy impacts[23]. At the same time, the circulation of grassland further affects the breeding efficiency of herders: the circulation of grassland makes the grassland resources flow from the less efficient livestock breeding, where herders want to withdraw from the livestock breeding activities, to the more efficient livestock breeding, where herders wish to obtain the rent from the pastoral circulation.The efficiency of grassland resource allocation can be optimized.
- Path 4: Grassland Ecological Compensation Funds affect the efficiency of breeding by changing the farming scale of herders. What cannot be ignored is that while the herders receive policy funds, it is possible to expand the scale of breeding. The development expands the boundaries of production possibilities for herders, and herders will choose to increase livestock breeding, which creates a scale effect. At the same time, herders also pay more attention to the use of new technologies and methods, and improve breeding technology by introducing more advanced livestock machinery to promote the improvement of breeding efficiency.

To sum up, this study will analyze the impact of GECP on herders’ breeding efficiency from the above four paths, that is, the direct effect of GECP on herders’ breeding technical efficiency; mediation effect 1-circulation effect; mediation effect 2-scale effect; mediation effect 3-structure effect.

According to the analysis in the theoretical framework, GECP will affect the re-allocation of input resources of herders from multiple paths of farming methods (direct effects), farming structure, farming scale, and grassland circulation (intermediary effects), thereby changing the overall farming efficiency of herders. Based on this, this study constructs the following parallel mediating effect model for analysis:

$Larea=a*policy+\sum\beta_{1i}Z_{i}+\mathbf{\varepsilon}_{1}$ （2）

$Scale=b*policy+\sum\beta_{2i}Z_{i}+\mathbf{\varepsilon}_{2}$ （3）

$Farmstr=c*policy+\sum\beta_{3i}Z_{i}+\mathbf{\varepsilon}_{3}$ （4）

$NEFF=d*policy+e*Larea+f*Scale+g*Farmstr+\sum\beta_{4i}Z_{i}+\mathbf{\varepsilon}_{4}$ （5）

Formula (2) analyzes the impact of the ecological compensation policy on the intermediary variable 1-herders’ grassland circulation, and the coefficient $a$ is the impact of the implementation of GECP on whether the herders carry out the grassland circulation; Formula (3) analyzes the intermediary variables of GECP 2——the impact of the scale of breeding. Among them, the coefficients $b$are the influence of GECP on the changes of herders’ breeding scale; Formula (4) is the impact of the ecological compensation policy on the intermediate variable 3-the herd farming structure, and the coefficient $c$is the impact of the implementation of GECP on the change of herd farming structure; Formula (5) simultaneously analyzes the influence of grassland circulation, farming scale, farming structure and GECP on the technical efficiency of herders’ farming. The coefficient $d$is the direct effect of GECP on the technical efficiency of farming after controlling the influence of the intermediary variables. $Z_{i}$is a series of control variables such as Education level of the head of the household, age of the head of the household, and etc. $\mathbf{\varepsilon}_{1}\text{、}\mathbf{\varepsilon}_{2}\text{、}\mathbf{\varepsilon}_{3}\text{、}\mathbf{\varepsilon}_{4}$ are model regression residuals. In the model, $a*e$、$b*f$、$c*g$represent the intermediary effect of grassland circulation, the intermediary effect of breeding scale and the intermediary effect of breeding structure, respectively. $d$ represents a direct effect. The steps of the test are as follows: first test the significance of the coefficient of the mediator regression variable; The second method is Sobel (1982) to test whether $a*e$、$b*f$、$c*g$are significant[24], and the third is to test whether the overall effect is significant, and the test method is to use the formula of Clogg et al. (1992) to test[25]. In the specific calculation process, we used the lavan package and mediation package in R[26, 27].

**4. Data and variables**

**4.1.** **Description of the study area**

**Figure 2 Locations of the study area**

The data used in this study comes from a field questionnaire interview on herders in the pastoral area of Inner Mongolia conducted by the research team. from June to November 2018. This herders’ survey was approved and carried out by the School of Economics and Management of Beijing Forestry University and the School of Finance and Economics of Jiangsu University, and was assisted by the local government. The main source of funding was the National Natural Science Foundation of China (No. 71704067). I would like to declare on behalf of my co-authors that the work described was original research that has not been published previously, and not under consideration for publication elsewhere, in whole or in part. All authors listed are aware of the attached manuscript and agree to submit it. We obtained the information of the participants’ informed consent and obtained written proof of their signed consent. And they are aware of the purpose of the survey data. In addition, our investigation does not involve minors.The Ethics Committee gave up the approval on the study, so we cannot provide an approval letter from the Ethics Committee. The reason is that the study is an evaluation study of the effects of GECP policy which did not involve any research on interviewee privacy issues, our survey content has been shown in the questionnaires, and the data we used have deleted the respondents' identifying information.

First, based on the regional characteristics of Inner Mongolia’s grasslands and pastoral areas, the Xinbarag Right(Hulunbeier City), Chenbarag Qi (Hulunbeier City), West Ujimqin (Xilinguole League) and Siziwang (Ulanchabu) were selected as the geographical distribution of the 4 sample counties is shown in the figure2. Then, taking into account the differences in population and grassland area, 4 to 8 sample towns (Sumu) were selected in each sample county, and a questionnaire survey was conducted with herders in the form of "one-to-one" interviews. The questionnaire survey includes the following aspects: basic characteristics of herders households (sex, age, education level, grassland area, etc.); basic household conditions (total population, household income level and structure, etc.); livestock production investment, Livestock breeding expenses, livestock income, living expenses, grassland compensation policy and other subsidies. A total of 449 valid questionnaires were collected in this survey.

**4.2. Variable selection and descriptive statistical analysis**

There are two aspects in the selection of herders' livestock breeding efficiency indicators: input variables and output variables. With reference to previous research results (Sun Zhilu et al., 2014; Wang Xuejiao, 2018)[28, 29], In this study, the amount of livestock produced in the current year (standard sheep unit) was selected as the output variable, and selected the feed cost (total purchased feed + total purchased forage + Total straw purchased + total feed salt), actual input area of grassland (own pasture area-grazing prohibited area + rented area-rented area), labor input (family labor input + annual number of employees) and other expenses (epidemic prevention expenses) +Medical Expenses + Repair Expenses + Fuel Expenses + Financial Expenses + Grassland Construction Expenses) as input variables (see Table 1). The conversion of the standard adult sheep unit is: 1 lamb is converted into 0.4 standard sheep, 1 cattle is converted into 6 standard sheep, 1 horse is converted into 6 standard sheep, and 1 camel is converted into 7 standard sheep.

In terms of factors affecting the efficiency of herders’ breeding technology, this study mainly explores the impact of GECP on the efficiency of herders’ breeding technology after the implementation of GECP. According to the previous theoretical analysis, as far as animal husbandry is concerned, the implementation of the grassland ecological compensation policy, whether it is grazing prohibition or the balance of forage and animal husbandry, has the most direct impact on herders due to the restriction of herding behavior, thus prompting herders to change the scale of breeding, breeding structure and breeding methods. However, grazing prohibition and forage-livestock balance have different effects on the utilization of pastures of herders. Therefore, this study selects the value of compensation funds for pasture-livestock balance to represent the impact of the policy on the utilization of pastures for herders.

**Table 1. Statistical description of input and output variables of herders' livestock breeding**

| **variable** | **Variable description** | **mean** | **sd** |
| --- | --- | --- | --- |
| Y | Total livestock production in the year (standard sheep) | 375.93 | 384.10 |
| area | Self-owned pasture area-grazing prohibited area+  leased in area - rented out area（ha） | 35.25 | 45.39 |
| lab | Family labor input + annual number of employees | 4.08 | 1.68 |
| feed | Total amount of feed purchased + total amount of purchased pasture +  total purchased straw + total feed salt($) | 222.23 | 288.20 |
| other | Epidemic prevention cost + medical cost + repair cost +  fuel cost +financial cost + grassland construction cost（$） | 840.21 | 774.52 |

At the same time, after the implementation of GECP, some herders will participate more in non-grass industry employment and transfer the grassland out due to the changes in traditional breeding methods. Some herders may enter the grassland to maintain or expand their original scale of farming and improve their farming methods due to the limited use of grassland and the lack of conditions to engage in non- animal husbandry. On the one hand, the transfer circulation has changed the scale of the inflow side and reduced the breeding cost; On the other hand, the circulation often promotes the transfer of pastures from pastures with low breeding efficiency to herders with high breeding efficiency. Therefore, this section will take the grassland circulation as an intermediary variable to investigate whether GECP indirectly affects the technical efficiency of herders through the circulation mode, and whether the herders are net transferred into the grassland (leased in area - leased out area > 0) represents the grassland circulation behavior of herders.

In addition, the existing literature found that the characteristics of herders and household resource endowment, location endowment, market price and grassland rainfall and other factors will have an impact on the efficiency of livestock farming technology. Therefore, the age of the head of the herders, the education level of the head of the household, the number of family population, the proportion of non pastoral income, the geographical distance from the county (banner) government, the geographical distance from the nearest livestock trading market, and regional variables are selected as the control variables of this study[15-18]. And the descriptive statistics of the corresponding variables are as follows(Table 2):

**Table 2. Descriptive statistics of variables affecting the efficiency of breeding technology**

| **variable** | **Variable description** | **mean** | **sd** |
| --- | --- | --- | --- |
| larea | Area of leased pasture-area of leased pasture> 0 to take 1, otherwise 0 | 0.27 | 0.45 |
| farnstr | Number of the animal which is the most feeded / total animals feeded | 0.69 | 0.18 |
| scale | Breeding scale(100 standard sheep) | 0.88 | 1.00 |
| age | Age of head of household | 45.16 | 11.40 |
| edu | Education level of the head of the household | 3.15 | 0.96 |
| policy | Average amount of compensation for herders per  mu of grassland in that year (100$/ha) | 0.09 | 0.09 |
| inc | Household income of herders in the year (1000$) | 12.66 | 12.22 |
| incstr | Non-animal husbandry income accounted for the  total household income in the year(%) | 0.04 | 0.09 |
| lab | Number of labor force of herders’ household in that year | 3.20 | 1.30 |
| dis1 | Distance to the banner (county) government(100km) | 0.70 | 0.32 |
| dis2 | Distance to the nearest livestock market(100km) | 0.50 | 0.24 |
| price | Average selling price of herders in the year ($/standard sheep) | 112.73 | 3.71 |

# 5. Model Results

## 5.1. Efficiency Evaluation of Breeding Technology

According to DEA calculations, the technical efficiency of herders' livestock breeding is obtained, as shown in Table3. It can be seen that the average technical efficiency of herders breeding in the surveyed area in 2010 was 0.5916, while that in 2017 rised to 0.6731, with an overall increase of 0.081. Therefore, under the background of the implementation of grassland ecological supplement award, the efficiency of herders’ breeding has been improved in this period.From the perspective of technical efficiency grouping, compared with 2010, the number of herders with efficiency lower than 0.3 in 2017 decreased significantly, from 3.34% of the original sample to 2.00%. At the same time, in the efficiency groups of 0.3-0.5, 0.5-0.7 , the proportion of herders decreased to varying degrees, with the decrease of 0.5-0.7 being the most obvious, while the number of herders in the high efficiency group of 0.9-1 increased significantly, from 3.79% of the original sample to 16.26%. On the whole, the breeding efficiency of herders in different efficiency levels has been improved to some extent.

**Table 3. Frequency distribution of breeding efficiency of sample herders**

| **Breeding**  **efficiency** | **2010** | | | **2017** | | |
| --- | --- | --- | --- | --- | --- | --- |
|  | **Number of**  **samples** | **Ratio** | **Cumulative**  **ratio** | **Number of**  **samples** | **Ratio** | **Cumulative**  **ratio** |
| 0~0.3 | 15 | 3.34% | 3.34% | 9 | 2.00% | 2.00% |
| 0.3~0.5 | 126 | 28.06% | 31.40% | 86 | 19.15% | 21.15% |
| 0.5~0.7 | 200 | 44.54% | 75.94% | 153 | 34.08% | 55.23% |
| 0.7~0.9 | 91 | 20.27% | 96.21% | 128 | 28.51% | 83.74% |
| 0.9~1 | 17 | 3.79% | 100.00% | 73 | 16.26% | 100.00% |
| mean | 0.5916 | | | 0.6731 | | |

Then, t-test was conducted on the average efficiency of herders’ breeding in 2010 and 2017, and the results are shown in Table 4. According to the test results, the efficiency of herders in Hulunbuir grassland in 2017 was significantly higher than that in 2010, which shows that the efficiency of herders breeding has improved under the implementation of grassland ecological compensation policy.

**Table 4. t-test results on the average efficiency of herders’ breeding in 2010 and 2017**

| **Original hypothesis** | **P-value** | **Result** | **Conclusion** |
| --- | --- | --- | --- |
| Eff2010=Eff2017 | 0.000 | Reject | Eff2010≠Eff2017 |
| Eff2010>Eff2017 | 0.000 | Reject | Eff2010<Eff2017 |

## 5.2. The influence path of GECP on the efficiency of herders’ farming technology

In order to verify the influence mechanism of GECP on the efficiency of herders’ breeding technology, the variables involved in the model were sorted out, and the estimation steps of the mediating effect model were used to establish the model, Table 5 shows the estimation results:

First, the results of Model 1 show that GECP effectively promote the grassland circulation among herders at P<0.01(1%). After the implementation of GECP, the available grassland area of herders will be affected, and some herders will withdraw from animal husbandry to engage in other industries, and some herders will make use of grassland ecological compensation funds to further expand the scale of animal husbandry[30, 31]. At the same time, observing the model results of Model 2, we can find that herders’ grassland circulation promotes the increase of herders’ breeding scale. An intuitive explanation is that herders get more grassland through the circulation, and their production possibility boundary moves out. At the same time, the grassland transfer in Model 4 improves the efficiency of herders. Grassland circulation will form an efficiency leveling effect. Herders with low breeding efficiency will gradually withdraw from pastoral production and transfer the pasture to herders with high breeding technology efficiency, forming a Pareto improvement in the allocation of grassland resources. Therefore, combined with the promotion of grassland circulation by GECP - grassland circulation significantly improves the technical efficiency of herders’ breeding technology, and it can be concluded that the intermediary effect of grassland circulation is significant at P<0.01(1%).

**Table 5. Estimation results of parallel mediating effects model of the effect of GECP on the efficiency of breeding technology**

|  | Dependent variable: | | | |
| --- | --- | --- | --- | --- |
|  | larea | scale | farmstr | eff |
| scale |  |  |  | 0.026*** |
|  |  |  |  | (-0.008) |
| farmstr |  |  |  | 0.013 |
|  |  |  |  | (-0.029) |
| larea |  |  |  | 0.088*** |
|  |  |  |  | (-0.012) |
| policy | 6.584*** | 8.893*** | 0.512 | 3.750*** |
|  | (-0.825) | (-1.211) | (-0.34) | (-0.316) |
| inc | 0.001 | -0.001 | 0.002*** | -0.002*** |
|  | (-0.001) | (-0.002) | (-0.001) | (-0.0004) |
| incstr | 0.161 | 0.29 | -0.011 | 0.043 |
|  | (-0.172) | (-0.252) | (-0.071) | (-0.061) |
| price | -0.001 | 0.002 | 0.001 | -0.003** |
|  | (-0.004) | (-0.006) | (-0.002) | (-0.001) |
| age | 0.004*** | -0.001 | 0.0002 | 0.002*** |
|  | (-0.001) | (-0.002) | (-0.001) | (-0.0005) |
| edu | -0.034** | -0.008 | -0.003 | 0.008 |
|  | (-0.015) | (-0.022) | (-0.006) | (-0.005) |
| lab | 0.022* | -0.005 | -0.012*** | 0.003 |
|  | (-0.011) | (-0.017) | (-0.005) | (-0.004) |
| dis1 | 0.125** | -0.046 | -0.035* | -0.025 |
|  | (-0.05) | (-0.073) | (-0.02) | (-0.018) |
| dis2 | -0.065 | -0.031 | -0.139*** | 0.037 |
|  | (-0.066) | (-0.097) | (-0.027) | (-0.024) |
| Constant | 361.463*** | 518.410*** | 27.871 | 213.133*** |
|  | (-52.477) | (-76.968) | (-21.597) | (-19.821) |
| Year | Yes | yes | yes | yes |
| Firm | Yes | yes | yes | yes |
| R2 | 0.141 | 0.634 | 0.1 | 0.379 |
| F Statistic | 13.170*** | 139.598*** | 8.989*** | 38.530*** |

*** p<0.01, ** p<0.05, * p<0.1

Second, when examining whether there is a mediating effect when breeding scale is used as an intermediary variable, the results of Model 2 show that although GECP stipulates the livestock carrying capacity per unit grassland, the livestock breeding scale of herders does not decline at P<0.01(1%). On the contrary, the higher the amount of subsidy, the more likely the herders will not reduce the number of livestock breeding. Herders use the subsidy funds to build captive breeding facilities to make up for grazing prohibition and the loss caused by the balance between grass and livestock. This shows that the current GECP makes herders increase the number of livestock breeding. Existing research explained that the grassland subsidy policy is not conducive to livestock reduction as follows: at present, GECP has the problem of mismatching the amount of subsidy and the livestock reduction behavior of farmers and herders, and the actual livestock reduction behavior occurs, and the farmers and herders with higher livestock reduction rate do not get more subsidy funds, resulting in the policy unable to mobilize the enthusiasm of farmers and herders to reduce livestock [40-42]. At the same time, the scale of herders in Model 4 has a positive impact on the breeding efficiency, and the reason is that the breeding cost of herders decreases with the increase of scale and the efficiency of breeding is improved.

Thirdly, when examining whether there is a mediating effect when Feeding structure is used as an intermediary variable, the results of Model 3 show that the effect of GECP on Feeding structure has not passed the significance test, so the mediating effect of breeding structure is not tenable at P>0.1 (10%).

Fourth, from the results of Model 4, we can find that the direct effect of GECP on the efficiency of herders' breeding is significantly positive at P<0.01(1%).The implementation of GECP has changed the traditional breeding mode to semi stocking + captive farming mode, which has changed the traditional extensive animal husbandry management mode. Although in the short term, it will have an impact on the traditional grazing breeding mode of herders, reduce the grazing time of herders, reduce the stocking rate of grassland, and increase the cost investment of forage and enclosure construction in the process of breeding, but the reward fund is added and it can also alleviate the economic pressure of the transformation of livestock farming mode to a certain extent, Moreover, as a long-term institutional arrangement, GECP is bound to force farmers and herders to change their development mode, improve their breeding management ability and breeding technology level, so as to speed up the breeding turnover and increase the live weight of breeding, and eventually lead to the improvement of livestock breeding technical efficiency.

Fifth, the direct effect of GECP on the technical efficiency of herders and whether the mediating effect is partial mediating effect or masking effect was investigated.The results of Model 4 showed that the direct effect of GECP on livestock breeding technical efficiency was significantly positive at P<0.01(1%), and the scale effect and grassland circulation effect were both positive and the symbol was the same as the direct effect (3.750, P<0.01), indicating that the grassland circulation and scale of cultivation were partial mediating effects in the impact of GECP on the technical efficiency of livestock breeding, that is, GECP was directly positive by changing the breeding mode of herders to promote the efficiency of livestock breeding technology. The direct effect accounts for 82.22% of the total effect, and the indirect effect accounts for 17.78% of the total effect(Table,6).The total effect of GECP on the technical efficiency of livestock breeding is positive under the two action paths, which is consistent with the research conclusions of Liu et al. (2019) and Han et al. (2019)[14, 32].

**Table 6. Grassland Ecological Protection Subsidy Policy affects the results of path analysis of herder breeding technology efficiency**

|  | Theoretical path | coefficients | Path coefficients | Total path coefficient  (effect ratio) |
| --- | --- | --- | --- | --- |
| ADE  （Direct effect） |  | $d$ | 3.750***  (0.312) | 3.750***  （82.22%） |
| ACME  (Indirect effect) | Policy→Structure→EFF | $d*n$ | 0.007  (0.015) | 0.811***  （17.78%） |
|  |  |  |  |  |
|  | Policy→Scale→EFF | $b*f$ | 0.227***  (0.078) |  |
|  |  |  |  |  |
|  | Policy→Circulation→  （ADE）/Scale(ACME)→EFF | $c*g$ | 0.577***  (0.106) |  |
|  |  |  |  |  |
| ATE  （Total effect） |  |  | 4.561***  (0.303) | 4.561***  （100%） |

*** p<0.01, ** p<0.05, * p<0.1

# 6.Discussion:GECP's Heterogeneous Impact between different herders

In order to further analyze the heterogeneous impact of GECP on the breeding efficiency of herders, we divided the samples into small-scale samples (the breeding scale is less than the average) and large-scale samples (the breeding scale is greater than the average) according to the average breeding scale of the herders in 2010. The two samples established a parallel mediation effect model for analysis, and the results obtained are shown in Table 7 as follows:

First, Whether in a sample of small-scale herders or a sample of large-scale herders, it can be found that the impact of GECP on herders’ feeding efficiency is still dominated by direct effects (80.27%)，The mediation effect (19.73%) is in the same direction as the direct effect, so it is part of the mediation effect. This result is generally consistent with the result of the overall sample.

**Table 7. Grassland Ecological Protection Subsidy Policy affects the results of path analysis of herder breeding technology efficiency: Based on different feeding scales**

| Scale | Effect | Theoretical path | Path coefficients | Total path coefficient  (effect ratio) |
| --- | --- | --- | --- | --- |
| Small  Scale | ADE  （Direct effect） |  | 3.712***  (0.427) | 3.712***  (0.427)  （80.27%） |
|  | ACME  (Indirect effect) | Policy→Structure→EFF | 0.003  (0.012) | 0.912***  （0.190）  （19.73%） |
|  |  | Policy→Scale→EFF | -0.010  (0.032) |  |
|  |  | Policy→Circulation  →EFF | 0.919***  (0.427) |  |
|  | ATE  （Total effect） |  | 4.624***  (0.431) | 4.624***  (0.431)  （100%） |
| Large  Scale | ADE  （Direct effect） |  | 3.608***  (0.438) | 3.608***  (0.438)  （79.62%） |
|  | ACME  (Indirect effect) | Policy→Structure→EFF | 0.036  (0.438) | 0.924***  （0.196）  （20.38%） |
|  |  |  |  |  |
|  |  | Policy→Scale→EFF | 0.551**  (0.149) |  |
|  |  |  |  |  |
|  |  | Policy→Circulation  →EFF | 0.336***  (0.116) |  |
|  | ATE  （Total effect） |  |  |  |
|  |  |  | 4.531***  (0.423) | 4.531***  (0.423)  （100%） |

*** p<0.01, ** p<0.05, * p<0.1

Second, by comparing the results of a sample of small-scale herders and a sample of large-scale herders, some differences can be found：First of all, the direct effect of GECP on small-scale herders is greater than the impact on large-scale herders (3.712>3.608), indicating that the implementation of GECP has a greater impact on the traditional and extensive breeding methods of small-scale herders than on large-scale herders, Small-scale herders use GECP funds to turn to captive breeding, and relatively speaking, large-scale herders’ facilities and housing conditions are due to small-scale herders, Therefore, after the implementation of GECP, the effect of improving the raising efficiency of herders by changing the feeding method is weaker than that of small-scale herders. Secondly, the mediation effect part: the total mediation effect of the two samples is very close (0.912 and 0.924), but the effects of each mediation part are different, The size-mediating effect of small-scale herders did not pass the significance test, while the size-mediating effect of large-scale herders was significantly positive (0.551)，It shows that the original large-scale herders are easier to adjust their own breeding scale to reach the optimal scale after the implementation of the GECP policy, while the small-scale herders did not form a scale effect due to the small basic breeding scale and shortage of funds，As shown in Table 8, the impact of GECP on the breeding scale of small-scale herders is not significant. In addition, the grassland circulation effect of large-scale herders (0.336) is smaller than that of small-scale herders (0.919)，There are two main reasons: On the one hand, the GECP policy makes small-scale herders more enthusiastic about flowing into the pasture, and small-scale herders tend to have smaller pastures, Generally, there is no basic pasture for maintaining livestock production after the implementation of GECP, so it is more inclined to flow into pastures than large-scale herders; on the other hand, renting pastures improves the feeding efficiency of small-scale herders more (Table 8).

**Table 8. Estimation results of parallel mediating effects model of the effect of GECP on the efficiency of breeding technology: Based on different feeding scales**

|  | | Dependent variable: | | | |
| --- | --- | --- | --- | --- | --- |
|  |  | Larea | scale | farmstr | eff |
| Small  Scale | scale |  |  |  | -0.044 |
|  |  |  |  |  | (-0.028) |
|  | farmstr |  |  |  | -0.010 |
|  |  |  |  |  | (-0.039) |
|  | Larea |  |  |  | 0.113*** |
|  |  |  |  |  | (-0.016) |
|  | policy | 8.340*** | 0.221 | -0.282 | 3.712*** |
|  |  | (-1.245) | (-0.72) | (-0.512) | (-0.436) |
| Large  Scale | scale |  |  |  | 0.046*** |
|  |  |  |  |  | (-0.01) |
|  | farmstr |  |  |  | 0.03 |
|  |  |  |  |  | (-0.042) |
|  | Larea |  |  |  | 0.060*** |
|  |  |  |  |  | （-0.017） |
|  | policy | 5.607*** | 12.056*** | 1.204** | 3.608*** |
|  |  | （-1.145） | （-1.954） | （-0.469） | （-0.448） |

*** p<0.01, ** p<0.05, * p<0.1

# 7. Conclusions

Based on 449 herders’ micro data obtained from field survey, this study empirically tested the impact of grassland ecological compensation policy on herders’ breeding efficiency and the mediating effects of grassland circulation, breeding scale and breeding structure in the above effects. The conclusions are as follows: (1) Since the implementation of GECP, the breeding scale of sample herders has not decreased but increased. Although the livestock carrying capacity of herders per unit grassland is required by grazing prohibition + livestock balance, in fact, the number of breeding households is increasing; (2) GECP has a positive impact on the overall technical efficiency of the herders. The supplementary fund relieves the economic pressure of the transformation of the breeding mode of the herders. As a long-term institutional arrangement to ensure the sustainable development of grassland animal husbandry, GECP forces the herders to change the development mode and improve the livestock breeding efficiency; (3) The mediating effect of grassland circulation and breeding scale was established, and the mediating effect accounted for 19.73% respectively. That is to say, GECP mainly depends on changing the breeding mode of herders to improve the technical efficiency of herders. At the same time, it indirectly improves the breeding efficiency of herders by increasing the breeding scale of herders and promoting the grassland circulation of herders. (4) Although the effect of GECP on the feeding efficiency of herders of different sizes is basically the same, there are still some differences. Generally speaking, the effect of GECP on the raising efficiency of small-scale herders is greater than that of large-scale herders. In terms of the direct effect of changing the feeding method, the small-scale herders are greater than the large-scale herders. In terms of the indirect utility of the promotion of grassland circulation and expansion scale, GECP can improve the feeding efficiency of large-scale herders more than small-scale herders.

# 8. Recommendations

Based on the above research conclusions, this study puts forward the following policy implications: (1) While continuing to implement the current grassland ecological protection compensation policy, we should formulate corresponding supporting policies to support the transformation and development of animal husbandry in pastoral areas through technology and funds, such as the policy support for the construction of breeding land and for beef cattle grazing to house feeding, forming a joint force with the ecological compensation policy to promote the transformation, upgrade the development of animal husbandry, and finally improve the breeding efficiency; (2) The implementation of GECP makes some herders gradually give up animal husbandry production and turn to other industries. Therefore, the local government should actively establish corresponding grassland circulation platform to avoid grassland waste and improve resource utilization efficiency; (3) The local government should strengthen the training of herders' grazing and breeding skills. With the implementation of GECP, house feeding and semi house feeding have gradually become the main mode of animal husbandry production of herders. Improving the feeding and management mode of house feeding and semi house feeding has become an important link for raising efficiency of herders.

# 9. Limitations and future work

This study not only verified the positive impact of grassland ecological compensation policy on the technical efficiency of herders’ breeding, but also used the intermediary effect model to analyze the path of action, which made up for the lack of relevant research. However, the data in this study are field survey data, which makes it difficult to investigate herders. And the sample coverage is only limited to Hulunbuir Grassland Area, not including all policy implementation areas. Therefore, this is one of the shortcomings of this study. In the future, we will continue to expand the research area for further research.

**Acknowledgments:** We wish to thank PhD Jin Yi from Leiden University for providing valuable suggestions that improved final versions of this manuscript.

**Author Contributions:**

Conceptualization, Y.W.; data curation,Y.Q.H.; formal analysis,Y.W.; methodology, Y.W. and W.C.L.; supervision, Y.J.H.; writing—original draft, Y.W. and W.C.L.; writing—review and editing, Y.W. and W.C.L.

All authors have read and agreed to the published version of the manuscript.

**Conflicts of Interest:** The authors declare no conflict of interest.

References

1.	Hua L, Squires VR. Managing China's pastoral lands: Current problems and future prospects. Land Use Policy. 2015;43:129-37. doi: 10.1016/j.landusepol.2014.11.004.

2.	Wang H, Zhou X, Wan C, Fu H, Zhang F, Ren J. Eco-environmental degradation in the northeastern margin of the Qinghai Tibetan Plateau and comprehensive ecological protection planning. Environmental Geology. 2008;55:1135. doi: 10.1007/s00254-007-1061-7.

3.	Squires D. Biodiversity Conservation in Asia. Asia & the Pacific Policy Studies. 2014;1(1):144-59. doi: 10.1002/app5.13.

4.	Li S, Verburg PH, Lv S, Wu J, Li X. Spatial analysis of the driving factors of grassland degradation under conditions of climate change and intensive use in Inner Mongolia, China. Regional Environmental Change. 2011;12(3):461-74. doi: 10.1007/s10113-011-0264-3.

5.	Kemp D, Brown C, Han G, Michalk D, Nan Z, Wu J, et al. Chinese grasslands: Problems, dilemmas, and finding solutions. In: Kemp DR, Michalk DL, editors. XXI International Grassland Congress and VIII International Rangeland Congress. Canberra, ACT: Australian Centre for International Agricultural Research (ACIAR); 2011. p. 12-25.

6.	Brown K. Book Reviews : David SNEATH, Changing Inner Mongolia: Pastoral Mongolian Society and the Chinese State. Oxford: Oxford University Press, 2000. Oxford Studies in Social and Cultural Anthropology. 320 pp., with maps and index. ISBN: 0-19-823413-9. Price: £45.00. China Information. 2001;15(1):326-9. doi: 10.1177/0920203x0101500153.

7.	(MOA) MoAotPsRoC. Guidelines for the Implementation of the Grassland Ecological Conservation Subsidy and Incentive Policy in 2011. 2011:1-11.

8.	Xudong Y, Zhixing M, Chun Y. Effects of Grassland Eco-Protection Compensation and Reward System.. 2016;17(6):1506-9. PubMed PMID: 117209330.

9.	Aijun L. Analysis of the effects of subsidies and incentives for grassland ecological protection in Inner Mongolia and its problems. Grassland and Prataculture. 2014;26(02):4-8.

10.	Liu M, Dries L, Heijman W, Huang J, Zhu X, Hu Y, et al. The Impact of Ecological Construction Programs on Grassland Conservation in Inner Mongolia, China. Land Degradation & Development. 2018;29(2):326-36. doi: 10.1002/ldr.2692.

11.	Haichun W, bo G. An Empirical Analysis of the Impact of the Grassland Ecological Protection Subsidy and Reward Mechanism on Herdsmen's Livestock Reduction Behavior——Based on a Survey of 260 Herdsmen in Inner Mongolia. Issues in Agricultural Economy. 2017;38(12):73-80+112.

12.	Hu Y, Huang J, Hou L. Impacts of the Grassland Ecological Compensation Policy on Household Livestock Production in China: An Empirical Study in Inner Mongolia. Ecological Economics. 2019;161:248-56. doi: <https://doi.org/10.1016/j.ecolecon.2019.03.014>.

13.	Jin-ya L, Jian-liang X, Xu-dong S, Bing-long L. Otherness Analysis on the Subjects of Indemnification about “Balancing Grass and Livestock” Compensation Policy:Variation Analysis of Herdsmen with Different Scales of Pastureland on “Balance of Forage and Livestock”. China Population,Resources and Environment. 2014;24(11):89-95.

14.	Chun-peng L, Hai-feng X. Grazing ban policy, breeding scale and technical efficiency:A case study of sheep farmers from five provinces. Research of Agricultural Modernization. 2019;40(01):138-44.

15.	Ning Z, fu Q. Analysis of the effect of large-scale livestock and poultry farming under environmental endogenous conditions: taking laying hens as an example. Rural Economy. 2016;(01):50-6.

16.	Xudong Y, Chun Y, Zhixing M. Analysis of the effect of grassland ecological protection compensation policy on animal husbandry production——Based on the investigation of Youyu in Shanxi. Agricultural Economy. 2018;(10):51-3.

17.	Yanjiao W, Jing Z. Effects of dairy imports on the efficiency of cow farming operations of different scales in China. Resources Science. 2019;41(08):1475-87.

18.	Jie L, Xiangdong H, Yubin W. Analysis of Pig Breeding Efficiency and its Influence Factors——A Survey of 277 Farmers in Four Provinces in China. Journal of Agrotechnical Economics. 2019;(08):29-39.

19.	Chun Y, Mingli W. Research on Beef Cattle Total Factor Productivity Growth and Convergence in the Context of Grassland Ecological Protection Subsidy Policy. Journal of Agrotechnical Economics. 2019;(03):96-105.

20.	Xin S, Haolong L. The Effects of Farmers' Specialized Cooperatives on Agricultural Production Efficiency within the Context of Rural Labor Migration. Chinese Rural Economy. 2017;(05):58-72.

21.	Lansink AO, Reinhard S. Investigating technical efficiency and potential technological change in Dutch pig farming. Agricultural Systems. 2004;79(3):353-67. doi: <https://doi.org/10.1016/S0308-521X(03)00091-X>.

22.	Lizhong Z, Jianwei P, Jiancheng C. Measurement of Moderate Management Scale of Animal Husbandry in Different Grassland Type Areas. Issues in Agricultural Economy. 2012;33(04):90-7.

23.	Huihui L. Research on Grassland Circulation Behavior of Herdsmen in Inner Mongolia Pastoral Area and Its Impact on Grassland Ecology [硕士]: China Lanzhou University; 2020.

24.	Sobel ME. Asymptotic Confidence Intervals for Indirect Effects in Structural Equation Models. Sociological Methodology. 1982;13:290-312. doi: 10.2307/270723.

25.	Clogg CC, Petkova E, Shihadeh ES. Statistical methods for analyzing collapsibility in regression models. Journal of Educational Statistics. 1992;17(1):51-74.

26.	Tingley D, Yamamoto T, Hirose K, Keele L, Imai K. Package ‘mediation’. 2019.

27.	Rosseel Y. lavaan: An R Package for Structural Equation Modeling. 2012. 2012;48(2):36. Epub 2012-05-24. doi: 10.18637/jss.v048.i02.

28.	Xuejiao W, Haifeng X. Allocation efficiency of farmers' meat sheep production in China and its influencing factors. Journal of Arid Land Resources and Environment. 2018;32(03):88-93.

29.	Zhi-lu S, Xian-de L. Influence of Agricultural FDI on China's Total Factor Productivity: An Estimation Based on Stochastic Frontier Function Model for Panel Data. International Business. 2014;(03):54-62.

30.	Shengqiang Z, Kai Z. How Does the Grassland Ecological Compensation Policy Affect Farmers and Herdsmen's Behavior of Reducing Livestock: an Analysis Based on the Regulatory Effect of Non-agricultural and Pastoral Employment. Issues in Agricultural Economy. 2019;(11):108-21.

31.	Panxian W, Xiaohui Q, Guanghuai Q. Research on the Incentive Effect of Grassland Ecological Protection Compensation Policy on Herdsmen's Livestock Reduction Behavior. Heilongjiang Animal Science and Veterinary Medicine. 2020;(06):20-6.

32.	Zhen H, Chun Y, Xinxin Z. Analysis of Total Factor Productivity of Mutton Sheep Breeding in Pastoral Areas under Ecological Compensation and Reward Mechanism. Journal of Agrotechnical Economics. 2019;(11):116-26.
