# Supplementary material for: Does the grassland ecological compensation policy improve the herders’ breeding technical efficiency in China?—Based on the parallel mediation effect model
Source: PLoS One. 2021 Apr 29;16(4):e0249990. doi: 10.1371/journal.pone.0249990 (PMC8084240; doi:10.1371/journal.pone.0249990)
Supplement: Peer review history

# Peer review: Decision Letter 0

**DOI:** 10.1371/journal.pone.0249990.r001

**Authors:** Bing Xue

13 Nov 2020

PONE-D-20-31234

Does the Grassland Ecological Compensation Policy improve the herders' breeding technical efficiency in China?—Based on the parallel mediation effect model

PLOS ONE

Dear Dr. Li,

Thank you for submitting your manuscript to PLOS ONE. After careful consideration, we feel that it has merit but does not fully meet PLOS ONE’s publication criteria as it currently stands. Therefore, we invite you to submit a revised version of the manuscript that addresses the points raised during the review process.

Please submit your revised manuscript by Dec 28 2020 11:59PM. If you will need more time than this to complete your revisions, please reply to this message or contact the journal office at plosone@plos.org. When you're ready to submit your revision, log on to https://www.editorialmanager.com/pone/ and select the 'Submissions Needing Revision' folder to locate your manuscript file.

Please include the following items when submitting your revised manuscript:

- A rebuttal letter that responds to each point raised by the academic editor and reviewer(s). You should upload this letter as a separate file labeled 'Response to Reviewers'.
- A marked-up copy of your manuscript that highlights changes made to the original version. You should upload this as a separate file labeled 'Revised Manuscript with Track Changes'.
- An unmarked version of your revised paper without tracked changes. You should upload this as a separate file labeled 'Manuscript'.

If you would like to make changes to your financial disclosure, please include your updated statement in your cover letter. Guidelines for resubmitting your figure files are available below the reviewer comments at the end of this letter.

If applicable, we recommend that you deposit your laboratory protocols in protocols.io to enhance the reproducibility of your results. Protocols.io assigns your protocol its own identifier (DOI) so that it can be cited independently in the future. For instructions see: http://journals.plos.org/plosone/s/submission-guidelines#loc-laboratory-protocols

We look forward to receiving your revised manuscript.

Kind regards,

Bing Xue, Ph.D.

Academic Editor

PLOS ONE

Journal Requirements:

When submitting your revision, we need you to address these additional requirements.

1) Please ensure that your manuscript meets PLOS ONE's style requirements, including those for file naming. The PLOS ONE style templates can be found at

https://journals.plos.org/plosone/s/file?id=wjVg/PLOSOne_formatting_sample_main_body.pdf and

https://journals.plos.org/plosone/s/file?id=ba62/PLOSOne_formatting_sample_title_authors_affiliations.pdf

2) Please provide additional details regarding participant consent. In the ethics statement in the Methods and online submission information, please ensure that you have specified (1) whether consent was informed and (2) what type you obtained (for instance, written or verbal, and if verbal, how it was documented and witnessed). If your study included minors, state whether you obtained consent from parents or guardians. If the need for consent was waived by the ethics committee, please include this information.

3) Please include additional information regarding the survey or questionnaire used in the study and ensure that you have provided sufficient details that others could replicate the analyses. For instance, if you developed a questionnaire as part of this study and it is not under a copyright more restrictive than CC-BY, please include a copy, in both the original language and English, as Supporting Information.

4) Please remove your figures from within your manuscript file, leaving only the individual TIFF/EPS image files, uploaded separately.  These will be automatically included in the reviewers’ PDF.

5) Please include captions for your Supporting Information files at the end of your manuscript, and update any in-text citations to match accordingly. Please see our Supporting Information guidelines for more information: http://journals.plos.org/plosone/s/supporting-information.

[Note: HTML markup is below. Please do not edit.]

Reviewers' comments:

Reviewer's Responses to Questions

**Comments to the Author**

1. Is the manuscript technically sound, and do the data support the conclusions?

The manuscript must describe a technically sound piece of scientific research with data that supports the conclusions. Experiments must have been conducted rigorously, with appropriate controls, replication, and sample sizes. The conclusions must be drawn appropriately based on the data presented. 

Reviewer #1: Yes

Reviewer #2: No

Reviewer #3: Yes

Reviewer #4: Yes

Reviewer #5: Yes

Reviewer #6: Yes

**********

2. Has the statistical analysis been performed appropriately and rigorously? 

Reviewer #1: Yes

Reviewer #2: N/A

Reviewer #3: Yes

Reviewer #4: I Don't Know

Reviewer #5: Yes

Reviewer #6: Yes

**********

3. Have the authors made all data underlying the findings in their manuscript fully available?

The PLOS Data policy requires authors to make all data underlying the findings described in their manuscript fully available without restriction, with rare exception (please refer to the Data Availability Statement in the manuscript PDF file). The data should be provided as part of the manuscript or its supporting information, or deposited to a public repository. For example, in addition to summary statistics, the data points behind means, medians and variance measures should be available. If there are restrictions on publicly sharing data—e.g. participant privacy or use of data from a third party—those must be specified.

Reviewer #1: Yes

Reviewer #2: Yes

Reviewer #3: Yes

Reviewer #4: Yes

Reviewer #5: Yes

Reviewer #6: Yes

**********

4. Is the manuscript presented in an intelligible fashion and written in standard English?

PLOS ONE does not copyedit accepted manuscripts, so the language in submitted articles must be clear, correct, and unambiguous. Any typographical or grammatical errors should be corrected at revision, so please note any specific errors here.

Reviewer #1: Yes

Reviewer #2: No

Reviewer #3: Yes

Reviewer #4: Yes

Reviewer #5: Yes

Reviewer #6: No

**********

5. Review Comments to the Author

Please use the space provided to explain your answers to the questions above. You may also include additional comments for the author, including concerns about dual publication, research ethics, or publication ethics. (Please upload your review as an attachment if it exceeds 20,000 characters)

Reviewer #1: Generally,the manuscript is technically sound, and the data support the conclusions; the statistical analysis been performed appropriately and rigorously; and the manuscript presented in an a good fashion and written in standard English.

However,the author(s) should make corrections and revise the following parts;

1.General comments

-The font type should be checked,the font type of the title should be the same with other parts of the manuscript

-Set your computer to English language to avoid mixing up of Chinese and English font type. Example,the commas used in the lines 311,312,and 316, together with the character used in line 527 are in Chinese format,Please consider to change them to English format.

-The whole manuscript text should be double-spaced when submitting

-Indicate the number on headings and subheadings(example. 1.Introductions,2.Materials and Methods,2.1.Description of the study area etc.

-Indicate the continuous page numbers on the manuscript

-The language quality score is 48/60, which is good,but try to recheck for minor improvements.The minor issues to check are; punctuation,article usage,prepositions,singular-plural form,subject-verb agreement,verb form, and avoid unnecessary word capitalization

-Consider replacing the word "herdsmen" with "herders" if applicable in the given context in the whole manuscript(consider uniformity when using the words)

-Consider replacing the word "paper" with "study" if applicable in the given context, or use the preferred word whether the study,paper, or article on the whole manuscript(these words been used in the lines 45,178,218,226,,292,322,3345, and 353)

-Indicate the Keywords below the abstract, minimum of 5 and maximum of 10 key words

-The funding statement section should appear after the conclusion section

-Ethics statement should appear in the materials and methods section.In the ethics statement,PLOS ONE requires that,for studies reporting research involving human participants,the author(s) should confirm that the specific study was reviewed and approved by an institutional review board (ethics committee) before the study began. Please provide the specific name of the ethics committee/IRB that approved your study, or explain why you did not seek approval in this case.Also,please provide additional details regarding participant consents before data collection.

2. Abstract section

-In the abstract section,please indicate briefly the specific study area(s),sampling methods/technique of the respondents and the sample size, data sources/types (primary or secondary data)and data collection method(whether by interview,questionnaire(cross-sectional or panel data). Also indicate the significance effect levels(eg. at P<0.05(5% etc) for results indicated in the abstract.Meaning that, you should consider to summarize the most important results and their significance levels.

3.Introduction section

-The whole first paragraph in the introduction is not cited,please consider to cite it.

-Again, consider to cite(indicate the sources) the words from line 75 to line 81,from line 84 to line 87 , and from line 98 to line 119 . Please,consider to indicate the sources of all words or sentences which are not yours

4.Materials and Methods section

-Please recheck the model formula,you have indicated that i(i=1,...,m) is the inputs and r(r=1,...,s) is the output,but they do not appear in the formula,consider to recheck it or elaborate more the model formula in detail.

-Restructure(regroup) the materials and methods section to indicate clearly the;

-(i) Description of the specific study area/site.Consider to put a map if possible

-(ii) Sampling procedures of the participants(respondents) together with sample size if applicable

-(iii) Data collection methods and data sources if applicable

-(iv) Analytical models/data analysis models

-(v) Conceptual framework of the study

-(vi) Variables selection and justification supported by literature if available.You can also include the hypothesized effect of variables on bleeding efficiency(optional)

In addition,clarify in this section why you intend to publish 2018 surveyed data in 2020? and not earlier?say 2019.

5.Results and discussion section

-When reporting and discussing the results ,please indicate the significance effect levels of every variables included in your model (eg. X has a positive impact on Y and statistically significant at P<0.05(5%),0.01(10%), etc or otherwise)

-State what other similar studies in other countries have reported(if available)

6.Referencing and citation:

-There are wrong citations in the lines 155,159,164,165,172,173,220,316,318,333, and 488,consider to recheck,please citations should be by numbers and not by year of publication,example[10] and not (Zhu Ning, 2016)

-References number 3,4,5,15,16,17,19,21,22,23,24,30,and 31 are in the reference list but not cited in the manuscript,consider to remove or cite them.In addition reference number 40 and 42 are been cited in line 454 but are not listed in the reference list,consider to add them. For more details visit the citation and referencing guidelines on the PLOS ONE homepage

-You are encouraged to use citation and referencing tools such as End Note to avoid errors.

Reviewer #2: The authors provide first-hand survey data, hoping to prove the research conclusion. The authors have provided data on all the findings in their manuscripts.Two suggestions for amendment.1. Supplement the latest academic literature related to this paper in 2020.2. Add discussion on the research process and conclusion of the paper.

Reviewer #3: I congratulate the authors for spending their time to research into an emerging field. The current paper needs much work to be done.

Abstract does not reflect the main purpose of the study and major findings. The originality is not properly written. In abstract, the authors should present what the conclusions serve for. Clearly demonstrate the objective of this study by adding a paragraph at the end of introduction section.

The introduction should flow from the global understanding or argument around your topic or key variable. Why the study need to be done? What is significance of the research? What is the motivation for this study? What are the specific objectives of the study? What is the potential contribution of this study? None of them are reported in the introduction section. Many mistakes and unclear sentences also appear quite often in the texts, a native English speaker would be helpful in improving the quality of language.

There is no literature review section. At least, the authors could define the research variable and report what had been done by other researchers within their research setting. The author should also demonstrate any research gap in the literature review.

Your methodology is poorly explained and confusing. What is your research paradigm? Why descriptive design? If you are looking at identified factors that influence recreational cost of park visitors, then why descriptive design? Reconsider the research design. Where do you adopt your survey?

The conclusion did not capture the summary of each of the objectives. What new have contributed to existing literature? Where are the recommendations in this paper? The study did not show any of the following implications: theoretical, managerial and practical implications.

Reviewer #4: 1. Generally speaking, mediator should remain independent. However, in the model construction of the influence path study, scale is used as an mediator, and at the same time, the production efficiency calculated by the scale is used as the outcome variable, so that the independence of the mediator variable does not exist, and the result will be unstable.

2. In the influence path study, the time trend is not stripped. That is, in 2010-2017, there will be various exogenous variables, rather than the factors of the policy itself, which will affect the production efficiency of farmers, leading to the effect of the policy being magnified.

3. In Table 1, what are the specific reasons for the positive impact of grassland policy on the scale of household animal husbandry? Is there such a positive effect? Please specify. Does this positive effect really exist? Please be specific.

Reviewer #5: This article discusses an interesting topic. The ecological compensation policy plays an important role in improving China's environment. This article discusses the impact of grassland ecological compensation policy on the efficiency of herders’ breeding technical efficiency. And further, it focuses on the impact mechanism of the policy on the efficiency. The structure of article is reasonable and it can arouse readers' interest. I recommend publishing in its current form.

Reviewer #6: Try to be more specific and clearer of variables measurement and explain the model employed. Please, kindly take time to read the instructions for authors and follow closely the guidelines of the journal. The paper contains a lot of English and grammatical errors. Kindly edit your English. Recheck the authors guide on references and citations and update your paper to fit the journal requirements.

**********

6. PLOS authors have the option to publish the peer review history of their article (what does this mean?). If published, this will include your full peer review and any attached files.

If you choose “no”, your identity will remain anonymous but your review may still be made public.

**Do you want your identity to be public for this peer review?** For information about this choice, including consent withdrawal, please see our Privacy Policy.

Reviewer #1: **Yes: **Cornel Anyisile Kibona

Reviewer #2: No

Reviewer #3: No

Reviewer #4: No

Reviewer #5: No

Reviewer #6: **Yes: **Essiagnon John-Philippe Alavo

[NOTE: If reviewer comments were submitted as an attachment file, they will be attached to this email and accessible via the submission site. Please log into your account, locate the manuscript record, and check for the action link "View Attachments". If this link does not appear, there are no attachment files.]

While revising your submission, please upload your figure files to the Preflight Analysis and Conversion Engine (PACE) digital diagnostic tool, https://pacev2.apexcovantage.com/. PACE helps ensure that figures meet PLOS requirements. To use PACE, you must first register as a user. Registration is free. Then, login and navigate to the UPLOAD tab, where you will find detailed instructions on how to use the tool. If you encounter any issues or have any questions when using PACE, please email PLOS at figures@plos.org. Please note that Supporting Information files do not need this step.

Attachment Submitted filename: PLOS ONE REVIEW.docx Click here for additional data file.

# Author comment: Author response to Decision Letter 0

**DOI:** 10.1371/journal.pone.0249990.r002

10 Mar 2021

Many thanks to the PLOS ONE editor office for sending us the reviewers’ suggestions. We have discussed each suggestion from the reviewers seriously and made corresponding revision, and the following are our responses.Specific information can be viewed in the reply letterSpecific information can be viewed in the response letter

Reviewer #1:

(1) General comments

-The font type should be checked,the font type of the title should be the same with other parts of the manuscript

-Set your computer to English language to avoid mixing up of Chinese and English font type. Example,the commas used in the lines 311,312,and 316, together with the character used in line 527 are in Chinese format,Please consider to change them to English format.

Response: Thank you for this suggestion. We have carefully checked the fonts and ensured that they are consistent.

-The whole manuscript text should be double-spaced when submitting

-Indicate the number on headings and subheadings(example. 1.Introductions,2.Materials and Methods,2.1.Description of the study area etc.

-Indicate the continuous page numbers on the manuscript

Response: Thank you for this suggestion. We have made corresponding amendments as required, including double-spaced, numbering on headings and subheadings, and indicate the continuous page numbers on the manuscript

-The language quality score is 48/60, which is good,but try to recheck for minor improvements.The minor issues to check are; punctuation,article usage,prepositions,singular-plural form,subject-verb agreement,verb form, and avoid unnecessary word capitalization

-Consider replacing the word "herdsmen" with "herders" if applicable in the given context in the whole manuscript(consider uniformity when using the words)

-Consider replacing the word "paper" with "study" if applicable in the given context, or use the preferred word whether the study,paper, or article on the whole manuscript(these words been used in the lines 45,178,218,226,,292,322,3345, and 353)

Response: Thank you for this suggestion. We re-checked the spelling and grammar of the words in the manuscript, and unified the related expressions. We use "study" instead of "paper" and "herders" instead of "herdmens".We also have invited a professional editing group to improve the language.

-Indicate the Keywords below the abstract, minimum of 5 and maximum of 10 key words

-The funding statement section should appear after the conclusion section

Response: Thank you for this suggestion. We added five keywords(Grassland Ecological Compensation and Award Policy;Parallel Mediation Effect Model;Data Envelopment Analysis(DEA);Breeding Efficiency;Inner Mongolia) below the abstract and added the funding statement contents after the conclusion. 

-Ethics statement should appear in the materials and methods section.In the ethics statement,PLOS ONE requires that,for studies reporting research involving human participants,the author(s) should confirm that the specific study was reviewed and approved by an institutional review board (ethics committee) before the study began. Please provide the specific name of the ethics committee/IRB that approved your study, or explain why you did not seek approval in this case.Also,please provide additional details regarding participant consents before data collection.

Response: Thank you for reminding. This herders’ survey was approved and carried out by the School of Economics and Management of Beijing Forestry University and the School of Finance and Economics of Jiangsu University, and was assisted by the local government. The main source of funding was the National Natural Science Foundation of China (No. 71704067). All herdsmen surveyed are aware of the purpose of the survey data. We have added corresponding instructions in the materials and methods section.

(2) Abstract section

-In the abstract section,please indicate briefly the specific study area(s),sampling methods/technique of the respondents and the sample size, data sources/types (primary or secondary data)and data collection method(whether by interview,questionnaire(cross-sectional or panel data). Also indicate the significance effect levels(eg. at P<0.05(5% etc) for results indicated in the abstract.Meaning that, you should consider to summarize the most important results and their significance levels.

Response: Thanks for your suggestions. We have rewritten the summary section, which introduces the source of the data and how it was collected, while supplementing the most important results and their significance levels. The specific description of the survey data will be described in more detail in the data source section(4.1Description of the study area).

(3) Introduction section

-The whole first paragraph in the introduction is not cited,please consider to cite it.

-Again, consider to cite(indicate the sources) the words from line 75 to line 81,from line 84 to line 87 , and from line 98 to line 119 . Please,consider to indicate the sources of all words or sentences which are not yours 

Response: Thanks for your suggestions. We have combined the opinions of multiple reviewers and rewritten the original introduction part. The new content will be divided into two parts: introduction and literature review. Regarding the citation problem you mentioned, we have revised it in the new content and guaranteed to indicate the sources of all words or sentences which are not ours.

(4) .Materials and Methods section

-Please recheck the model formula,you have indicated that i(i=1,...,m) is the inputs and r(r=1,...,s) is the output,but they do not appear in the formula,consider to recheck it or elaborate more the model formula in detail.

Response: Thanks for your suggestions. Sorry, there was an error in our statement,Where is the subscript number of input, is the subscript number of output, the actual input items and output items are represented by x and y.We have modified this part of the content.

-Restructure(regroup) the materials and methods section to indicate clearly the;

-(i) Description of the specific study area/site.Consider to put a map if possible

-(ii) Sampling procedures of the participants(respondents) together with sample size if applicable

-(iii) Data collection methods and data sources if applicable

-(iv) Analytical models/data analysis models

-(v) Conceptual framework of the study

-(vi) Variables selection and justification supported by literature if available.You can also include the hypothesized effect of variables on bleeding efficiency(optional)

In addition,clarify in this section why you intend to publish 2018 surveyed data in 2020? and not earlier?say 2019

Response: Thanks for your suggestions. Combined with the comments of reviewer 3, we rewrite the Data and variables section, which adds a detailed description of the survey visit area.

-(i) (ii) (iii) :The data used in this study comes from a field questionnaire interview on herders in the pastoral area of Inner Mongolia conducted by the research team. from June to November 2018. First, based on the regional characteristics of Inner Mongolia’s grasslands and pastoral areas, the Xinbarag Right(Hulunbeier City), Chenbarag Qi (Hulunbeier City), West Ujimqin (Xilinguole League) and Siziwang (Ulanchabu) were selected as the geographical distribution of the 4 sample counties is shown in the figure1. Then, taking into account the differences in population and grassland area, 4 to 8 sample towns (Sumu) were selected in each sample county, and a questionnaire survey was conducted with herders in the form of "one-to-one" interviews. The questionnaire survey includes the following aspects: basic characteristics of herders households (sex, age, education level, grassland area, etc.); basic household conditions (total population, household income level and structure, etc.); livestock production investment, Livestock breeding expenses, livestock income, living expenses, grassland compensation policy and other subsidies. A total of 449 valid questionnaires were collected in this survey.

Figure 1 Locations of the study area

-(iv) (v) :We have provided a research framework diagram (Fig. 2) in the new submission. Fig.2 briefly and clearly shows the motivation, data sources, Mechanism analysis and modeling steps, Model results and discussion, conclusions of our work.

-In addition,the main reason we did not publish 2018 surveyed data in 2019 is that we did not use this set of data in time. GECP's research on herder farming efficiency is the result of changing herders' breeding behavior. Before this study, we paid more attention to the changes of herdsmen's breeding behavior before and after the implementation of the policy.

Figure 2 The research framework diagram.

-(vi): We rewrote the methods and data parts, and gave a detailed introduction to the selected methods and selected variables.

(5) Results and discussion section

-When reporting and discussing the results ,please indicate the significance effect levels of every variables included in your model (eg. X has a positive impact on Y and statistically significant at P<0.05(5%),0.01(10%), etc or otherwise)

-State what other similar studies in other countries have reported(if available)

Response: Thank you for your suggestion. We have marked the significance level of each variable in the results section of the resubmitted manuscript. Because GECP is implemented in China, although there are similar ecological compensation policies in the world, the implementation process and reward methods are quite different from those of GECP and are not comparable. At the same time, the related research rarely involves the impact on the breeding efficiency of herdsmen. We have selected two articles by Chinese scholars to support our conclusion (Liu et al. (2019) and Han et al. (2019)).

(5) Referencing and citation:

-There are wrong citations in the lines 155,159,164,165,172,173,220,316,318,333, and 488,consider to recheck,please citations should be by numbers and not by year of publication,example[10] and not (Zhu Ning, 2016)

-References number 3,4,5,15,16,17,19,21,22,23,24,30,and 31 are in the reference list but not cited in the manuscript,consider to remove or cite them.In addition reference number 40 and 42 are been cited in line 454 but are not listed in the reference list,consider to add them. For more details visit the citation and referencing guidelines on the PLOS ONE homepage

-You are encouraged to use citation and referencing tools such as End Note to avoid errors.

Response: Thank you for your suggestion. We carefully refer to the citations and reference guide on the PLOS ONE homepage and revise the citations of the manuscript with the help of ENDnote.

Reviewer #2:

The authors provide first-hand survey data, hoping to prove the research conclusion. The authors have provided data on all the findings in their manuscripts.Two suggestions for amendment.

1. Supplement the latest academic literature related to this paper in 2020.

Response: Thanks for your suggestions. We have supplemented the latest relevant academic literature in 2020 as much as possible in the article.

2. Add discussion on the research process and conclusion of the paper.

Response: Thanks for your suggestions. We have added a description of the research process of the article to the newly submitted manuscript. First, we used the DEA method to measure the efficiency of herder farming in 2010 and 2017, and then used the package in R: "lavan" and "Mediation" calculates the mediation effect model, in which farming scale, farming structure and pasture circulation are used as mediating variables. At the same time, we divided herders into large-scale and small-scale, and discussed the heterogeneity of the impact of GECP on herders' breeding efficiency.

Reviewer #3: I congratulate the authors for spending their time to research into an emerging field. The current paper needs much work to be done.

Abstract does not reflect the main purpose of the study and major findings. The originality is not properly written. In abstract, the authors should present what the conclusions serve for.

Response: Thanks for this suggestion. We have rewritten the abstract in the new submission. In the new abstract, we elaborated on the motivation, method, result of this article. The main purpose of this research is to answer whether the implementation of the GECP policy can improve the grassland The breeding efficiency of herders, and the effect path of scale changes, grassland circulation behavior and changes in breeding structure are analyzed. The main conclusion drawn is that the total effect of the grassland ecological compensation policy on the breeding efficiency of Inner Mongolia grassland herdsmen is a positive effect (P<0.01), and the change of breeding methods (direct effect) is the main influence path, and the grassland circulation behavior (P<0.01) and the scale of breeding (P<0.01) are part of the mediating effect, and the mediating effect of the breeding structure is not significant (P>0.1).

Clearly demonstrate the objective of this study by adding a paragraph at the end of introduction section.The introduction should flow from the global understanding or argument around your topic or key variable. Why the study need to be done? What is significance of the research? What is the motivation for this study? What are the specific objectives of the study? What is the potential contribution of this study? None of them are reported in the introduction section. Many mistakes and unclear sentences also appear quite often in the texts, a native English speaker would be helpful in improving the quality of language.

Response: Thanks for this suggestion. We have rewritten the introduction part based on the opinions of you and reviewer 1, which describes the research purpose and significance of this article.The main purpose of this research is to answer whether the implementation of the GECP policy can improve the grassland The breeding efficiency of herders, and the effect path of scale changes, grassland circulation behavior and changes in breeding structure are analyzed. The main conclusion drawn is that the total effect of the grassland ecological compensation policy on the breeding efficiency of Inner Mongolia grassland herdsmen is a positive effect (P<0.01), and the change of breeding methods (direct effect) is the main influence path, and the grassland circulation behavior (P<0.01) and the scale of breeding (P<0.01) are part of the mediating effect, and the mediating effect of the breeding structure is not significant (P>0.1). 

In the literature review section, we propose the marginal contribution of this research.This study makes the following contributions on the basis of previous studies: 1) enrich the research of GECP on livestock breeding efficiency, and study the impact of policies on livestock breeding efficiency from a micro perspective based on the survey data of Inner Mongolia Pastoral Areas; 2) establish a quantitative model to analyze the impact of GECP on the technical efficiency of herders’ breeding, because GECP changes the efficiency by influencing herders’ breeding behavior. Therefore, the parallel mediating effect model was utilized to analyze the influence mechanism of GECP on aquaculture efficiency from three action paths: breeding scale, breeding structure and grassland circulation.

There is no literature review section. At least, the authors could define the research variable and report what had been done by other researchers within their research setting. The author should also demonstrate any research gap in the literature review.

Response: Thanks for this suggestion. We have combined the opinions of multiple reviewers and rewritten the original introduction part. The new content will be divided into two parts: introduction and literature review. Reviewing the previous literature on the implementation effect of grassland ecological compensation policy, it can be summarized into two aspects. On the one hand, the impact of GECP on grassland ecological environment, that is, ecological effect, mainly focuses on the changes of the thickness of the grass layer of natural grassland, grassland coverage and biomass before and after the implementation of GECP. Based on the perspective of herders’ animal husbandry production, another part of the works studies the impact of GECP, that is, economic effect. However, there are two shortcomings in this kind of studies: firstly, the data adopted is open and macro; secondly, this kind of studies only compare the breeding efficiency prior to and after the implementation of GECP, but do not conduct the precise estimate through metering model or do not analyze the effecting mechanisms.

Therefore, this study makes the following contributions on the basis of previous studies: 1) enrich the research of GECP on livestock breeding efficiency, and study the impact of policies on livestock breeding efficiency from a micro perspective based on the survey data of Inner Mongolia Pastoral Areas; 2) establish a quantitative model to analyze the impact of GECP on the technical efficiency of herders’ breeding, because GECP changes the efficiency by influencing herders’ breeding behavior. Therefore, the parallel mediating effect model was utilized to analyze the influence mechanism of GECP on aquaculture efficiency from three action paths: breeding scale, breeding structure and grassland circulation.

Your methodology is poorly explained and confusing. What is your research paradigm? Why descriptive design? If you are looking at identified factors that influence recreational cost of park visitors, then why descriptive design? Reconsider the research design. 

Response: Thanks for this suggestion. We have provided a research framework diagram (Fig. 2) in the new submission. Fig.2 briefly and clearly shows the motivation, data sources, Mechanism analysis and modeling steps, Model results and discussion, conclusions & Suggestions of our work. I'm sorry that I don't understand the "entertainment cost affecting park visitors". If you are not satisfied with our modification, we welcome your suggestions for further modification. Thank you very much.

Figure 3 The research framework diagram.

Where do you adopt your survey?

Response: The data used in this study comes from a field questionnaire interview on herders in the pastoral area of Inner Mongolia conducted by the research team. from June to November 2018. First, based on the regional characteristics of Inner Mongolia’s grasslands and pastoral areas, the Xinbarag Right(Hulunbeier City), Chenbarag Qi (Hulunbeier City), West Ujimqin (Xilinguole League) and Siziwang (Ulanchabu) were selected as the geographical distribution of the 4 sample counties is shown in the figure. Then, taking into account the differences in population and grassland area, 4 to 8 sample towns (Sumu) were selected in each sample county, and a questionnaire survey was conducted with herders in the form of "one-to-one" interviews. The questionnaire survey includes the following aspects: basic characteristics of herders households (sex, age, education level, grassland area, etc.); basic household conditions (total population, household income level and structure, etc.); livestock production investment, Livestock breeding expenses, livestock income, living expenses, grassland compensation policy and other subsidies. A total of 449 valid questionnaires were collected in this survey.

The conclusion did not capture the summary of each of the objectives. What new have contributed to existing literature? Where are the recommendations in this paper? The study did not show any of the following implications: theoretical, managerial and practical implications.

Response: Thank you for your suggestion. We have rewritten the introduction, literature review and conclusion in the newly submitted manuscript. In the Literature review section we pointed out the contribution of this research：1) enrich the research of GECP on livestock breeding efficiency, and study the impact of policies on livestock breeding efficiency from a micro perspective based on the survey data of Inner Mongolia Pastoral Areas; 2) establish a quantitative model to analyze the impact of GECP on the technical efficiency of herders’ breeding, because GECP needs to influence herders’ breeding behavior. At the end of this article, we rewrote the policy recommendations part, combined with the research conclusions of this article, we put forward three recommendations: 1)While continuing to implement the current grassland ecological protection compensation policy, Chinese government should formulate corresponding supporting policies to support the transformation and development of livestock husbandry in pastoral areas through technology and funds, such as the policy support for the construction of breeding land and for transforming beef cattle grazing to house breeding, forming a joint force with the ecological compensation policy to promote the transformation ;2)the government should actively establish corresponding grassland circulation platform to avoid grassland waste and improve resource utilization efficiency;3) improving the breeding level of herders by means of technical training is helpful to further enhance the economic benefits of livestock husbandry, and will contribute to the professional and modern development of animal husbandry

Reviewer #4:

1. Generally speaking, mediator should remain independent. However, in the model construction of the influence path study, scale is used as an mediator, and at the same time, the production efficiency calculated by the scale is used as the outcome variable, so that the independence of the mediator variable does not exist, and the result will be unstable.

Response:Thank you very much for your valuable suggestion which we have not considered. Next, I will elaborate on the following three points: First, due to our misrepresentation, the output variable that we estimate the efficiency of breeding is: the Total outcome Livestock number (standard sheep), this variable is not consistent with scale (farming scale) , According to our understanding the Total Livestock number (standard sheep)=scale *the ratio of outgoing stocks, but we agree with your statement "the production efficiency calculated by the scale is used as the outcome variable", because Y and scale have a strong correlation; Second, in the study, we conducted a discussion on the heterogeneity of herders of different sizes, and divided herders into large-scale herders and small-scale herders according to the median of the sample size in 2011, and established mediation effects respectively.The model is estimated. From the results of the model, the two types of herdsmen are not affected by the GECP policy. Therefore, we believe that our results are robust. Finally, in order to further ensure the accuracy of the results, we have The output variables were standardized. The output variables were divided by the slaughter amount to 1, and the input variables were changed to the area, lab, feed, and other required to produce 1 unit of standard sheep. The efficiency was re-estimated (average breeding in 2010). The efficiency was 0.2760, and the average breeding efficiency in 2017 was 0.2911), and the mediation effect model was established to obtain the following results: It can be found that the sign and significance of the core explanatory variables (policy) in each equation has not changed significantly. The final mediating effect path analysis can find that the direct effect is positive and accounted for 76.17%, and the indirect effect is positive and accounted for 23.86%, which is not much different from the original results (ADE, 82.22%; ACME, 17.78%), so it is believed that eliminating the impact of scale did not cause a major change in the results.

Table 1 mediation model results (calculation efficiency after data standardization)

　 Dependent variable:

 Larea scale farmstr eff

scale 　 　 　 0.051***

　 　 　 　 (-0.008)

farmstr 　 　 　 0.003

　 　 　 　 (-0.029)

Larea 　 　 　 0.048***

　 　 　 　 (-0.012)

policy 6.584*** 8.893*** 0.512 2.442***

　 (-0.825) (-1.211) (-0.34) （-0.317）

inc 0.001 -0.001 0.002*** -0.002***

　 (-0.001) (-0.002) (-0.001) （-0.0004）

incstr 0.161 0.29 -0.011 -0.064

　 (-0.172) (-0.252) (-0.071) （-0.062）

price -0.001 0.002 0.001 -0.002**

　 (-0.004) (-0.006) (-0.002) （-0.001）

age 0.004*** -0.001 0.0002 0.001***

　 (-0.001) (-0.002) (-0.001) （-0.0005）

edu -0.034** -0.008 -0.003 -0.003

　 (-0.015) (-0.022) (-0.006) （-0.005）

lab 0.022* -0.005 -0.012*** 0.005

　 (-0.011) (-0.017) (-0.005) （-0.004）

dis1 0.125** -0.046 -0.035* 0.006

　 (-0.05) (-0.073) (-0.02) （-0.018）

dis2 -0.065 （-0.031） -0.139*** 0.018

　 （-0.066） （-0.097） -0.027 （-0.024）

Constant 309.834*** 444.257*** 23.976 129.121***

　 （-44.99） （-65.987） （-18.516） （-17.032）

Year Yes Yes Yes Yes

Firm Yes Yes Yes Yes

R2 0.141 0.634 0.1 0.621

F Statistic 13.170*** 139.598*** 8.989*** 103.497*** 

*** p<0.01, ** p<0.05, * p<0.1

 Theoretical path coefficients Path coefficients Total path coefficient

(effect ratio)

ADE

（Direct effect） 

2.442***

(0.312) 2.442***

（76.17%）

ACME

(Indirect effect) Policy→Structure→EFF 

0.002

(0.015) 0.765***

（23.86%）

 Policy→Scale→EFF 

0.449***

(0.088) 

 Policy→Circulation→

（ADE）/Scale(ACME)→EFF 

0.314***

(0.094) 

ATE

（Total effect） 4.561***

(0.303) 3.206***

（100%）

*** p<0.01, ** p<0.05, * p<0.1

Based on the above three points, we believe that the results of this study are robust and credible. Thank you again for pointing out the points that we have not noticed. If you are not satisfied with our modification or have other question, we welcome your suggestions for further modification. Thank you very much.

2. In the influence path study, the time trend is not stripped. That is, in 2010-2017, there will be various exogenous variables, rather than the factors of the policy itself, which will affect the production efficiency of farmers, leading to the effect of the policy being magnified.

Response: Thank you for your suggestion. We very much agree with your opinion. Before the start of this study, we tried to consider using the PSM-DID model to eliminate common trends in time. However, since the GECP policy covers almost all herder families, there is almost no control Group (ie the control group not affected by GECP) for comparison; However, this study refers to related research trying to control the influence of GECP and other factors by using a fixed-effects model, which can Control most of the time-varying variables, including livestock prices, household factors and climate factors, to obtain an accurate estimate of GECP's breeding efficiency for herders.

3. In Table 1, what are the specific reasons for the positive impact of grassland policy on the scale of household animal husbandry? Is there such a positive effect? Please specify. Does this positive effect really exist? Please be specific.

Response: Thank you for your suggestion. In response to your questions, I will answer them one by one: I think that the GECP policy has a positive effect on the increase of herd farming scale. We quote macro data to prove this point. Table 3 records the Average number of rearing per household, It can be found that this value has increased. The specific reasons will be explained from the following two aspects:

Table 2 Average number of rearing per household from 2011 to 2017

Year 2011 2012 2013 2014 2015 2016 2017

Number of herders households in Inner Mongolia pastoral area (ten households) 48.87 48.45 55.50 57.28 57.33 57.67 56.11 

Number of sheep and goat in Inner Mongolia pastoral area at the end of the year (ten thousand) 5276.00 5144.00 5239.20 5569.28 5777.80 5506.24 6111.93 

Average number of rearing per household 107.96 106.17 94.40 97.23 100.79 95.48 108.92 

1：The purpose of the GECP policy itself is not to reduce the scale of pastoral farming: the purpose of the GECP policy itself is to protect the grassland ecology in China and promote the transformation and upgrading of animal husbandry. It does not mean that the implementation of the policy is to reduce the scale of herdsmen’s breeding, and reducing the intensity of pasture utilization is not the same as reducing the scale of herders’ breeding, because herders can switch to shed-raising methods or rent out herdsmen who give up livestock production. Expansion of the pasture. We used two data to prove this statement: one is the value of fixed assets produced by herdsmen in Inner Mongolia at the end of the year (data source: "Inner Mongolia Statistical Yearbook") and the average amount of food consumed to raise a sheep (data source: "Compilation of National Agricultural Product Cost and Benefit Data"), it can be found that these two values have fluctuated and increased from 2011 to 2017. Therefore, we believe that herders are gradually changing traditional grazing and feeding methods, and the reduction in the intensity of pasture utilization does not mean the expansion of herdsmen’s farming scale.

Figure 4 Average value of fixed assets produced by herdsmen at the end of the year($ in 2015)

Table 3 Quantity of food consumed by sheep from 2011 to 2017

 Year unit sheep goat

Quantity of food consumed per 100 2011 kg 816.66 2584.45

Quantity of food consumed per 101 2012 kg 1004.27 2790.63

Quantity of food consumed per 102 2013 kg 847.24 3616.24

Quantity of food consumed per 103 2014 kg 1077.77 3867.56

Quantity of food consumed per 104 2015 kg 1208.54 3401.42

Quantity of food consumed per 105 2016 kg 1218.28 4197.59

Quantity of food consumed per 106 2017 kg 1267.62 4077.75

2 . GECP's own subsidies and supervision are not very strong. As a rational person, herders have more economic rationality than ecological rationality. After receiving supplementary funds, they are more inclined to use funds for the expansion of production scale, which has led to grassland ecology to a certain extent. The compensation policy for environmental protection is ineffective; Secondly, the compensation for the grassland ecological compensation policy is low, and at the same time, the implementation of policy supervision is difficult, and illegal grazing often occurs(Hu Yuanning, 2019).

Reviewer #5: 

This article discusses an interesting topic. The ecological compensation policy plays an important role in improving China's environment. This article discusses the impact of grassland ecological compensation policy on the efficiency of herders’breeding technical efficiency. And further, it focuses on the impact mechanism of the policy on the efficiency. The structure of article is reasonable and it can arouse readers' interest. I recommend publishing in its current form

Response: Thanks for your postive comments.

Reviewer #6: 

Try to be more specific and clearer of variables measurement and explain the model employed. Please, kindly take time to read the instructions for authors and follow closely the guidelines of the journal. The paper contains a lot of English and grammatical errors. Kindly edit your English. Recheck the authors guide on references and citations and update your paper to fit the journal requirements.

Response: Thanks for your suggestions. We rewrote the methods and data parts, and gave a detailed introduction to the selected methods and selected variables. We followed the requirements of the journal to review the unreasonable places in the paper, and invited a professional English editing team to improve the language. If you are not satisfied with our modification or have other question, we welcome your suggestions for further modification. Thank you very much.

Attachment Submitted filename: Response to Reviewers.docx Click here for additional data file.

# Peer review: Decision Letter 1

**DOI:** 10.1371/journal.pone.0249990.r003

**Authors:** Bing Xue

30 Mar 2021

Does the Grassland Ecological Compensation Policy improve the herders' breeding technical efficiency in China?—Based on the parallel mediation effect model

PONE-D-20-31234R1

Dear Dr. Li,

We’re pleased to inform you that your manuscript has been judged scientifically suitable for publication and will be formally accepted for publication once it meets all outstanding technical requirements.

Within one week, you’ll receive an e-mail detailing the required amendments. When these have been addressed, you’ll receive a formal acceptance letter and your manuscript will be scheduled for publication.

An invoice for payment will follow shortly after the formal acceptance. To ensure an efficient process, please log into Editorial Manager at http://www.editorialmanager.com/pone/, click the 'Update My Information' link at the top of the page, and double check that your user information is up-to-date. If you have any billing related questions, please contact our Author Billing department directly at authorbilling@plos.org.

If your institution or institutions have a press office, please notify them about your upcoming paper to help maximize its impact. If they’ll be preparing press materials, please inform our press team as soon as possible -- no later than 48 hours after receiving the formal acceptance. Your manuscript will remain under strict press embargo until 2 pm Eastern Time on the date of publication. For more information, please contact onepress@plos.org.

Kind regards,

Bing Xue, Ph.D.

Academic Editor

PLOS ONE

Additional Editor Comments (optional):

Reviewers' comments:

Reviewer's Responses to Questions

**Comments to the Author**

1. If the authors have adequately addressed your comments raised in a previous round of review and you feel that this manuscript is now acceptable for publication, you may indicate that here to bypass the “Comments to the Author” section, enter your conflict of interest statement in the “Confidential to Editor” section, and submit your "Accept" recommendation.

Reviewer #1: (No Response)

Reviewer #3: All comments have been addressed

Reviewer #4: All comments have been addressed

Reviewer #6: All comments have been addressed

**********

2. Is the manuscript technically sound, and do the data support the conclusions?

The manuscript must describe a technically sound piece of scientific research with data that supports the conclusions. Experiments must have been conducted rigorously, with appropriate controls, replication, and sample sizes. The conclusions must be drawn appropriately based on the data presented. 

Reviewer #1: Yes

Reviewer #3: Yes

Reviewer #4: Yes

Reviewer #6: Yes

**********

3. Has the statistical analysis been performed appropriately and rigorously? 

Reviewer #1: Yes

Reviewer #3: Yes

Reviewer #4: Yes

Reviewer #6: Yes

**********

4. Have the authors made all data underlying the findings in their manuscript fully available?

The PLOS Data policy requires authors to make all data underlying the findings described in their manuscript fully available without restriction, with rare exception (please refer to the Data Availability Statement in the manuscript PDF file). The data should be provided as part of the manuscript or its supporting information, or deposited to a public repository. For example, in addition to summary statistics, the data points behind means, medians and variance measures should be available. If there are restrictions on publicly sharing data—e.g. participant privacy or use of data from a third party—those must be specified.

Reviewer #1: Yes

Reviewer #3: Yes

Reviewer #4: Yes

Reviewer #6: Yes

**********

5. Is the manuscript presented in an intelligible fashion and written in standard English?

PLOS ONE does not copyedit accepted manuscripts, so the language in submitted articles must be clear, correct, and unambiguous. Any typographical or grammatical errors should be corrected at revision, so please note any specific errors here.

Reviewer #1: No

Reviewer #3: Yes

Reviewer #4: Yes

Reviewer #6: Yes

**********

6. Review Comments to the Author

Please use the space provided to explain your answers to the questions above. You may also include additional comments for the author, including concerns about dual publication, research ethics, or publication ethics. (Please upload your review as an attachment if it exceeds 20,000 characters)

Reviewer #1: -The authors have tried to address some comments raised in a previous round of review and the manuscript has been improved. However, the authors did not check the English stricture and grammar of some sentences in the manuscript as addressed in previous comments.

-I recommend the manuscript to be accepted for publication, however minor modifications should be made prio to publication.

-I have added all the comments in the attached manuscript file. Please download the file and check all the comments addressed to improve your work.

-Note: English grammar of your manuscript should be checked by English expert.

Reviewer #3: Manuscript technically sound, and do the data support the conclusions.

Statistical analysis been performed appropriately and rigorously.

Manuscript presented in an intelligible fashion and written in standard English

Reviewer #4: (No Response)

Reviewer #6: All the review comments were addressed and the manuscript was improved. However, there are still minor things to consider before getting the manuscipt ready for publication. These are English language improvement, grammar spelling and minor mistakes. For instance "the use of herders and herdersmen" etc..

**********

7. PLOS authors have the option to publish the peer review history of their article (what does this mean?). If published, this will include your full peer review and any attached files.

If you choose “no”, your identity will remain anonymous but your review may still be made public.

**Do you want your identity to be public for this peer review?** For information about this choice, including consent withdrawal, please see our Privacy Policy.

Reviewer #1: **Yes: **Cornel Anyisile Kibona

Reviewer #3: **Yes: **Sehresh Hena

Reviewer #4: No

Reviewer #6: **Yes: **Essiagnon John-Philippe Alavo

Attachment Submitted filename: Manuscript (4).docx Click here for additional data file.

Attachment Submitted filename: Reviewed Manuscript in PDF.pdf Click here for additional data file.

# Editor report: Acceptance letter

**DOI:** 10.1371/journal.pone.0249990.r004

**Authors:** Bing Xue

13 Apr 2021

PONE-D-20-31234R1 

Does the Grassland Ecological Compensation Policy Improve the Herders' Breeding Technical Efficiency in China?—Based on the parallel mediation effect model 

Dear Dr. Li:

I'm pleased to inform you that your manuscript has been deemed suitable for publication in PLOS ONE. Congratulations! Your manuscript is now with our production department. 

If your institution or institutions have a press office, please let them know about your upcoming paper now to help maximize its impact. If they'll be preparing press materials, please inform our press team within the next 48 hours. Your manuscript will remain under strict press embargo until 2 pm Eastern Time on the date of publication. For more information please contact onepress@plos.org.

If we can help with anything else, please email us at plosone@plos.org. 

Thank you for submitting your work to PLOS ONE and supporting open access. 

Kind regards, 

PLOS ONE Editorial Office Staff

on behalf of

Professor Bing Xue 

Academic Editor

PLOS ONE
